# In-depth proteomic analysis of a mollusc shell: acid-soluble and acid-insoluble matrix of the limpet *Lottia gigantea*

**DOI:** 10.1186/1477-5956-10-28

**Published:** 2012-06-13

**Authors:** Karlheinz Mann, Eric Edsinger-Gonzales, Matthias Mann

**Affiliations:** 1 Abteilung Proteomics und Signaltransduktion, Max-Planck-Institut für Biochemie, Am Klopferspitz 18, D-82152, Martinsried, Munich, Germany; 2 Department of Molecular and Cell Biology, University of California, Berkeley, 545 Life Sciences Addition, Berkeley, CA, 94720, USA

## Abstract

**Background:**

Invertebrate biominerals are characterized by their extraordinary functionality and physical properties, such as strength, stiffness and toughness that by far exceed those of the pure mineral component of such composites. This is attributed to the organic matrix, secreted by specialized cells, which pervades and envelops the mineral crystals. Despite the obvious importance of the protein fraction of the organic matrix, only few in-depth proteomic studies have been performed due to the lack of comprehensive protein sequence databases. The recent public release of the gastropod *Lottia gigantea* genome sequence and the associated protein sequence database provides for the first time the opportunity to do a state-of-the-art proteomic in-depth analysis of the organic matrix of a mollusc shell.

**Results:**

Using three different sodium hypochlorite washing protocols before shell demineralization, a total of 569 proteins were identified in *Lottia gigantea* shell matrix. Of these, 311 were assembled in a consensus proteome comprising identifications contained in all proteomes irrespective of shell cleaning procedure. Some of these proteins were similar in amino acid sequence, amino acid composition, or domain structure to proteins identified previously in different bivalve or gastropod shells, such as BMSP, dermatopontin, nacrein, perlustrin, perlucin, or Pif. In addition there were dozens of previously uncharacterized proteins, many containing repeated short linear motifs or homorepeats. Such proteins may play a role in shell matrix construction or control of mineralization processes.

**Conclusions:**

The organic matrix of *Lottia gigantea* shells is a complex mixture of proteins comprising possible homologs of some previously characterized mollusc shell proteins, but also many novel proteins with a possible function in biomineralization as framework building blocks or as regulatory components. We hope that this data set, the most comprehensive available at present, will provide a platform for the further exploration of biomineralization processes in molluscs.

## Background

Molluscan shells are extraordinarily stable biocomposites of calcium carbonate and an organic matrix consisting of polysaccharides and proteins. The organic matrix, although constituting a very minor fraction of the biocomposite by weight, is thought to be of utmost importance for the construction of the biocomposite and its final properties because it controls crystal nucleation, crystal growth, crystal shape and choice of calcium carbonate polymorph [1,2]. Previously established methods to identify new mollusc shell matrix proteins, such as isolation by chromatography and biochemical characterization or molecular biology approaches, have been complemented recently by mass spectrometry-based proteomic analysis or combination of proteomic and transcriptomic studies [3-11]. However, proteomic approaches depend on the comparison of experimentally determined spectra with theoretical spectra obtained by *in silico* digestion of proteins and *in silico* fragmentation of resulting peptides [12,13]. Therefore protein sequence databases that are as comprehensive as possible, usually derived from genome sequencing, are presently indispensable for high-throughput proteomics. The need for a comprehensive database is highlighted by previously published proteomic studies of shell matrices in various molluscan species [3-11]. These studies relied on translated EST databases contributed by a number of groups [7,11,14-18] and usually less than 15 proteins were identified from isolated organic matrices. Sometimes database searches were combined with *de novo* mass spectrometric sequencing. However, *de novo* sequencing algorithms, which attempt to interpret spectra independently of a sequence database [19], are not compatible with high-throughput analysis at present. Transcriptomics, on the other hand, does not identify matrix proteins directly, making additional techniques, such as immunohistochemical localization, necessary to demonstrate the actual location of potential shell matrix proteins. Thus, although previous studies have identified several very interesting new matrix proteins, these studies may fail to show the actual complexity of the shell matrix proteome indicated by proteomic studies of biomineral matrices of organisms with sequenced genomes, such as chicken [20] or the sea urchin *Strongylocentrotus purpuratus*[21-23].

The first genome sequence of a mollusc, the limpet *Lottia gigantea*, was made public recently (http://genome.jgi-psf.org/Lotgi1/Lotgi1.download.html) [24]. In the present report we used a protein sequence database derived from this genome sequence to perform a high-throughput in-depth proteomic analysis of the shell matrix of this marine snail.

The shell of *Lottia* and related limpets consists of five layers [25,26], which are divided into 3 outer layers, M + 1, M + 2 and M + 3 and separated from an inner layer M-1 by the intermediate myostracum (M layer). The outermost layer, M + 3, is reported to contain calcite as mineral phase. This layer appears eroded and often disappears altogether around the top of the shell. The M + 2 layer consists of flat prismatic crystals made of aragonite, another common calcium carbonate mineral. The M + 1 and M-1 layers are described to consist of lamellar prisms similarly made of aragonite. Compared to the other layers, the M layer, sandwiched between M + 1 and M-1, is very thin and has a prismatic structure of aragonite. Organic matrix was visible in M + 3 and M + 2, but was not detected in other layers [25].

Using LTQ Orbitrap Velos high-performance mass spectrometers [27] in combination with the MaxQuant software package designed for analysis of large high-resolution mass spectrometric data sets [28-30] we identified 311 proteins in the organic matrix of the *Lottia* shell with very high stringency. This is the first in-depth proteomic study of a mollusc shell matrix.

## Materials and methods

The shells of freshly collected limpets were carefully cleaned manually and treated with sodium hypochlorite solution (Merck, Darmstadt; Germany; 6–14% active chlorine) to remove organic surface contaminants. Shells were either treated with hypochlorite for 2 h at room temperature (A), for 2 h with two 5 min ultrasonic treatments at the start of each hour (B), or for 24 h with two 5 min ultrasound bursts as before and one after 24 h (C). The shells were then washed with de-ionized water, dried, and crushed into small pieces using a hammer. The pieces were demineralized in 50% acetic acid (20 ml/g of shell) in a cold room overnight, yielding a dark brown suspension. Acid-soluble and acid-insoluble matrix was separated by centrifugation at 14000g_av_ at 5°C for 1 h. The pellet was washed twice by re-suspension in approximately 20 volumes of 50% acetic acid, centrifugation for 30 min at 14000g_av_, and lyophilized. The supernatant was dialyzed twice against 10 volumes of 10% acetic acid followed by three times 10 volumes of 5% acetic acid at 4–6°C (Spectra/Por 6 dialysis membrane, molecular weight cut-off 2000; Spectrum Europe, Breda, The Netherlands), and lyophilized.

SDS-PAGE was done using pre-cast 4–12% Novex Bis-Tris gels in MES buffer with reagents and protocols supplied by the manufacturer (Invitrogen, Carlsbad, CA). Samples were suspended in 30 μl sample buffer/200 μg of organic matrix and heated to 95°C for 5 min. Sample buffer-insoluble matrix was removed by centrifugation in an Eppendorf bench top centrifuge for 5 min at 13000 rpm. Gels were loaded with 30 μl of matrix sample supernatant per lane and stained with colloidal Coomassie (Invitrogen) after electrophoresis. The protein standard used for molecular weight estimation was Novex Sharp, pre-stained (Invitrogen). Gels were sliced into 12 sections for in-gel digestion with trypsin [31]. The eluted peptides were purified on C18 Stage Tips [32].

Peptide mixtures were analyzed by on-line nanoflow liquid chromatography using the EASY-nLC system (Proxeon Biosystems, Odense, Denmark; now Thermo Fisher) with 15 cm capillary columns of an internal diameter of 75 μm filled with 3 μm Reprosil-Pur C18-AQ resin (Dr. Maisch GmbH, Ammerbuch-Entringen, Germany). The gradient consisted of 5–30% acetonitrile in 0.5% acetic acid at a flow rate of 250 nl/min for 85 min, 30–60% acetonitrile in 0.5% acetic acid at a flow rate of 250 nl/min and 60–80% acetonitrile in 0.5% acetic acid at a flow rate of 250 nl/min for 7 min. The eluate was electrosprayed into an LTQ Orbitrap Velos (Thermo Fisher Scientific, Bremen, Germany) through a Proxeon nanoelectrospray ion source. The Orbitrap Velos was operated in a HCD top 10 mode essentially as described [Olsen et al., 2009] at a resolution of 30,000 for full scans and of 7,500 (both at m/z 400) for MS/MS scans.

Data analysis was performed with MaxQuant (v1.1.1.36) [28,29], a computational proteomics platform based on the Andromeda search engine [30] (http://www.maxquant.org/), using the Lotgi1_GeneModels_Filtered Models1_aa.fasta.gz protein sequence database comprising 23,851 gene models at present (http://genome.jgi-psf.org/Lotgi1/Lotgi1.download.html) [24], together with the corresponding reversed database and the sequences of common contaminants, including human keratins from IPIhuman. Carbamidomethylation was set as fixed modification. Variable modifications were set as oxidation (M), N-acetyl (protein) and pyro-Glu/Gln (N-term). Initial peptide mass tolerance was set to 7 ppm and fragment mass tolerance was 20 ppm. Two missed cleavages were allowed and the minimal length required for peptide identification was seven amino acids. The peptide and protein false discovery rates (FDR) were both set to 0.01. The maximal posterior error probability (PEP) for peptides, which is the probability of each peptide to be a false hit considering identification score and peptide length [28,29], was set to 0.01. The Re-quantify and Second Peptide [30] options were enabled. At least two MaxQuant group sequence-unique peptides with a score >100 were required for protein identification. Furthermore, identifications were only accepted if the peptides were identified in at least two replicates within the respective group A, B or C. Identifications with only two unique peptides were manually validated considering the assignment of major peaks, occurrence of uninterrupted y- or b-ion series of at least 4 consecutive amino acids, preferred cleavages N-terminal to proline bonds, the possible presence of a2/b2 ion pairs and immonium ions, and mass accuracy. The ProteinProspector MS-Product program (http://prospector.ucsf.edu/) was used to calculate the theoretical masses of fragments of identified peptides for manual validation. BLAST and FASTA searches against non-redundant databases (all organisms) were performed using the programs provided by NCBI (http://www.ncbi.nlm.nih.gov/blast) and EBI http://www.ebi.ac.uk/Tools/sss/. Domains were predicted with InterProScan (http://www.ebi.ac.uk/Tools/pfa/iprscan/) and PROSITE (http://prosite.expasy.org/). For sequence alignments we employed Kalign (http://www.ebi.ac.uk/Tools/msa/kalign/) and ClustalW (http://www.ebi.ac.uk/Tools/msa/clustalw2/). Sequence repeats were predicted using RADAR (http://www.ebi.ac.uk/Tools/Radar/index.html). The abundance of proteins was estimated by calculating the exponentially modified protein abundance index (emPAI) [33]. Observable peptides were determined and counted with Protein Prospector (http://prospector.ucsf.edu/prospector/cgi-bin/msform.cgi? form = msdigest) using zero miss-cleavages, a peptide mass of 700–2800, and a minimal peptide length of seven amino acids. Observed unique parent ions with a minimal length of seven amino acids and a mass between 700–2800 used for emPAI calculation included ions with up to two miss-cleavages, modifications specified for MaxQuant analysis (see above), different charges, and neutral losses [33]. Proteins with emPAI ≥9 were referred to as major proteins in this report.

## Results and discussion

### Matrix isolation and characterization by SDS-PAGE

**Figure 1:**
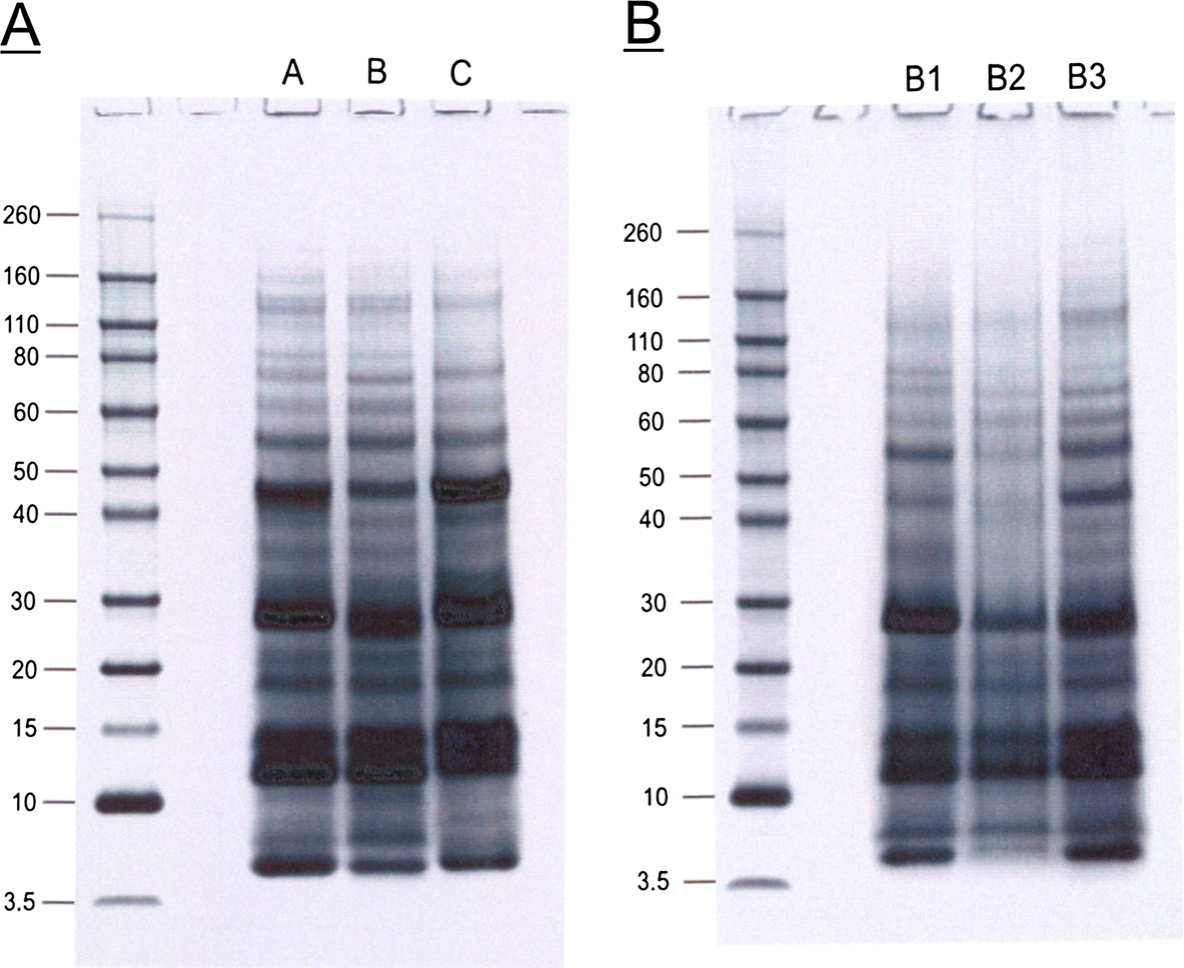
**PAGE comparison of acid-soluble matrices from shells.** Molecular weight markers are indicated at the left. Each lane was loaded with 200 μg of matrix in a volume of 30 μl. **A**, matrices of shells cleaned with different sodium hypochlorite protocols. Lane A, 2 h hypochlorite at room temperature; lane B, 2 h hypochlorite with 2 x 5 min ultrasound treatment at the start of each hour; lane C, cleaned with hypochlorite for 24 h with 2 x 5 min ultrasound bursts as before and one after 24 h. **B**, matrices of different shells, all cleaned with hypochlorite according to protocol B (2 h hypochlorite, 2 x 5 min ultrasound).

**Figure 2:**
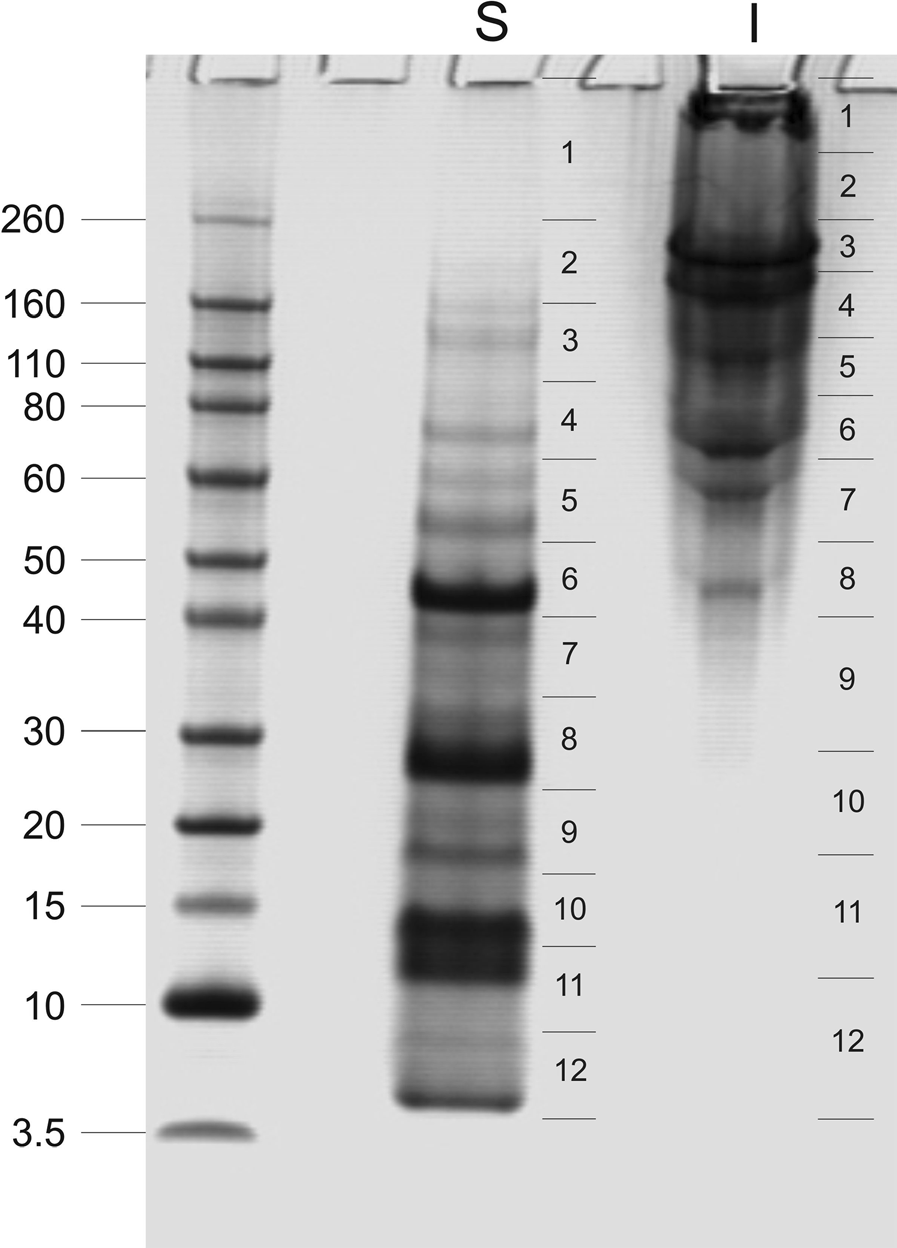
**PAGE comparison of acid-soluble and acid-insoluble matrix.** Molecular weight markers are indicated at the left. S, acid soluble matrix; I, acid-insoluble matrix. The sections for in-gel digestion are indicated at the right of each lane. With longer exposure times sections 1–8 of the acid-insoluble sample became a feature-less smear, while faint bands became apparent in sections 9–12.

The cleaning of invertebrate biominerals usually involves washing in sodium hypochlorite using different incubation lengths. This is supposed to destroy and remove organic material at the biomineral surface, while intra-crystalline organic matrix components are thought to be shielded from the destructive action of hypochlorite by the surrounding, densely packed, mineral. Because we wanted to study the effect of different sodium hypochlorite treatment length and the effect of ultrasonic treatment of shells during hypochlorite treatment on matrix composition, shells were either washed in hypochlorite solution for 2 h without (A) or with (B) short ultrasonic treatment, or for 24 h with short ultrasonic treatment (C). Comparison of the protein band pattern of the isolated matrices typically showed some minor, apparently predominantly quantitative rather than qualitative, differences (Figure 1A). However, PAGE comparison of matrices from different shells treated according to the same protocol showed comparable differences (Figure 1B). This suggests that not only experimental variables in the extraction protocol played a role, but possibly also individual biological factors, such as shell size, preservation and thickness of the outer calcitic shell layer, or environmental factors. The yields of organic matrix were between 2.2–5.3 mg/g of shell for the acid-soluble matrix, and between 2.1–4.6 mg/g for the acid-insoluble matrix (total of nine shells). The acid-insoluble matrix formed approximately half of the total organic matrix and the PAGE protein band patterns of soluble and insoluble matrices were very different (Figure 2). Therefore the proteomes of both fractions were analyzed separately. Several sets of data from different shells were evaluated together to establish a representative shell proteome. For A and B, four data sets (replicates) of matrices isolated from three different shells (8.8, 5.6, and 3.8 g of weight and 11.5, 9.1 and 4.1 g of weight, respectively) were analyzed. For C, two data sets were from a single large shell (8.6 g) and two data sets were from the pooled matrices of two small shells (2.9 and 1.5 g). Each data set was obtained from the analysis of tryptic peptides extracted from three gel lanes cut into 12 slices (Figure 2).

### Proteomic analysis of matrix fractions

**Figure 3:**
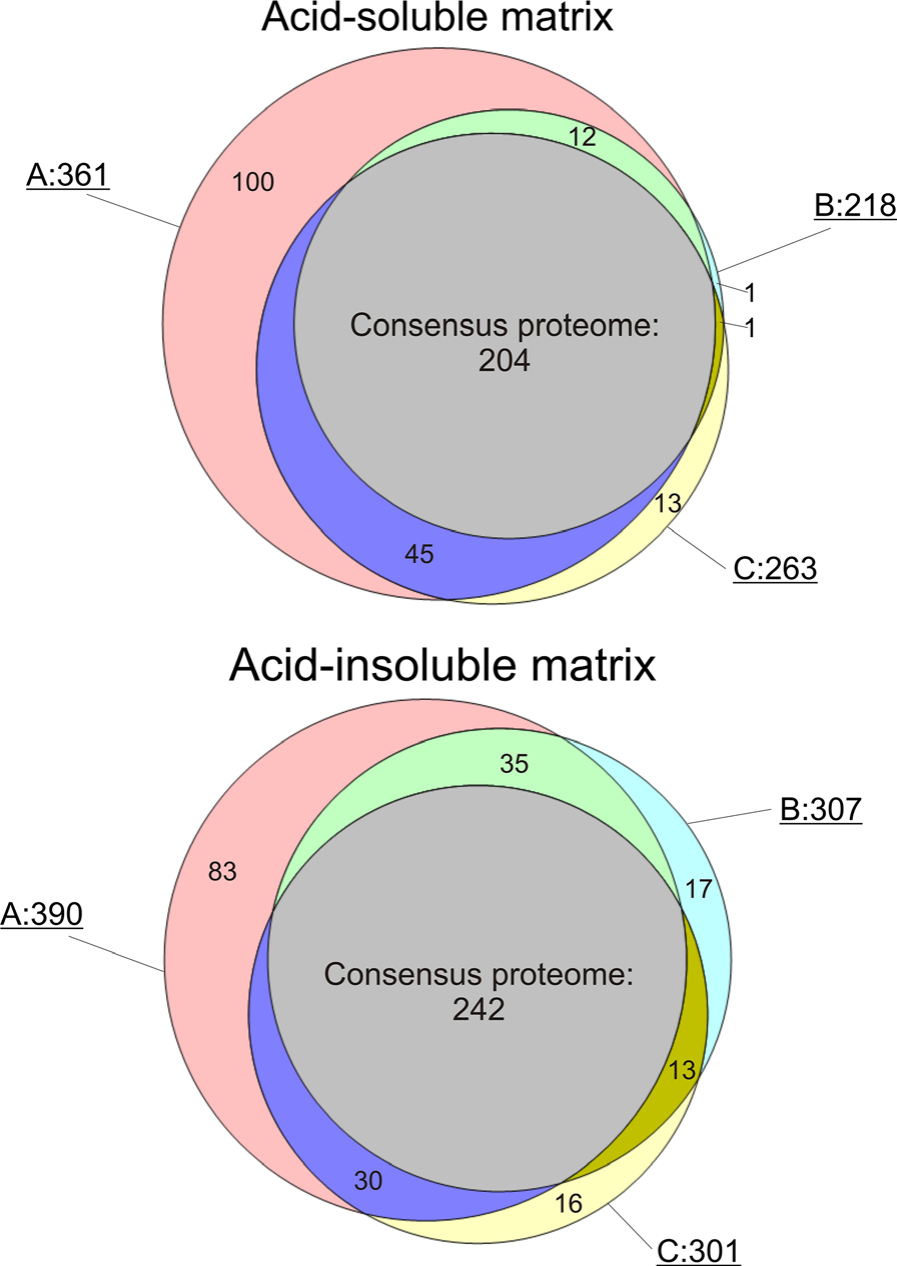
**Venn diagrams of protein identifications in different samples.** A, matrix isolated after sodium hypochlorite treatment of the shells for 2 h at room temperature. B, 2 h hypochlorite cleaning with 2 x 5 min ultrasound at the start of each hour. C, 24 h hypochlorite with 2 x 5 min ultrasound bursts as before and one after 24 h. The consensus proteome comprises all identifications occurring in all three types of samples. Venn diagrams were prepared using the Venn Diagram Plotter of http://omics.pnl.gov/software/VennDiagram Plotter.php.

Proteomic analysis of all fractions (Figure 3; Additional file 1 and Additional file 2) clearly showed the effect of ultrasound treatment. Approximately 28% of the proteins of the acid-soluble matrix and 21% of the acid-insoluble matrix of shells not treated with ultrasound during hypochlorite cleaning (A) were identified only in these matrices but not in B or C (Figure 3). Differences between B (2 h hypochlorite) and C (24 h hypochlorite) were less clear. Surprisingly the number of protein and peptide identifications in the soluble fraction of C was greater than that of B ( Additional file 1). Most of the proteins distinct between the two preparations were not unique but also occurred in A. This was difficult to explain, because all four replicates showed the same effect although they were prepared and analyzed at different times, sometimes on different mass spectrometers and often in sequence with replicates from other preparations. However, the qualitative differences between B and C were minor and focused almost exclusively on low abundance proteins. This may indicate that ultrasound treatment during cleaning with hypochlorite may have helped to solubilize and destroy proteins that stuck tenaciously to the biomineral surface. The length of hypochlorite treatment, however, apparently did not play a dominant role, at least after two hours of treatment. This aspect of hypochlorite treatment may become more important with nacreous shell layers, as our experience with *Haliotis laevigata* has shown that lengthy treatments start to degrade the matrix surrounding nacre plates, leading to a partial loss of the outermost nacre layers.

Altogether 569 proteins were identified in matrices obtained after different hypochlorite treatments. To obtain a representative, high-confidence, shell matrix proteome of *Lottia gigantea*, we assembled a consensus proteome comprising all database entries identified in all three types of samples (Figure 3). The consensus proteome of the acid-soluble fraction included 204 proteins and the consensus proteome of the acid-insoluble fraction contained 242 proteins. Given an overlap of 135, this summed up to a total of 311 *Lottia* database entries containing shell matrix protein sequences. However, these numbers should not be regarded as final because some database entries may eventually turn out to contain the sequence of more than one protein and some protein sequences may be divided among several database entries. Furthermore, the identifications not comprised in the consensus proteome are by no means to be considered as false positives but may be true shell matrix components. In most cases these were minor proteins and their absence or presence in different fractions may be due to experimental variability or the still limited dynamic range of mass spectrometers. Additional files 3, 4, 5, 6, 7, 8, 9, 10, 11, 12, 13, and 14 contain protein and peptide details, such as accession numbers of proteins sharing group-unique peptides, scores, masses, peptide sequences, and distribution in gel slices ( Additional files 3, 4, 5, 6, 7, 8, 9, 10, 11, 12, 13, and 14). Unlike Additional file 1 and Additional file 2 ( Additional file 1 and Additional file 2), Additional files 3, 4, 5, 6, 7, 8, 9, 10, 11, 12, 13, 14 contain data of all peptides and proteins identified within the set thresholds for MaxQuant searches (including identifications with one sequence-unique peptide), irrespective of whether they were accepted after manual inspection or not.

Both consensus proteomes contained intracellular proteins. In the soluble proteome these amounted to approximately 15% ( Additional file 1). The acid-insoluble fraction contained approximately 36% ( Additional file 2). Many of these proteins, such as the endoplasmatic reticulum and Golgi apparatus residents, may be by-products of secretion processes. Others may be releases into the extrapallial fluid by damaged or decaying cells of the epithelium lining the mantle cavity. Once in the extrapallial fluid, they have free access to the growing shell surface, may bind there, and may eventually be overgrown by further calcium carbonate deposition in shell growth periods. As true intra-crystalline components, although probably without any function, they may not be removed even by rigorous hypochlorite cleaning. Because the acid-insoluble consensus proteome contained more of these intracellular components, one may conclude that many of them were already structurally modified and aggregated before incorporation into the growing shell. Proteins of previously known intracellular location were also found in other invertebrate skeletal matrices analyzed in depth using similar proteomic technology [22-24]. However, it is rather unlikely that matrix components with a well-defined intracellular location have any function in the shell. However, specific functional shell matrix proteins may be found among the major matrix proteins and those with recognized or predicted extracellular location.

### Uncharacterized *Lottia* matrix proteins with unusual amino acid composition and short sequence repeats

The matrix of the *Lottia gigantea* shell contained many previously uncharacterized proteins (i.e. proteins without obvious sequence homology to known mollusc shell proteins) with unusual amino acid composition, short tandem repeats, and blocks of identical or similar amino acids (homorepeats). Often these characteristic primary sequence features are found in terminal regions of shell proteins that have been proposed to be structurally unstable, unfolded domains able to adopt a specific structure only upon binding to a ligand, such as a crystal surface [34]. This proposition was based on experiments with synthetic polypeptides confirming the intrinsically disordered conformation of such shell protein domains and the *in vitro* interaction with calcium carbonate [35-39]. However, most known features of such short linear motifs and homorepeats come from intracellular examples [40,41]. Apart from occurring predominantly in natively disordered structures, such motifs mediate protein-protein interactions with low affinity, which is usually compensated by frequent repetition of the motif. Examples of major (average emPAI ≥9) *Lottia* matrix proteins with peculiar primary sequence features are shown in Table 1. Many of these proteins either do not contain cysteines, which usually are disulfide-bonded in extracellular proteins and stabilize structured domains (except in the predicted signal peptide), or have cysteine-containing domains apart from the presumed intrinsically disordered sequence motifs. However, there are exceptions. Thus, in Lotgi1|173200, one of the most abundant proteins of the acid-soluble matrix ( Additional file 1), 30% of the sequence consists of Asn, Pro and Ser, but the sequence also contains 20 Cys, indicating a well-ordered structure stabilized by disulfide bonds. Database searches indicated some similarity to the *Pinctada fucata* shell mpn88 protein B7X6R9_PINFU (unpublished; submitted to EMBL by Nogawa et al., 2007). The proteins showed 27% sequence identity, but none of the 20 cysteines of Lotgi1|173200 was preserved in mpn88, which contains no cysteine at all in the predicted mature sequence. Therefore we prefer to accredit the similarity in database searches to regions of similar amino acid composition, but not to sequence homology. The same may be true for Lotgi1|231186 (Table 1).

**Table 1:** **Previously uncharacterized major*Lottia*shell matrix proteins with unusual primary sequence features**

**Accession**	**Feature**
Lotgi1|115147	14% P, 11% T, 6 repeats of ~30aa, starting with MITPE; pI: 4.7; 319aa
Lotgi1|142790	25% Q, 10% E, 17% P, 12% V, 10% N 10% L; 6 short repeats: k/qQQPxVELNKQQP; pI 5.2; 182aa
Lotgi1|142814	38% Q, 11% L, 10% P; 5 ~70aa repeats containing shorter repeat motifs like NQQQ and KQQQ; pI: 10.5; 322aa
Lotgi1|152688	20% G, 12% P; pI: 9.7; 137aa
**Lotgi1|158113**	11% P; Q-rich C-term (aa210–240); pI: 9.7; 258aa
Lotgi1|159331	26% E, 13% L,12% T; pI: 4; starting with aa156 8x SNLLQQPDa/tTQqLa/tTNeQQQ; (Figure 6**)**
Lotgi1|163637	17% D, 16% A; EFh, pI: 3.8; 643aa; 12 ca30aa repeats similar to AxVDNxxMADMIDTxQDxxEDAADNMADNIDTAQDAQ between aa32–453
Lotgi1|171084	13% S; frequent doublets (SS, QQ, TT, YY, NN); G/E block aa322–337; pI: 4.4; 357aa
Lotgi1|172698	23% Q, 13% N, 13% S; aa130–702: 31 x 14aa repeats similar to QSNQQFNxxQSNQQF; pI: 7.1; 1184aa
**Lotgi1|173200**	~10% of P, N and G; in aa107–170 10x GAMP/GSMP; pI: 9.6; 563aa
Lotgi1|174003	19% P in aa50–400 and 35% P in aa778–882; pI: 9.5; 882aa
**Lotgi1|227783**	aa17–126: 17% R + K, 12% P, 11% L; pI: 11; 126aa
Lotgi1|228385	16% R, 11% S; pI: 11.7; 160aa; R/H-rich from aa103–150
Lotgi1|231186	19% G, 12% P; aa433–481: 27% M; pI: 4.6; 481aa; R/H-rich C-term half
Lotgi1|231509	aa26–230: 18% P; pI: 4.2; 230aa; acidic blocks in N-term half
**Lotgi1|233397**	A/P-rich motif aa150–170; H-rich motif aa171–185; pI: 8.8; 219aa
**Lotgi1|233420**	31% D, 10% E; pI: 3.6; similar to aspein?
Lotgi1|234884	42% Q in aa281–630; G/L/A-rich region aa631–928; pI: 9.2; 928aa
**Lotgi1|235497**	aa120–247: 20% P, 16% A, 10% Q; pI: 9.7; 247aa
**Lotgi1|235610**	15% P, 15% T; pI:5.7; 557aa
**Lotgi1|235621**	aa171–270: 33% G, 25% T, 15% P, 14% Q; 16 x GGQPs/tT; pI: 5.4; 303aa
Lotgi1|235812	24% P, 18% Q, 10% N; pI: 8.9; 729aa; aa57–376: 17 repeats of 16aa, similar to NNxa/vQPPxxQxxYQPt/p
Lotgi1|236689	19% P, 10% A, 10% V, 10% R; pI: 10; 317aa
Lotgi1|236690	21% Q, 18% P; aa268–356: 4 xAQPGAYQQP(x)_2–4_ GAYxQQP repeats; pI: 8.4; 440aa
Lotgi1|236691	22% P, 13% Q, 10% A; Q-rich regions: ~aa61–160 and ~ aa721–990; P-rich: ~aa280–600 and ~780–970¸pI: 8.8; 1035aa
Lotgi1|238358	aa61–232: 32% D + E, 12% N; pI: 3.7; 323aa; (Figure 4**)**
**Lotgi1|238831**	13% A, 11% R, 11% L; K/R/A-rich C-terminus (aa185–219); pI. 10.3; 219aa
**Lotgi1|239170**	16% G, 12% M, 10% Q; G blocks in N-term half; pI: 9.9; 145aa
**Lotgi1|239174**	20% G, 18%M, 12%A, 10% L; pI: 11.2; 186aa; some similarity to shematrins
Lotgi1|239339	13% T, 12% S, 10% P; blocks of T from aa185–240; pI: 9.7; 609aa
**Lotgi1|239447**	22% G, 12% N; pI:9.5; 191aa; some similarity to GAAP_HALAI (Figure 5**)**
Lotgi1|77105	19% P, 15% S; 12% G; 9 x g/dSQPGIYP and 4 x imperfect; pI: 4.5; 173aa
Lotgi1|84059	23% N, 15% P, 15%T, 11% S; 7 repeats similar to TPxxxNNVNPGSETPxTxNNVNPGSE and 2 incomplete; pI: 3.8; 234aa

For complete lists of matrix proteins see Additional file 1 and Additional file 2. Accessions in bold belong to the 26 most abundant proteins with average emPAI >1000 ( Additional file 1 and Additional file 2).

**Figure 4:**
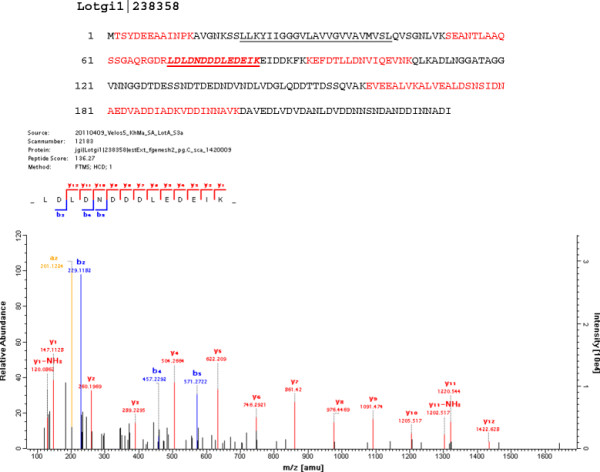
**The amino acid sequence of a very acidic protein, Lotgi1|238358.** Entry Lotgi1|238358 contains the sequence of a predicted transmembrane protein with a short intracellular domain (aa2–20), the predicted transmembrane segment (underlined) and a very acidic extracellular domain (theoretical pI 3.6) with Asp and Glu adding up to 30% of the amino acid composition. This protein was more abundant in the acid-insoluble than in the acid-soluble fraction. Sequences covered by MS/MS spectra a shown in red. The lower part shows the spectrum of one of the acidic, doubly charged peptides (shown in bold italics and underlined in the complete sequence) with m/z 831.3731, a mass error of 1.4 ppm and a PEP of 1.1E-12.

**Figure 5:**
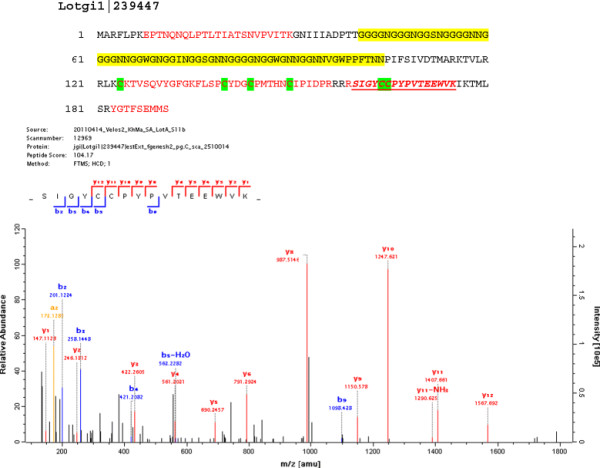
**The amino acid sequence of the Gly/Asn-rich protein in Lotgi1|239447.** This was one of the most abundant proteins in the acid-soluble matrix. The sequence contained a Gly/Asn-rich domain (aa41–105; shaded yellow) consisting of 55% Gly and 28% Asn. This is followed by a cysteine-containing domain (cysteines shaded green) that can be presumed to have a more rigid structure stabilized by disulfide bonds. The Gly/Asn-rich domain did not yield a peptide because of the lack of tryptic cleavage sites. However, it is framed by MS/MS-sequenced peptides. A very similar G/N-rich sequence region was found in the otherwise unrelated shell protein GAAP_HALAI, identified in *Haliotis asinina*[6] and in nacrein_like proteins [7,46]. Sequences covered by MS/MS are in red, the peptide giving rise to the spectrum is in bold italics and underlined. The doubly charged peptide with m/z 994.4501 and a deviation from the calculated value of 0.1 ppm had a PEP of 4.7E-13. Very typically, the most intense fragments, y8 and y10, were produced by preferential fragmentation N-terminal to Pro and in the +1 position of Pro.

**Figure 6:**
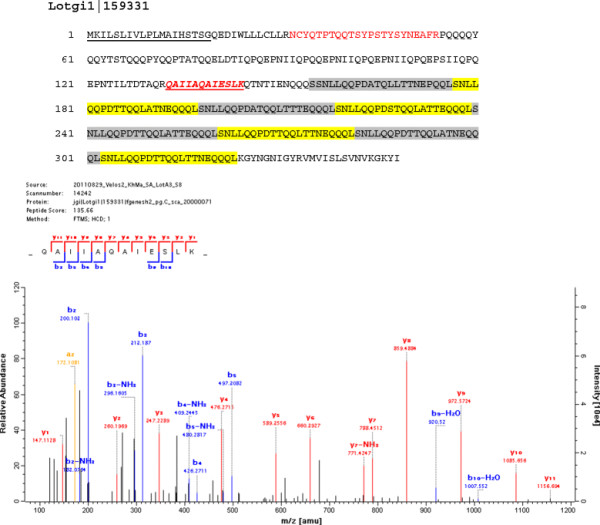
**The amino acid sequence of Lotgi1|159331, an acidic Gln-rich protein with multiple sequence repeats.** The predicted secretion signal sequence (aa1–19) is underlined. Sequences covered by MS/MS are in red, the peptide giving rise to the spectrum below is in bold italics and underlined. The theoretical pI for this sequence is 4.0, and the amino acid composition includes 27% Gln, 13% leu and 12% Thr. Eight 21aa-long Gln-rich sequence repeats are alternately shaded grey and yellow. No peptides from the repeat region were obtained because of the lack of tryptic cleavage sites. The doubly charged peptide with m/z 642.80 and a mass deviation of 0.6 ppm had a PEP of 6.2E-09.

Selected sequences and spectra of this group are shown in Figures 4,5,6. Several of these proteins shared their sequence features with recently discovered shell proteins. Thus, the very acidic protein in Lotgi1|233420, which is one of the most abundant proteins in *Lottia* shell matrix ( Additional file 1 and Additional file 2), shows 36% sequence identity to aspein [42], but this is based almost exclusively on alignment of aspartic acids. Extended Asp-rich sequences also occur in other shell proteins, such as MSP-1 [43] and asprich [44]. A very similar acidic domain was also contained in the C-terminal third of Lotgi1|239188, while the N-terminal domain was similar to nacrein (Table 2). Glycine-rich proteins may be relatives of shematrins [45]. However, in the absence of significant sequence similarity in non-repetitive sequence regions a possible homology is difficult to prove. The *Lottia gigantea* shell matrix also contained several proteins with sequence similarity to previously identified mollusc shell proteins (Table 2) discussed below.

**Table 2:** ***Lottia*matrix proteins with possible sequence homologs in other shells**

**Accession**	**Suggested homolog**^**1**^	**Organism**	**Reference**	**Sequence identity**^**2**^	**Alignment**
**Lotgi1|140660**	BMSP (fragment) Pif (fragment)	*M. galloprovincialis* *Pinctada fucata*	[47,48]	44% (5.0E–30) 27% (1.3E–6)	Additional file 16
**Lotgi1|173138**	BMSP (fragment) Pif (fragment)	*M. galloprovincialis* *Pinctada fucata*	[47,48]	37% (1.6E–33) 27% (3.2E–13)	Additional file 16
**Lotgi1|238526**	BMSP 100	*Mytilus galloprovincialis*	[48]	21% (4.0E–7)	Additional file 16
Lotgi1|133595	dermatopontin	*Biomphalaria glabrata*	[49]	31% (6.6E–17)	Figure 7
Lotgi1|233583	ependymin-related protein	*Haliotis asinina*	[6]	27% (6.5E–9)	Additional file 15: Figure SA
Lotgi1|235548 aa170–540	gigasin-2	*Crassostrea gigas*	[9]	26% (8.6E–4)	Additional file 15: Figure SB
Lotgi1|132911	Kunitz-type protease inhibitor KCP_HALAI	*Haliotis asinina*	[6]	56% (3.6E–18)	
Lotgi1|233461	nacrein B4/B3/A1/B2	*Pinctada margaritifera*	[7]	36–38% (1.6E–9 – 5.2E-6)	
**Lotgi1|238082**	nacrein-like protein	*Mytilus californianus*	[10]	25% (4.1E–13)	Additional file 15: Figure SC
Lotgi1|239188 (aa1–420)	nacrein B2/B3/A1/B4; aa421–633 very acidic, with similarity to such proteins as aspein	*Mytilus californianus*	[10]	27–33% (4.1E-6 – 3.3E-5)	
Lotgi1|229175 (aa1–156)	perlucin_like	*Mytilus galloprovincialis*	[10]	26% (1.3E-4)	Figure 8
Lotgi1|235529 (aa1–165)	perlucin_like	*Mytilus galloprovincialis*	[10]	31% (1.0E-4)	Figure 8
**Lotgi1|174065**	perlustrin	*Haliotis laevigata*	[50]	33% (0.076)	Figure 9
**Lotgi1|238970**	perlustrin	*Haliotis laevigata*	[50]	39% (1.1E-7)	Figure 9
Lotgi1|143247	perlwapin	*Haliotis laevigata*	[51]	31% (0.003)	
Lotgi1|201804	perlwapin	*Haliotis asinina*	[6]	35% (1.2E-5)	
Lotgi1|239125	perlwapin	*Haliotis laevigata*	[51]	40% (4.3E-9)	
Lotgi1|228264	Pif (fragment) BMSP (fragment)	*Pinctada fucata* *M. galloprovincialis*	[47,48]	28% (5.8E-5) 29% (1.1E-11)	Additional file 17
Lotgi1|232022	Pif BMSP	*Pinctada fucata/* *Mytilus galloprovincialis*	[47,48]	24% (3.3E-15) 32% (5.0E-12)	Additional file 17
Lotgi1|239574 (~aa300–650)	BMSP Pif	*Mytilus galloprovincialis/* *Pinctada fucata*	[47,48]	22% (5.9E-9) 28% (4.6E-4)	Additional file 17
Lotgi1|237510	P86860 Pif	*Mytilus californianus* *Pinctada fucata*	[10,48]	28% (2.0E-9) 44% (1.0E-11)	
Lotgi1|166196 (aa1–400)	tyrosinase	*Pinctada fucata*	[52,53]	35% (5.7E-5)	Additional file 15: Figure SD
**Lotgi1|231009**	UP2	*Haliotis asinina*	[6]	28% (2.9)	Additional file 15: Figure SE

For complete lists of matrix proteins see Additional file 1 and Additional file 2. ^1^, identified in database searches against complete databases (UniProt Knowledgebase, NCBI non-redundant protein sequences) the suggested homolog was usually not the best match, but the best mollusc shell match. ^2^, sequence identity in regions of sequence similarity identified by database searches; E values for the FASTA results are shown in brackets. Accessions in bold belong to the 26 most abundant proteins with average emPAI > 1000 ( Additional file 1 and Additional file 2).

### Proteins with possible homologs in other shells

#### Dermatopontin, ependymin-like and gigasin-2-like proteins

**Figure 7:**
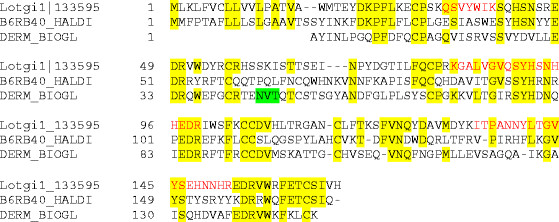
**Comparison of Lotgi1|133595 to dermatopontin.** The sequence of Lotgi1|133595 is compared to the sequence of *Biomphalaria glabrata* dermatopontin [49] and to the unpublished sequence of *Haliotis discus* dermatopontin submitted to EMBL by H.-S. Kang, M. De Zoysa and J. Lee. Peptides sequenced by MS/MS are shown in red. The N-glycosylation site of *B. glabrata* dermatopontin is shaded green. The *Biomphalaria* sequence is the sequence of the mature protein determined by Edman degradation and therefore lacks a secretion signal peptide.

The first mollusc shell dermatopontin was isolated from the freshwater snail *Biomphalaria glabrata* shell matrix [49]. Since then several molluscan dermatopontin-encoding genes have been identified and some of them were transcribed in mantle cells, implying the shell matrix as final destination [17,54,55]. A protein very similar to dermatopontin, Lotgi1|133595 (Figure 7), was identified at moderate abundance in the acid-insoluble matrix consensus proteome and in the soluble fraction of A and C ( Additional file 1 and Additional file 2). The function of this protein remains unknown at present [55].

A protein similar to the ependymin-related proteins recently discovered in *Haliotis asinina* shells [6] was found in Lotgi1|233583, a minor protein of the acid-insoluble consensus proteome ( Additional file 1 and Additional file 2). It was also similar to an unpublished *Haliotis discus* protein submitted to databases by Kang et al. (2006) under the name X-box binding protein with the accession number B6RB39 ( Additional file 15 Figure SA). The function of ependymin and related proteins is unknown at present.

Entry Lotgi1|235548 contained a protein sequence partially (~aa170-540) similar to the recently discovered *Crassostrea gigas* shell protein gigasin-2 (*Cgigas*-IMSP-2) [9] and the related proteins EGF-like domain containing protein-1 and −2 from *Pinctada maxima* [Jackson et al., 2009] ( Additional file 15 Figure SB). Lotgi1|235548 was a minor protein in both, acid-soluble and acid-insoluble, consensus proteomes ( Additional file 1 and Additional file 2).

#### Nacrein-like proteins

One of the most important enzymes in biomineralization events is carbonic anhydrase, which catalyzes the formation of hydrogen carbonate from CO_2_ and water. The first carbonic anhydrase isolated from a mollusc shell and characterized at the molecular level was nacrein [46]. This protein, which was isolated from the nacreous layer of *Pinctada fucata* shells, contained two carbonic anhydrase domains separated by a Gly-X-Asn repeat domain. The same protein was also identified in the prismatic layer [56]. Since then nacrein-like proteins or nacrein-encoding genes have been identified in several other molluscs [4,7,10,57,58].

The *Lottia* shell matrix contained three entries that showed some similarity to nacreins (Table 2). Of these Lotgi1|238082 belonged to the most abundant proteins in the shell matrix ( Additional file 1 and Additional file 2) and its sequence was 25% identical to that of *Mytilus californianus* nacrein-like protein [10] ( Additional file 15 Figure SC). It is comprised of a single α-CA_2 domain preceded by a predicted secretion signal sequence. The peak of protein distribution along gels was in slice 6. This was in agreement with the predicted protein mass (44.7 kDa) and coincided with a major band in the PAGE pattern (Figure 2). A less abundant but still major protein was Lotgi1|239188. The sequence contained a predicted secretion signal sequence and a single α-CA_2 domain (aa87–411). This was followed by a region containing 26% Asp, 23% Gly, 22% Arg and 13% Asn that aligned with 32–37% identity to the GN- and GXN-rich domains of nacreins. The CA domain was 33% identical to the sequence of an unpublished *Haliotis tuberculata* protein (accession G0YY03 of UniProt, submitted as carbonic anhydrase by LeRoy et al., 2011) and only 23% to the sequence of *Mytilus californianus* nacrein-like protein [10]. Lotgi1|233461 contained neither a secretion signal sequence nor a predicted CA domain, but showed 36–38% sequence identity to nacrein regions preceding and comprising the GN- and GXN-rich domains. Therefore its relation to nacrein remains inconclusive. In addition to nacrein-like proteins the *Lottia* shell matrix contained two other predicted carbonic anhydrases apparently completely unrelated to nacreins (see below and Table 3).

**Table 3:** Other proteins with a possible or established link to biomineralization

**Accession**	**Protein**	**Comment**
**Lotgi1|230492**	Similar to calcineurin	30% identity in a 120aa overlap (Fasta E value: 0.37) with *Pinctada fucata* calcineurin (C1ITK0_PINFU) [59,60]; EFh;
Lotgi1|205401	Carbonic anhydrase	Minor protein; possibly intracellular
Lotgi1|66515	Carbonic anhydrase	Major protein in acid-soluble shell proteoime; possibly intracellular
Lotgi1|159694	Chitin-binding	Minor protein, 4 chitin-binding peritrophin A domains and 4–6 SRCR (scavenger receptor-related) domains
Lotgi1|160173	Chitin-binding	Major protein, secreted; 2–3 chitin-binding peritrophin A domains
Lotgi1|231395	Chitin-binding	Sequence contains predicted secretion signal sequence followed by two chitin-binding peritrophin A domains
Lotgi1|226726	Chitin-binding	Major protein in acid-soluble, minor in acid-insoluble consensus proteome; chitin-binding_3 domain
Lotgi1|231869	Chitin-binding	Major protein in acid soluble proteome; 10 chitin-binding perotrophin A domains organized in two blocks separated by four Pro-rich extensin-like motifs (aa470–600; 29% Pro, 16% Thr, 12% Gln, 12% Asn)
Lotgi1|232880	Chitin-binding/chitinase	Major protein in acid-insoluble proteome; several SEA domains; chitin-binding peritrophin domain (aa2140–2200)with some similarity to chitinases
Lotgi1|234405	Chitin-binding	Major protein in acid soluble proteome; four chitin-binding peritrophin A domains preceded by a predictedsecretion signal sequence
Lotgi1|238400	Chitin-binding	Major protein in acid-insoluble proteome; predicted secretion signal sequence, VWA domain and Chitin-binding peritrophin A domain
Lotgi1|209107	Chitinase	Lysosomal; chitin degradation; major protein
Lotgi1|181237	Chitin deacetylase	Minor secreted protein
Lotgi1|156599	FAM20C/DMP4	Extracellular matrix protein; minor
Lotgi1|109908 Lotgi1|176394	Osteonectin/SPARC/BM-40	Overlapping fragments; extracellular matrix protein; major in acid-soluble matrix, minor in acid-insoluble matrix; Additional file 15: Figure SF

Accessions in bold belong to the 26 most abundant proteins with average emPAI > 1000 ( Additional file 1 and Additional file 2). For complete lists of matrix proteins see Additional file 1 and Additional file 2.

#### Proteins with CLECT, IGFBP and WAP domains

**Figure 8:**
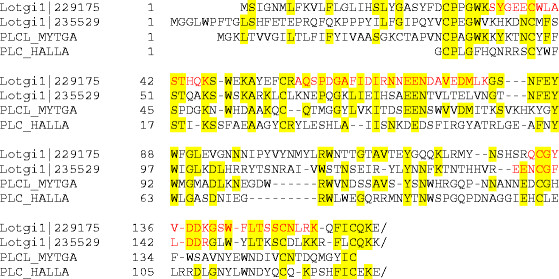
**Sequence comparison of perlucin-like proteins.** Peptides sequenced by MS/MS are shown in red. The sequence of PLCL_MYTGA is from [15] (P86854), PLC_HALLA is from [62] (P82596). This latter sequence had been determined by Edman degradation with the isolated mature protein. Therefore there is no secretion signal sequence as in the other sequences.

The C-type lectin perlucin was first identified and isolated as a major protein of the nacreous layer of *Haliotis laevigata* shells [61,62]. C-type lectin-like (CLECT) domains were detected in several *Lottia* matrix proteins ( Additional file 1 and Additional file 2), two of which were reasonably similar to perlucin to be considered as homologs (Lotgi1|229175 and Lotg1|235529; Figure 8). However, in both entries the perlucin-like domain was joined to a ZP (zona_pellucida)_2 domain. This resulted in a predicted mass of approximately 57,000 for the presumed products. The peptides of both domains were found predominantly in gel slices four and five (Figure 2). This was in good agreement with the predicted M_r_ of the complete protein, indicating that the domains occurred in the same protein. Therefore it remains questionable whether the *Lottia* shell matrix contained a true perlucin homolog. While Lotgi1|229175 was an abundant protein in the consensus proteomes of acid-soluble and acid-insoluble fractions, Lotgi1|235529 was a minor protein only identified in the acid-soluble fraction of preparation A ( Additional file 1 and Additional file 2). Lotgi1|235549 was a minor consensus proteome component with a chain of 11 predicted CLECT domains preceded by two predicted EGF and one ZP_2 domains. Finally, in the predicted minor transmembrane protein Lotgi1|156525 a single CLECT domain with limited similarity to mollusc perlucins was joined by several CUB; Sushi and EGF domains. Perlucin was recently also detected in the shell of a *Mytilus* species [10].

**Figure 9:**
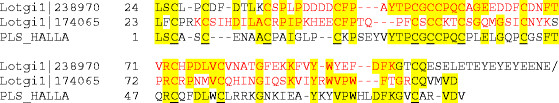
**Sequence comparison of perlustrin-like proteins.** Peptides sequenced by MS/MS are shown in red. Unlike the *Lottia* proteins, *Haliotis laevigata* perlustrin has no secretion signal sequence because the mature protein had been sequenced by Edman degradation [50] (P82595).

Compared to perlucin, the EGF- and insulin-binding protein perlustrin was a minor component of the *Haliotis laevigata* shell nacre matrix [50,61]. However, its predicted homolog (Figure 9) Lotgi|174065 was one of the most abundant proteins in the *Lottia* matrix ( Additional file 1 and Additional file 2). A second perlustrin-like protein (Figure 9), Lotgi1|238970, was less abundant, but still a major protein. To our knowledge no perlustrin-like protein has been found in shells other than *Haliotis laevigata* and *Lottia gigantea*.

**Figure 10:**
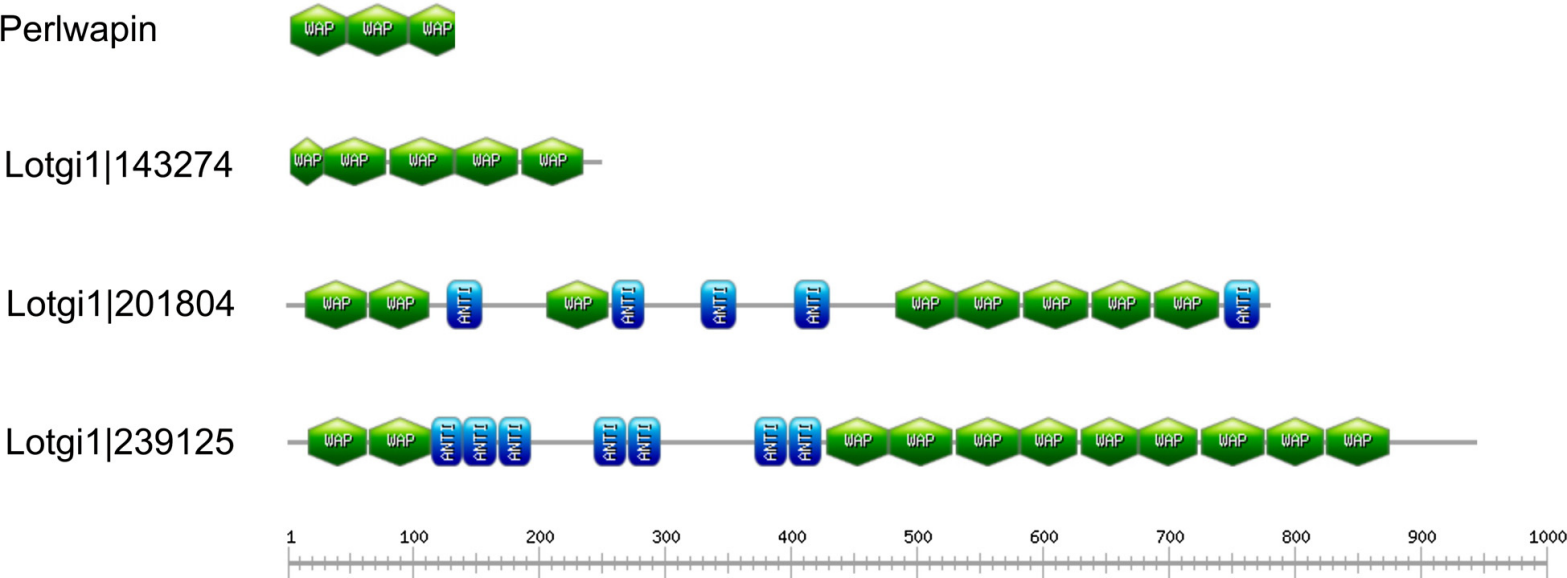
**Domain organization of WAP-containing proteins of the shell matrix.** WAP (whey acidic protein) domains are shown in green, antistasin-like protease inhibitor domains are shown in blue. Lotgi1|143274 starts with a partial WAP domain. Perlwapin is the *Haliotis laevigata* protein [51]. Domain borders were determined with Prosite (http://prosite.expasy.org/), the drawing was prepared with the help of Prosite MyDomains (http://prosite.expasy.org/cgi-bin/prosite/mydomains/).

Another major protein of *Haliotis laevigata* nacre matrix is perlwapin [51], which derives its name from three whey acidic protein (WAP), also called four-disulfide core domains. WAP domains are widespread among vertebrates and invertebrates [63] and proteins very similar to *Haliotis laevigata* perlwapin were recently identified in *Haliotis asinia*[6] and *Mytilus galloprovincialis*[10]. The *Lottia* shell matrix contained three proteins with WAP domains (Figure 10). Lotgi1|143247 and Lotgi1|201804 were minor proteins of the acid-soluble consensus proteome, while Lotgi|239125 was a major constituent of both, acid-soluble and acid-insoluble, consensus proteomes ( Additional file 1 and Additional file 2). Lotgi1|143247 contained four complete WAP domains and what appeared to be a partial WAP domain at the N-terminus with four cysteines instead of the canonical six-cysteine pattern. Lotgi1|201804 contained eight WAP domains (Figure 10) separated into three groups by predicted antistasin-like protease inhibitor domains. The peptides that identified this protein were almost all derived from gel slices 3 and 4 in agreement with the calculated mass of the intact protein of approximately 85,000. Lotgi1|239125 contained two WAP domains at the N-terminus and an array of nine WAP domains in the C-terminal half, the two groups being separated by proteinase inhibitor/antistasin domains (Figure 10). As is usual with very abundant proteins the peptides were derived from several gel slices, but the distribution peaked in slice 3 and neighboring slices. This was compatible with a calculated mass of approximately 103,000 and indicated that the database entry comprised a single protein.

#### Pif- and BMSP-like proteins

Several identified *Lottia* proteins showed similarity to the recently described acidic P*inctada fucata* nacre matrix protein Pif [47] and its *Mytilus galloprovincialis* homolog BMSP [48] (Table 2; Additional file 16 and Additional file 17). Pif is synthesized as a large precursor cleaved into two products, Pif97 and Pif80. Pif97 contains a von Willebrand type A (VWA) domain and a chitin-binding peritrophin A domain. Pif80, which does not contain any known domain, induces the formation of aragonite. Similarly, BMSP is cleaved into BMSP120, which contains four VWA domains and a chitin-binding domain, and BMSP100, the calcium carbonate-binding protein. The sequence of Pif80 and BMSP100 were described as completely different [48]. A Pif-related protein was also identified in *P. margaritifera*[7].

Lotgi1|140660 and Lotgi1|173138 were highly abundant in the acid-insoluble matrix and moderately abundant in the acid-soluble matrix ( Additional file 1 and Additional file 2). The sequence of Lotgi1|140660 contained two predicted VWA domains, but no signal peptide. Lotgi1|173138 contained no VWA domain, no signal sequence, but a chitin-binding domain. As often observed with major proteins, the peptides were detected in all slices of the gel. However, there was an unequivocal tendency towards slices from the high molecular weight region (see, for instance, Additional file 13) indicating that both entries possibly represented cleavage products of a larger protein. Lotgi1|238526 was one of the most abundant proteins in the acid-insoluble *Lottia* shell proteome and a much less abundant, but still major, protein of the acid-soluble matrix ( Additional file 1 and Additional file 2). The sequence showed a low similarity to the aragonite-binding part of BMSP. The overall sequence identity was 21%, but in the C-terminal ~100 amino acid-long sequence it rose to 40% ( Additional file 16). Because these three entries occurred at the same abundance level and were more similar to BMSP than to Pif (Table 2), we believe that they belong together and may represent fragments of a possible *Lottia* BMSP homolog.

Lotgi1|228264 was part of both consensus proteomes but was much less abundant than the presumed BMSP fragments described before ( Additional file 1 and Additional file 2). This protein contained a signal sequence, a VWA domain, and a chitin-binding domain. The difference in abundance to the previously described fragments indicated that this protein was a possible Pif homolog rather than a possible BMSP homolog, although it was as similar to BMSP as to Pif in database searches. Lotgi1|232022 was a minor protein of the acid-insoluble consensus proteome and also occurred in fractions A and C of the acid-soluble matrix. It contained a predicted VWA domain and a chitin-binding domain, but no signal sequence ( Additional file 1 and Additional file 2). The sequence aligned to Pif in the same region as Lotgi1|228264 and may be a minor Pif-related protein of the shell matrix ( Additional file 17). Lotgi1|239574 was a major protein of both consensus proteomes. The sequence contained a secretion signal and a predicted chitin-binding domain. The chitin-binding domain was preceded by a Thr-rich motif (aa300–370; 59% Thr). This arrangement of chitin-binding domain and Thr-rich motif was very similar to Lotgi1|228264 and Lotgi1|232022 ( Additional file 17). Our results indicate that the *Lottia* shell matrix may contain at least three Pif-related proteins occurring at different abundances. We did not identify the aragonite-binding part of any of these possible Pif homologs. However, the sequence of this part of Pif does not contain a known domain structure and may be poorly conserved between species [Suzuki et al., 2009; 2011], probably rendering identification by database searches difficult.

Both Prosite and InterProScan predict a second chitin-binding domain immediately after the published chitin-binding domain of *Mytilus galloprovincialis* BMSP and *Pinctada fucata* Pif. This domain was also predicted in all of the *Lottia* BMSP- and Pif-related proteins described above. In contrast to the regular invertebrate chitin-binding domain with six cysteines there was a cysteine doublet intercalated between regular Cys3 and Cys4 of the normal pattern ( Additional file 16 and Additional file 17). This was reminiscent of cysteine patterns in plant chitin-binding domains, where a cysteine doublet is inserted between Cys2 and Cys3 [64,65]. Therefore it is not clear whether these sequence motifs are really chitin-binding domains and consequently they were not considered in the respective figures ( Additional file 16 and Additional file 17).

Lotgi1|237510 was a major protein in the acid-soluble and a less abundant protein in the acid-insoluble consensus proteome ( Additional file 1 and Additional file 2). This protein showed similarity to the recently described chitin-binding protein P86860 of different *Mytilus* species [10] (Table 2) but part of it (aa1–100) was also predicted to be similar to Pif in database searches.

#### Tyrosinase-like proteins

Lotgi1|166196 encoded a minor protein of the acid-insoluble consensus proteome that was predicted to contain a secretion signal sequence and a tyrosinase domain. Database searches indicated similarity of ~ aa1–400 of this protein to several molluscan tyrosinases previously shown to occur in shells [7,52], or to be synthesized by mantle cells [17,53] indicating the shell as destination ( Additional file 15 Figure SD). In addition the sequence was very similar to other molluscan tyrosinase database entries, the known localization of which are either not in shells or was not reported. The C-terminal half of Lotgi1|166196 contained nine repeats of the type GPPVNP (aa393–462). Tyrosinase was suggested to function in periostracum formation of *Pinctada fucata*[53]. A second, unrelated, putative tyrosinase was found in Lotgi1|234481, but this protein was of low abundance, did not contain a secretion signal sequence, and was only identified in acid-insoluble fractions A and C.

#### Miscellaneous proteins

Lotgi1|171918 contained a sequence with high similarity to the protease inhibitor antistasin. However, the sequence was also similar to aa660–950 of the *Haliotis rufescens* shell protein lustrin A [66]. Two other entries, Lotgi1|231010 and Lotgi1|237013 matched to aa980–1420 of lustrin A in database searches. However, these matches were not convincing and were probably due to similarities in amino acid composition. Most importantly, the typical cysteine pattern of the lustrin A cysteine-rich repeats was not conserved in all of these *Lottia* sequences.

Lotgi1|132911 contained a fragment of a Kunitz-type protease inhibitor sequence similar to a recently published *Haliotis asinina* shell protein (Table 2) [6]. Lotgi1|231009, one of the most abundant proteins in the acid-soluble shell matrix, showed some similarity to the *Haliotis asinina* protein UP2 (Uncharacterized Protein 2; Table 2; Additional file 15 Figure SE) [6].

#### Other proteins of possible interest in biomineralization

Lotgi1|230492 contained a sequence with 30% identity in a ~120aa overlap with *Pinctada fucata* calcineurin B [59] and a predicted secretion signal sequence. This protein was implicated in shell regeneration processes recently [60] and was a major component of the acid-soluble proteome ( Additional file 1).

Chitin is a major non-protein component of mollusc shells [67-69] and the inhibition of chitin synthase has dramatic effects on the structure of newly formed larval shell [70]. This water-insoluble polysaccharide was suggested from structural studies to constitute a framework binding silk-like and acidic proteins [71]. Apart from proteins similar to Pif or BMSP described above, we have retrieved several proteins with predicted chitin-binding domains but without significant similarity to known shell matrix proteins in database searches (Table 3). In addition we identified a few putative chitin-degrading enzymes that could play a role in shell construction or repair by modifying the chitin framework (Table 3).

In addition to nacrein-like carbonic anhydrases we identified two putative carbonic anhydrases without obvious similarity to nacrein in sequence similarity searches (Table 3). Lotgi1|205401 was a minor carbonic anhydrase with approximately 40% sequence identity to a *Pinctada fucata* enzyme recently submitted to databases by H. Miyamoto (E5RQ31_PINFU). Lotgi1|66515 contained another predicted carbonic anhydrase, which was a moderately abundant protein in the acid-soluble matrix proteome ( Additional file 1). The lack of a secretion signal sequence indicated an intracellular origin of this protein. Possible roles for these two carbonic anhydrases in the mineralization process remain unclear at present.

FAM20C, also known as dentin matrix protein 4, was first detected in mouse dentin matrix [72] and may play a regulatory role in osteogenesis and odontogenesis of the mouse. However, similar proteins have also been detected in invertebrates. The sequence in Lotgi1|156599 was 41% identical to the mouse sequence and more than 60% to an uncharacterized putative *Daphnia pulex* protein (E9GAB5_DAPPU). The regulatory properties of this protein in vertebrates may implicate this minor shell protein in *Lottia* shell production.

Osteonectin was first isolated from bone matrix [73] but was soon recognized to occur in many other tissues as well. Sequence comparisons established identity of osteonectin with the basement membrane protein BM-40 [74] and a serum albumin-binding protein secreted by endothelial cells in culture, later called SPARC [75]. Since then many functions have been proposed for this protein, including a regulatory role in some biomineralization events in mammals [76]. *Lottia* osteonectin was a major protein in the acid-soluble shell matrix proteome and a minor one in the acid-insoluble fraction ( Additional file 1 and Additional file 2). Lotgi1|109908 contained the C-terminus of the protein, the N-terminus was identified in the first 135 amino acids of Lotgi1|176394 ( Additional file 15 Figure SF). Related proteins were reported from *Haliotis discus* and *Pinctada fucata* (unpublished, UniprotKB/TrEMBL accessions F2Z9K1_PINFU and F2Z9K2_HALDI, submitted by H. Miyamoto and F. Asada) and the sequences were included in the sequence alignment ( Additional file 15 Figure SF) together with the human sequence [77]. A possible role in molluscan biomineralization is unknown at present.

## Conclusions

The *Lottia gigantea* shell matrix turned out to contain a rather diverse set of proteins, comparable in complexity to the few other invertebrate shell matrix proteomes analyzed in-depth at present [21-23]. Among the 569 proteins identified by high-resolution mass spectrometry-based proteomics were at least 23 with a clear similarity to previously identified bivalve or gastropod shell matrix proteins. Others showed characteristics shared with previously known shell proteins, such as long stretches of acidic amino acids, of glycine, proline, or other amino acids. This made unequivocal recognition of homology difficult, if not impossible. However, such features as similar amino acid composition or preservation of domain structures may at least suggest functional equivalence. In addition we have identified many previously unknown proteins that may eventually turn out to play an important role as framework components or in regulation of matrix assembly and crystallization of the mineral. Despite the long list of identified proteins we do not expect to have identified all *Lottia* shell matrix proteins. Some may have been missed because of a lack of specific cleavage sites while others may not be represented adequately in the present draft of the database. Other known proteins may have been identified but were not recognized because of a low preservation of amino acid sequence. Nevertheless, we hope that this set of data, the most comprehensive list of mollusc shell matrix proteins available at present, may provide a starting point for the functional characterization of these proteins by researchers interested in biomineralization processes.

## Abbreviations

Aa = Amino acid; BMSP = Blue Mussel Shell Protein; CA = Carbonic anhydrase; CLECT = C-type lectin; IGFBP = Insulin-like growth factor-binding protein; emPAI = Exponentially modified protein abundance index; FDR = False discovery rate; HCD = Higher-energy collision-induced decomposition; PAGE = Polyacrylamide gel electrophoresis; PEP = Posterior error probability; VWA = Von Willebrand type A; WAP = Whey acidic protein.

## Competing interests

The authors declare that they have no competing interests.

## Authors’ contributions

KM conceived the study, performed sample preparation and data acquisition. EEG collected and mechanically cleaned *Lottia* shells and helped with database search and annotation. MM supplied methodological expertise. All authors took part in the design of the study and were critically involved in manuscript drafting. All authors read and approved the final manuscript.

## Supplementary Material

Additional file 1 ***Lottia gigantea*acid-soluble matrix proteins.** Doc-file containing a list of all accepted protein identifications, their distribution in matrices obtained after different sodium hypochlorite treatments, the number of unique peptides, emPAI values and previously known or predicted subcellular occurrence. Click here for file

Additional file 2 ***Lottia gigantea*acid-insoluble matrix proteins.** Doc-file containing a list of all accepted protein identifications, their distribution in matrices obtained after different sodium hypochlorite treatments, the number of unique peptides, emPAI values and previously known or predicted subcellular occurrence. Click here for file

Additional file 3 **Proteins identified in acid-soluble matrix A.** Xls-file containing MaxQuant output data such as Lotgi1 entries grouped together because of sequence identity, number of sequence-unique and non-unique peptides, sequence coverage, protein length and molecular weight, PEP values and distribution among gel slices. Click here for file

Additional file 4 **Peptides identified in acid-soluble matrix A.** Xls-file containing MaxQuant output data concerning peptides, such as peptide sequence, mass, score, PEP and distribution among gel slices. Click here for file

Additional file 5 **Proteins identified in acid-soluble matrix B.** Xls-file containing MaxQuant output data such as Lotgi1 entries grouped together because of sequence identity, number of sequence-unique and non-unique peptides, sequence coverage, protein length and molecular weight, PEP values and distribution among gel slices. Click here for file

Additional file 6 **Peptides identified in acid-soluble matrix B.** Xls-file containing MaxQuant output data concerning peptides, such as peptide sequence, mass, score, PEP and distribution among gel slices. Click here for file

Additional file 7 **Proteins identified in acid-soluble matrix C.** Xls-file containing MaxQuant output data such as Lotgi1 entries grouped together because of sequence identity, number of sequence-unique and non-unique peptides, sequence coverage, protein length and molecular weight, PEP values and gel slice origin of proteins. Click here for file

Additional file 8 **Peptides identified in acid-soluble matrix C.** Xls-file containing MaxQuant output data concerning peptides, such as peptide sequence, mass, score, PEP and distribution in gel slices. Click here for file

Additional file 9 **Proteins identified in acid-insoluble matrix A.** Xls-file containing MaxQuant output data such as Lotgi1 entries grouped together because of sequence identity, number of sequence-unique and non-unique peptides, sequence coverage, protein length and molecular weight, PEP values and gel slice origin of proteins. Click here for file

Additional file 10 **Peptides identified in acid-insoluble matrix A.** Xls-file containing MaxQuant output data concerning peptides, such as peptide sequence, mass, score, PEP and distribution of peptides among gel slices. Click here for file

Additional file 11 **Proteins identified in acid-insoluble matrix B.** Xls-file containing MaxQuant output data such as Lotgi1 entries grouped together because of sequence identity, number of sequence-unique and non-unique peptides, sequence coverage, protein length and molecular weight, PEP values and gel slices yielding peptides of the respective proteins. Click here for file

Additional file 12 **Peptides identified in acid-insoluble matrix B.** Xls-file containing MaxQuant output data concerning peptides, such as peptide sequence, mass, score, PEP and distribution of peptides among gel slices. Click here for file

Additional file 13 **Proteins identified in acid-insoluble matrix C.** Xls-file containing MaxQuant output data such as Lotgi1 entries grouped together because of sequence identity, number of sequence-unique and non-unique peptides, sequence coverage, protein length and molecular weight, PEP values and gel slice origin of peptides for protein identification. Click here for file

Additional file 14 **Peptides identified in acid-insoluble matrix C.** Xls-file containing MaxQuant output data concerning peptides, such as peptide sequence, mass, score, PEP and distribution among gel slices. Click here for file

Additional file 15 **Selected sequence alignments.** Doc-file showing sequence alignments of ependymin-related protein, gigasin-2, nacrein-like protein, tyrosinase, UP2, and osteonectin to similar proteins identified in this study. Click here for file

Additional file 16 **Sequence analysis of BMSP-related***Lottia***proteins.** Doc-file showing the alignment of BMSP-related protein sequences to *Mytilus galloprovincialis* BMSP (A) and the domain distribution in these sequences (B). Click here for file

Additional file 17 **Sequence analysis of Pif-related***Lottia***proteins.** Doc-file showing the alignment of Pif-related protein sequences to *Pinctada fucata* Pif (A) and the domain distribution in these sequences (B). Click here for file
