# Supplementary material for: In-depth proteomic analysis of a mollusc shell: acid-soluble and acid-insoluble matrix of the limpet Lottia gigantea
Source: Proteome Sci. 2012 Jun 13;10:28. doi: 10.1186/1477-5956-10-28 (PMC3374290; doi:10.1186/1477-5956-10-28)
Supplement: Additional file 1 — Lottia giganteaacid-soluble matrix proteins. Doc-file containing a list of all accepted protein identifications, their distribution in matrices obtained after different sodium hypochlorite treatments, the number of unique peptides, emPAI values and previously known or predicted subcellular occurrence. [file 1477-5956-10-28-S1.doc]

***Additional file 1: Lottia gigantea* acid-soluble matrix proteins**

| **Protein** | **Accession** | **Cleaning** | **Unique peptides** | **emPAI** |  | **Sub-cellular**  **location** |
| --- | --- | --- | --- | --- | --- | --- |
|  |  |  |  |  |  |  |
| Uncharacterized protein; domain: Cu-Zn superoxide dismutase, fragment | Lotgi1|101611 | A^2^  B^2^  C^2^ | 5  7  6 | 24.1  38.8  157.5 |  | IC |
| Similar to pancreatic lipase-related protein; domain: esterase/lipase/thioesterase | Lotgi1|102397 | A^2^  B^2^  C^2^ | 2  -  2 | 0.5  0.5 |  | EC |
| Similar to calumenin; domains: multiple EFh | Lotgi1|103470 | A  B  C | 9  6  7 | 2.7  1.7  1.7 | si/tm | EC/IC (ER) |
| Uncharacterized protein, fragment/similar to *M. edulis* collagen; 10% Tyr | Lotgi1|104132 | A  B  C | 2  -  - | 1.4 |  | EC |
| Similar to gelsolin; shares 1 peptide with Lotgi1|214936 | Lotgi1|105757 | A^2^  B^2^  C^2^ | 5  -  - | 1.4 |  | IC |
| Similar to ezrin/radixin/moesin | Lotgi1|106937 | A^2^  B^2^  C^2^ | 19  10  12 | 19.7  2.8  5.8 |  | IC |
| Similar to fascin; domains: fascin | Lotgi1|108036 | A^2^  B^2^  C^2^ | 6  -  - | 4.6 |  | IC |
| Uncharacterized protein, fragment?; domain: IG_like (c2) | Lotgi1|108898 | A^2^  B^2^  C^2^ | 8  8  7 | 9.0  5.3  3.0 |  | EC |
| Similar to intermediate filament protein; shares peptides with Lotgi1|204921 | Lotgi1|109284 | A^2^  B^2^  C^2^ | 14  -  4 | 1.6  0.2 |  | IC |
| Similar to pancreatic lipase-related protein; domain: esterase/lipase/thioesterase | Lotgi1|109426 | A^2^  B^2^  C^2^ | 3  2  3 | 1.2  0.7  1.8 |  | EC |
| Similar to osteonectin/BM-40/SPARC, overlapping fragments; domains: EFh, KAZAL, | Lotgi1|109908  Lotgi1|176394 (aa1-135) | A^2^  B^2^  C^2^ | 19  13  16 | 74.0  16.8  35.5 | si/tm | EC |
| Uncharacterized protein/similar to mucin-like protein 1 fragment | Lotgi1|110884 | A^2^  B^2^  C | 7  6  3 | 62.1  14.8  3.0 | si | EC |
| Similar to thiopurine S-methyltransferase; domain: TPMT | Lotgi1|112328 | A  B  C | 2  -  2 | 0.6  0.6 |  | IC |
| Similar to follistatin-like protein; domains: EFh, Kazal_FS | Lotgi1|112867 | A  B  C | 3  4  4 | 0.6  1.9  1.2 |  | EC |
| Uncharacterized protein/similar to antistasin; domains: antistasin; 15% Cys | Lotgi1|113221 | A^2^  B^2^  C^2^ | 4  -  3 | 2.2  0.8 | si | EC |
| Uncharacterized protein/similar to 45kDa Calcium-binding protein; domains: EFh | Lotgi1|113682 | A  B  C | 4  -  - | 1.0 |  | IC (Golgi lumen) |
| Similar to lysozyme, g-type | Lotgi1|114561 | A^2^  B^2^  C^2^ | 18  22  22 | 2781.6  9999.0  >10,000 |  | EC |
| Uncharacterized protein; domain: Dyp-type peroxidase | Lotgi1|114599 | A^2^  B  C | 6  2  - | 1.1  0.3 |  | ? |
| Uncharacterized protein; 6 repeats of ~30aa, starting with MITPE; 14% Pro, 11% Thr | Lotgi1|115147 | A^2^  B^2^  C^2^ | 10  11  8 | 27.5  14.2  14.2 |  | ? |
| Uncharacterized protein/similar to epidermal growth factor receptor kinase substrate 8; domains: SH3, PH | Lotgi1|115450 | A^2^  B^2^  C^2^ | 6  -  - | 0.5 |  | IC |
| Uncharacterized protein/similar to ceruloplasmin; domains: cupredoxin, multicopper oxidase type 2 | Lotgi1|115607 | A^2^  B^2^  C^2^ | 44  31  36 | 48.0  10.8  24.4 |  | EC |
| Similar to legumain; domain:peptidase C13/legumain | Lotgi1|115714 | A^2^  B^2^  C^2^ | 3  -  3 | 0.4  0.4 | si | EC |
| Uncharacterized protein; domains: Cys-rich secretory protein (SCP), Ves allergen | Lotgi1|116834  Lotgi1|116974 | A  B  C | 2  -  2 | 3.2  1.4 |  | EC |
| Similar to phospholipase A2/group 3 secreted PLA2 | Lotgi1|117176 | A  B  C | 2  -  - | 3.6 |  | EC |
| Uncharacterized protein/similar to mucin-like protein 1/2 (fragment) | Lotgi1|121410 | A  B  C | 3  -  - | 2.2 |  | EC |
| Uncharacterized protein/similar to mucin-like protein 1/2 (fragment) | Lotgi1|121599 | A  B  C | 2  -  - | 1.5 |  | EC |
| Similar to transcriptional activator pur-α | Lotgi1|121721 | A  B  C | -  -  2 | 0.3 |  | IC |
| Similar to nucleobindin-2; domains: EFh | Lotgi1|121860 | A  B  C | 18  18  18 | 85.6  47.7  47.7 |  | IC/EC |
| Similar to ribosomal protein P2 | Lotgi1|121932 | A  B  C | 6  -  2 | 13.7  1.2 |  | IC |
| Similar to matrix metalloproteases-21; domain: ZnMc_MMP, hemopexin | Lotgi1|122269 | A^2^  B^2^  C^2^ | 4  4  5 | 0.4  0.4  0.6 |  | EC |
| Uncharacterized protein; domain: DUF938 | Lotgi1|123440 | A  B  C | 3  -  - | 0.9 |  | ? |
| Similar to Kunitz-type protease inhibitor kalicludein-3/SHPI-1 and -2 | Lotgi1|123902 | A  B  C | 2  -  - | 3.6 |  | EC |
| Similar to multicopper oxidase; domains: multicopper oxidase type 1/2 | Lotgi1|124263 | A^2^  B^2^  C^2^ | 9  2  3 | 1.2  0.2  0.3 |  | ? |
| Uncharacterized protein; 12% Lys; domain: SOUL/ heme-binding superfamily | Lotgi1|125538 | A  B  C | 3  -  - | 0.3 |  | ? |
| Similar to ubiquitin/polyubiquitin | Lotgi1|126004  Lotgi1|234561  Lotgi1|162671  Lotgi1|233138 | A^2^  B^2^  C^2^ | 3  3  4 | 24.1  14.8  24.1 |  | IC |
| Similar to semaphorin-2a; domain: semaphorin/CD100 | Lotgi1|126264 | A  B  C | -  -  2 | 0.3 |  | TM/EC |
| Similar to Myo/myotrophin; domain: ankyrin | Lotgi1|128209 | A  B  C | 3  -  - | 1.7 |  | IC |
| Uncharacterized protein; domain: RHOD (rhodanese) superfamily; 16% Glu; pI 4.7 | Lotgi1|129390 | A^2^  B^2^  C | 5  5  5 | 9.0  5.8  9.0 |  | ? |
| Similar to thioredoxin-domain containing protein; domains: thioredoxin-related | Lotgi1|131935 | A^2^  B^2^  C^2^ | 15  13  15 | 7.9  4.4  9.0 |  | IC (ER) |
| Similar to Kunitz-type protease inhibitor KCP_HALAI ^1^ | Lotgi1|132911 | A^2^  B  C | 3  2  3 | 4.6  2.2  4.6 |  | EC |
| Similar to dermatopontin ^1^ | Lotgi1|133595 | A^2^  B^2^  C^2^ | 5  -  4 | 9.0  5.0 | si | EC |
| Similar to DnaJ/HSP40; domains: DnaJ, DnaJ_C | Lotgi1|138864 | A  B  C | 7  6  7 | 1.4  1.8  1.4 |  | IC |
| Similar to BMSP ^1^; domains: vWFA | Lotgi1|140660 | A^2^  B^2^  C^2^ | 7  7  7 | 30.6  41.2  30.6 |  | EC |
| Similar to aldehyde reductase; domains: aldoketo_reductase | Lotgi1|140773 | A^2^  B^2^  C^2^ | 8  -  - | 2.2 |  | IC |
| Similar to membrane alanine aminopeptidase; domain: M1_APN_2 | Lotgi1|140786 | A^2^  B^2^  C^2^ | 37  33  32 | 17.7  9.0  7.3 |  | IC (ER) |
| Similar to ribosomal protein L27 | Lotgi1|141455 | A  B  C | 3  -  - | 4.6 |  | IC |
| Similar to transaldolase | Lotgi1|142681 | A^2^  B^2^  C^2^ | 2  -  - | 0.3 |  | IC |
| Uncharacterized protein; 25% Gln, 10% Glu, 17% Pro, 12% Val, 10% Asn, 10% Leu; 6 short repeats:k/qQQPxVELNKQQP; pI 5.2 | Lotgi1|142790 | A  B  C | 5  5  4 | 67.1  45.4  45.4 |  | ? |
| Uncharacterized protein; 38% Gln, 11% Leu, 10% Pro; 5 ~70aa repeats containing shorter repeat motifs like NQQQ and KQQQ | Lotgi1|142814 | A^2^  B^2^  C^2^ | 11  11  12 | 3.9  14.4  16.8 |  | ? |
| Similar to perlwapin ^1^; domains: WAP (4x) | Lotgi1|143247 | A^2^  B  C | 6  6  7 | 3.6  3.6  5.0 |  | EC |
| Similar to sulfatase-1 | Lotgi1|143759 | A  B  C | 3  -  - | 0.5 |  | IC (ER/Golgi) |
| Similar to gelsolin/villin | Lotgi1|144968 | A^2^  B^2^  C^2^ | 3  -  - | 1.7 |  | IC |
| Similar to histone H3 | Lotgi1|176498  etc | A^2^  B^2^  C^2^ | 2  -  3 | 2.2  -  2.2 |  | IC |
| Similar to histone H2A | Lotgi1|181153  etc | A^2^  B^2^  C^2^ | 3  3  3 | 3.6  3.6  3.6 |  | IC |
| Similar to calmodulin; domains: EFh, recoverin; N-term acetyl-Ala | Lotgi1|148903  Lotgi1|190032  Lotgi1|203998  Lotgi1|216228 | A  B  C | 2  -  2 | 2.2  1.2 |  | IC |
| Similar to ribosomal protein S12 | Lotgi1|150191 | A^2^  B  C | 4  -  - | 6.2 |  | IC |
| Similar to DnaJ/HSP40; domains: DnaJ, TPR repeat | Lotgi1|151060 | A  B  C | 3  5  3 | 0.5  1.0  0.5 |  | IC |
| Similar to acyl-protein thioesterase; domain: abhydrolase/lysophospholipase_related | Lotgi1|152136 | A  B  C | -  2  2 | 0.6  0.6 |  | IC |
| Uncharacterized protein; 20% Gly, 12% Pro | Lotgi1|152688 | A  B  C | 3  2  3 | 99.0  2.2  30.6 | tm | TM |
| Uncharacterized protein; domains: Sushi/SRC/CCP (complement control protein), PAN | Lotgi1|152699 | A  B  C | 5  4  2 | 3.6  1.8  0.7 | si | EC |
| Uncharacterized protein; 11% Pro | Lotgi1|152799 | A^2^  B  C^2^ | 27  26  28 | 3161.3  718.7  2274.8 | si | EC |
| Similar to paramyosin fragment, N-term  Similar to paramyosin fragment, C-term | Lotgi1|153233  Lotgi1|153235 | A  B  C  A  B  C | 7  -  5  3  -  4 | 1.7  1.1  3.0  9.0 |  | IC |
| Uncharacterized protein; 10% Leu, 11% Ser | Lotgi1|153653 | A  B  C | 2  2  4 | 0.4  0.4  0.9 |  | ? |
| Uncharacterized protein; 11% Glu; pI: 4.3; 3 repeats starting at aa 491, 551, and 611 | Lotgi1|154020 | A^2^  B^2^  C^2^ | 27  28  29 | 60.1  77.1  91.1 | si/tm | EC/TM |
| Uncharacterized protein | Lotgi1|154423 | A^2^  B^2^  C^2^ | 7  2  3 | 25.8  1.7  4.2 | si/tm | EC/TM |
| Uncharacterized protein | Lotgi1|154424 | A^2^  B^2^  C^2^ | 7  4  4 | 30.6  3.2  2.2 | si | EC |
| Uncharacterized protein; domain: NTR(netrin)_like superfamily/meteorin | Lotgi1|154590 | A^2^  B^2^  C^2^ | 3  5  6 | 0.5  1.1  1.4 | si/tm | EC |
| Uncharacterized protein; domain: CBM6(mannanase_like)-CBM35-CBM36_like superfamily | Lotgi1|154713 | A^2^  B^2^  C^2^ | 2  2  2 | 4.6  4.6  4.6 | si/tm | EC/TM |
| Uncharacterized protein; domain: Tryp_SPc superfamily, trypsin-like serine protease | Lotgi1|155299 | A  B  C | 6  -  2 | 1.6  0.3 |  | EC |
| Uncharacterized protein; domains: CLECT, CUB, Sushi/CCP, LDLRA_2, EGF; pI: 4.6 | Lotgi1|156525 | A  B^2^  C | 11  4  8 | 1.4  0.3  1.0 | tm | TM |
| Uncharacterized protein; pI 4.7; domains: CCP, Hyalin, Tyrosine-protein kinase ephrin type A/B receptor-like | Lotgi1|156526 | A  B  C | 12  -  5 | 1.5  0.6 |  | ? |
| Similar to FAM20C/DMP4 (dentin matrix protein 4) | Lotgi1|156599 | A^2^  B^2^  C^2^ | 14  15  14 | 6.9  7.9  5.3 |  | EC |
| Uncharacterized protein; 12% Leu, 12% Lys, pI 9.4 | Lotgi1|156601 | A  B^2^  C | 3  3  3 | 1.2  1.2  1.2 | si/tm | EC/TM |
| Uncharacterized protein; domains: Kazal-type protease inhibitor; 10% Leu; pI: 5.5 | Lotgi1|157133 | A  B  C | -  2  - | 1.7 | si | EC |
| Uncharacterized protein, 13% Val, shares peptide with Lotgi1|157827 | Lotgi1|157680 | A^2^  B^2^  C | 3  -  - | 2.7 | si | EC |
| Uncharacterized protein; 13% Val, 12% Asp, 10% Ala, pI 4.6; domain: EFh | Lotgi1|157689 | A  B  C | 5  5  4 | 30.6  99.0  55.2 | si | EC |
| Uncharacterized protein; 13% Val, 11% Asp, pI 4.5 | Lotgi1|157690 | A  B^2^  C | 5  4  4 | 24.1  9.0  14.8 | tm | TM |
| Uncharacterized protein; 11% Val; shares peptide with Lotgi1|157680 | Lotgi1|157827 | A^2^  B^2^  C^2^ | 6  4  4 | 67.1  9.0  9.0 | si/tm | EC/TM |
| Similar to cytochrome c oxidase subunit 5A | Lotgi1|157909 | A  B  C | 2  -  - | 0.5 |  | IC (Mito) |
| Uncharacterized protein; 11% Pro; Q-rich C-term (aa210-240) | Lotgi1|158113 | A^2^  B^2^  C^2^ | 13  15  15 | 1583.9  5010.9  1583.9 |  | ? |
| Uncharacterized protein; 11% Ser, 10% Gln; Q/S-rich motif aa561-640 | Lotgi1|158316 | A^2^  B^2^  C^2^ | 51  55  56 | 159.0  243.2  354.6 | si | EC |
| Uncharacterized protein; domains: β-ketoacyl synthase, acyl transferase, NAD(P)-binding, β-ketoacyl reductase | Lotgi1|158379 | A^2^  B^2^  C^2^ | 6  -  - | 0.1 |  | IC |
| Uncharacterized protein;13% Pro, 12% Ser | Lotgi1|158905 | A^2^  B  C^2^ | 18  13  22 | 288.4  57.8  491.4 |  | ? |
| Uncharacterized protein; domain: partial phospholipase_A2_3 | Lotgi1|159173 | A  B  C | 5  4  5 | 3.4  2.4  2.4 | tm | TM |
| Uncharacterized protein | Lotgi1|159264 | A^2^  B^2^  C^2^ | 10  9  4 | 1.2  1.2  0.4 | si/tm | EC/TM |
| Similar to buccalin; sequence consists mainly of nineteen ~14aa repeats of the type KRGxDxf/yFxGQLG | Lotgi1|159314 | A^2^  B  C | 15  3  - | 315.3  1.0 | si/tm | EC |
| Uncharacterized protein; 26% Gln, 13% Leu, 12% Thr, pI 4; starting with aa 156 8x SNLLQQPDa/tTQqLa/tTNeQQQ | Lotgi1|159331 | A  B  C | 2  2  2 | 9.0  9.0  9.0 | si | EC |
| Uncharacterized protein; domains: FAD-linked oxidase, arabinono-1,4-lactone oxidase | Lotgi1|159383 | A^2^  B^2^  C^2^ | 5  -  - | 0.9 |  | ? |
| Uncharacterized protein; domains: SRCR, chitin-binding | Lotgi1|159694 | A^2^  B  C | 30  9  36 | 0.8  0.2  1.1 |  | EC/TM |
| Uncharacterized protein; domains: SEA, 19% Thr, 13% Ser, 10% Ala | Lotgi1|159695 | A  B  C | 4  -  - | 0.7 | tm | EC/TM |
| Uncharacterized protein | Lotgi1|159735 | A^2^  B^2^  C | 6  -  - | 2.5 |  | ? |
| Uncharacterized protein; domains: Chitin-binding_2 peritrophin A | Lotgi1|160173 | A^2^  B^2^  C^2^ | 12  12  12 | 358.4  277.3  463.2 | si | EC |
| Uncharacterized protein; 13% Gln, 11% Gly, 11% Thr | Lotgi1|160356 | A^2^  B^2^  C^2^ | 7  5  4 | 9.0  4.0  3.0 |  | ? |
| Similar to Cys-rich secretory protein-2; domains: CAP/allergen V5/Tpx1-related | Lotgi1|160396 | A  B  C^2^ | -  -  2 | 0.7 |  | EC |
| Similar to 84kDa HSP/HSP90A | Lotgi1|161608 | A^2^  B^2^  C^2^ | -  -  3 | 0.2 |  | IC |
| Uncharacterized protein; 12% Pro; G/P-rich C-term (aa90-103) | Lotgi1|162562 | A^2^  B^2^  C^2^ | 5  2  4 | 55.2  2.2  16.8 | si | EC |
| Uncharacterized protein; 14% Ser, 10% Thr | Lotgi1|162861 | A^2^  B^2^  C^2^ | 16  15  16 | 5.0  7.5  7.4 | si/tm, tm | TM |
| Similar to thioester-containing protein; domains: α2-macroglobulin | Lotgi1|162872 | A^2^  B^2^  C^2^ | 53  47  52 | 9.3  6.8  8.7 |  | EC |
| Uncharacterized protein | Lotgi1|163448 | A^2^  B^2^  C^2^ | 17  18  19 | 43.7  49.1  55.2 | si | EC |
| Uncharacterized protein; domain: EFh, 17% Asp, 16%Ala, pI: 3.8; 12 ~30aa repeats | Lotgi1|163637 | A^2^  B^2^  C^2^ | 23  16  18 | 83.8  18.3  21.8 | si | EC |
| Uncharacterized protein/similar to endo α-1,4 polygalactosaminidase; domain: DUF297 | Lotgi1|163670 | A^2^  B^2^  C^2^ | 6  4  6 | 3.6  6.7  5.0 | si/tm | EC/TM |
| Uncharacterized protein; 16% Ser, 11% Thr, 11% Pro;  pI: 4.9 | Lotgi1|164956 | A  B  C | 2  2  3 | 0.9  0.9  1.3 | si | EC |
| Uncharacterized protein; 11% Asp, pI 3.8; domains: CCP | Lotgi1|164986 | A  B  C | 2  -  - | 2.2 | si | EC |
| Uncharacterized protein; domain: EGF_like; shares peptides with Lotgi1|165458/234475 | Lotgi1|165388 | A  B  C | 5  -  4 | 0.5  0.4 |  | ? |
| Similar to superoxide dismutase [Cu-Zn] | Lotgi1|166129 | A  B  C | -  -  2 | 0.4 | si | EC |
| Similar to USP (universal stress protein) | Lotgi1|166166 | A  B  C | 3  -  - | 1.7 |  | IC |
| Similar to tyrosinase ^1^; 11% Pro; domain: tyrosinase; aa393-462 nine GPPVNP-type repeats | Lotgi1|166196 | A^2^  B^2^  C^2^ | 8  -  4 | 0.9  0.4 | si | EC |
| Uncharacterized protein; 10% Ser | Lotgi1|166451 | A^2^  B^2^  C^2^ | 9  6  9 | 14.8  6.9  19.0 |  | ? |
| Similar to peroxiredoxin | Lotgi1|169267 | A  B  C | 5  -  - | 3.2 |  | IC (Mito) |
| Uncharacterized protein; 14% Pro, 11% Thr | Lotgi1|169439 | A  B  C | 2  -  - | 0.3 | si | EC |
| Uncharacterized protein; domain: AdoMet_MTase superfamily | Lotgi1|169491 | A^2^  B^2^  C^2^ | 3  2  - | 0.9  0.5 | si/tm | ? |
| Uncharacterized protein/similar to meteorin | Lotgi1|169717 | A  B  C | 2  -  3 | 0.6  1.0 | si/tm | EC |
| Uncharacterized protein | Lotgi1|169925 | A  B  C | 2  2  - | 4.6  2.2 | si | EC |
| Uncharacterized protein; 13% Ser, G/E block aa322-337; pI 4.4 | Lotgi1|171084 | A  B^2^  C | 4  3  3 | 99.0  99.0  45.4 | si | EC |
| Uncharacterized protein | Lotgi1|171839 | A  B  C | 11  10  9 | 16.8  25.1  16.8 |  | ? |
| Similar to antistasin; 17% Cys; domains: antistasin; limited similarity to aa660-950 of lustrin A ^1^ | Lotgi1|171918 | A  B  C | 10  8  12 | 18.3  5.1  15.4 | si | EC |
| Similar to cysteine protease/cathepsin F; domain: Peptidase_C1A | Lotgi1|172186 | A^2^  B  C | 3  -  - | 0.6 |  | IC (Lyso) |
| Similar to cystatin; domains: CY | Lotgi1|172187 | A^2^  B^2^  C^2^ | 2  -  2 | 1.2  0.7 | si | EC |
| Uncharacterized protein; 23% Glu, 13% Asn, 13% Ser; aa130-702: 31 x 14aa repeats similar to QSNQQFNxxQSNQQF | Lotgi1|172698 | A^2^  B^2^  C^2^ | 16  16  15 | 11.5  8.0  10.2 | si/tm | EC/TM |
| Similar to actin-related protein 2/3 complex subunit; domain: p16-Arc | Lotgi1|172988 | A  B  C | 4  -  - | 3.6 |  | IC |
| Similar to BMSP/Pif ^1^, fragment; domain: CBM_14 (chitin-binding) peritrophin A | Lotgi1|173138 | A^2^  B^2^  C^2^ | 5  6  4 | 24.1  24.1  9.0 |  | EC |
| Uncharacterized protein; 14% Pro; pI: 4.7 | Lotgi1|173199 | A  B  C | 8  2  4 | 1.4  0.4  0.7 |  | ? |
| Uncharacterized protein; ~10% of P, N and G; aa107-170: 10x GAMP/GSMP; limited similarity to mpn88 ^1^ | Lotgi1|173200 | A^2^  B^2^  C^2^ | 35  34  38 | 5622.4  2237.7  7942.3 |  | EC |
| Uncharacterized protein; shares peptides with Lotgi1|236952; domain: N/apple PAN/DNA-binding | Lotgi1|173550 | A  B  C | 3  -  2 | >0.1  >0.1 |  | IC (Nuc) |
| Uncharacterized protein; 15% Pro; 19% P in aa50-400 and 35% P in aa778-882 | Lotgi1|174003 | A^2^  B^2^  C^2^ | 49  47  46 | 473.8  240.2  183.0 | si/tm | EC/TM |
| Similar to perlustrin ^1^ | Lotgi1|174065 | A^2^  B^2^  C^2^ | 12  10  11 | <10,000  <10,000  <10,000 | si/tm | EC |
| Uncharacterized protein; 22% Gln, 12% Pro; aa704-848: 8 ~16aa repeats similar to LNQQQPPVQLNQQQPP | Lotgi1|174203 | A  B  C | 14  13  12 | 9.0  5.8  6.7 | si/tm | EC/TM |
| Uncharacterized protein; 12% Pro, 10% Ser | Lotgi1|174652 | A^2^  B^2^  C^2^ | 46  49  46 | 12.6  13.2  14.4 | si/tm | EC/TM |
| Uncharacterized protein; domain: DUF187 | Lotgi1|174920 | A^2^  B^2^  C^2^ | 21  22  22 | 29.3  26.8  38.1 | si | EC |
| Uncharacterized protein; domain: DUF547 | Lotgi1|175346 | A^2^  B  C | 2  -  - | 0.4 |  | ? |
| Uncharacterized protein; domain: Apple-like, Exo-endo-phos, partial; 12% Leu | Lotgi1|175888 | A  B  C | -  -  2 | 0.8 |  | IC |
| Similar to histone H2B/H4 | Lotgi1|175997 | A^2^  B^2^  C^2^ | 10  8  11 | 9.0  3.9  19.3 |  | IC |
| Similar to embryocarcinomic antigen-related cell adhesion molecule (secreted CEACAM1a-4C1); domains: IG_like | Lotgi1|176496 | A  B^2^  C | 2  2  3 | 1.2  1.2  1.8 |  | EC/TM |
| Uncharacterized protein; 13% Ser, 13% Val, 12% Gly | Lotgi1|176686 | A^2^  B^2^  C^2^ | 3  2  2 | 9.0  3.6  3.6 | si | EC |
| Uncharacterized protein; 13% Cys, 10% His; zinc_finger signature (CH2H2_1) | Lotgi1|176773 | A  B  C | 3  -  - | 5.3 |  | IC (Nuc) |
| Similar to NEDD8; domain: ubiquitin | Lotgi1|177660 | A  B  C | 2  -  - | 3.6 |  | IC |
| Similar to HSP70; shares 1 peptide with Lotgi1|198956 | Lotgi1|177837 | A^2^  B^2^  C^2^ | 2  -  - | 0.2 |  | IC |
| Similar to ferritin | Lotgi1|178880 | A^2^  B^2^  C^2^ | 12  12  11 | 16.8  16.8  13.7 |  | IC |
| Similar to pleiotrophic membrane chitin-binding protein/chitin deacetylase; domain: polysaccharide deacetylase | Lotgi1|181237 | A^2^  B^2^  C^2^ | 9  5  4 | 3.3  1.1  1.1 | si | EC |
| Similar to ribosomal protein S13 | Lotgi1|181799 | A  B  C | 4  -  - | 2.2 |  | IC |
| Similar to enolase | Lotgi1|182683 | A^2^  B  C^2^ | 4  -  - | 0.6 |  | IC |
| Similar to arginine kinase | Lotgi1|183052 | A^2^  B^2^  C | 13  5  - | 9.0  1.1 |  | IC |
| Similar to profilin; N-term acetyl-Ser | Lotgi1|183446 | A^2^  B^2^  C^2^ | 5  3  4 | 9.0  2.2  3.6 |  | IC |
| Similar to rho GDP dissociation factor | Lotgi1|183614 | A^2^  B^2^  C^2^ | 6  -  6 | 6.7  2.6 |  | IC |
| Similar to ribosomal protein S29 | Lotgi1|183859 | A  B  C | 2  -  - | 3.6 |  | IC |
| Similar to protein disulfide isomerase | Lotgi1|184255 | A^2^  B^2^  C^2^ | 3  -  - | 0.3 | si | IC (ER)/ EC |
| Uncharacterized protein; 11% Ser, 11% Leu | Lotgi1|185419 | A  B  C | -  -  2 | 1.2 | si/tm | EC/TM |
| Similar to syntenin; domains: PDZ | Lotgi1|186095 | A^2^  B^2^  C^2^ | -  -  2 | 0.4 |  | IC |
| Similar to palmitoyl-protein thioesterase | Lotgi1|186317 | A^2^  B^2^  C^2^ | 12  11  10 | 21.8  12.9  15.4 |  | IC (Lyso) |
| Similar to cytosolic malate dehydrogenase | Lotgi1|188534 | A^2^  B^2^  C^2^ | 8  -  - | 2.2 |  | IC |
| Similar to chloride intracellular channel protein/Glutathione-S-transferase, C-term | Lotgi1|189089 | A^2^  B^2^  C^2^ | 10  5  2 | 7.5  1.3  0.4 |  | IC |
| Similar to HSP70; shares 1 peptide with Lotgi1|209056 | Lotgi1|190284 | A  B  C | -  -  2 | 0.2 |  | IC |
| Similar to MSP130-2/alkaline phosphatase-like protein | Lotgi1|190352 | A^2^  B  C | 3  -  - | 0.3 |  | EC/TM |
| Similar to peptidylprolyl isomerase/FKBP2 | Lotgi1|192237 | A^2^  B^2^  C^2^ | 6  4  5 | 18.3  4.2  9.0 | si/tm | IC (ER) |
| Similar to gelsolin | Lotgi1|192582 | A^2^  B^2^  C^2^ | 2  -  - | 0.3 |  | IC |
| Similar to plancitoxin/DNAse II | Lotgi1|193211 | A^2^  B^2^  C^2^ | 2  3  - | 0.5  0.8 | si | EC |
| Similar to glutathione-S-transferase | Lotgi1|195517 | A  B^2^  C^2^ | 3  -  2 | 0.7  0.4 |  | IC |
| Uncharacterized protein/similar to NADH-ubiquinone oxidoreductase; 10% Arg; domain: NDUFB10 superfamily | Lotgi1|196202 | A  B  C | 3  -  - | 2.2 |  | IC (Mito) |
| Similar to ubiquinol-cyt c reductase | Lotgi1|197554 | A  B  C | 3  -  - | 1.7 |  | IC (Mito) |
| Similar to β-tubulin | Lotgi1|197703  Lotgi1|202077  Lotgi1|223043  Lotgi1|239523  Lotgi1|220547 | A^2^  B  C^2^ | 3  2  8 | 0.5  0.3  1.8 |  | IC |
| Similar to sulfatase | Lotgi1|197818 | A  B  C | 3  3  2 | 0.4  0.4  0.2 | si | IC (Golgi/ER) |
| Similar to ribosomal protein S10; domain: S10_plectin | Lotgi1|198455 | A  B  C | 4  -  - | 1.0 |  | IC |
| Similar to Na^+^/H^+^- exchange regulatory cofactor NHE-RF2; domain: PDZ_signaling | Lotgi1|198460 | A  B  C | 2  -  - | 0.9 |  | IC |
| Similar to ribosomal protein S13 | Lotgi1|198613 | A^2^  B^2^  C | 2  -  - | 9.0 |  | IC |
| Similar to ubiquitin carrier protein | Lotgi1|198904 | A  B  C | 2  -  - | 0.9 |  | IC |
| Similar to pyruvate kinase | Lotgi1|199626 | A^2^  B  C | 2  -  - | 0.2 |  | IC |
| Similar to ribosomal protein L31 | Lotgi1|200036 | A  B  C | 2  -  - | 1.5 |  | IC |
| Similar to perlwapin ^1^; domains: antistasin, WAP, 15% Cys, 11% Pro | Lotgi1|201804 | A  B  C | 22  11  12 | 8.4  0.9  1.0 | si | EC |
| Similar to ribosomal protein S3a | Lotgi1|202499 | A^2^  B  C | 2  -  - | 0.4 |  | IC |
| Similar to actin; shares peptides with contaminant (bovine actin) | Lotgi1|202971  Lotgi1|205506  Lotgi1|215510 | A^2^  B^2^  C^2^ | 4  4  6 | 0.7  1.0  2.0 |  | IC |
| Similar to fimbrin/plastin; domains: EFh, calponin, actinin | Lotgi1|203020 | A^2^  B^2^  C^2^ | 17  -  9 | 2.7  0.9 |  | IC |
| Similar to transgelin/calponin-like | Lotgi1|203275 | A  B  C^2^ | 4  -  2 | 1.2  0.5 |  | IC |
| Similar to HsjCib/β-thymosin-repeat protein; domains: thymosin β-4(5x) | Lotgi1|203463 | A  B  C | 5  -  - | 2.2 |  | IC |
| Similar to ribosomal protein S2 | Lotgi1|203487 | A^2^  B^2^  C^2^ | 5  -  - | 1.9 |  | IC |
| Similar to dipeptidyl peptidase 1/cathepsin | Lotgi1|203670 | A^2^  B  C^2^ | 2  -  - | 0.2 | si | IC (Lyso) |
| Uncharacterized protein; 11% Val, 10% Thr | Lotgi1|203715 | A  B  C | 2  -  3 | 3.0  5.3 | si/tm | EC/TM |
| Uncharacterized protein; 11% Asn | Lotgi1|203811 | A  B  C | 15  9  11 | 3.3  1.3  1.8 | si | EC |
| Similar to thioredoxin; N-term: acetyl-Ser | Lotgi1|203919 | A^2^  B^2^  C^2^ | 2  -  - | 2.2 |  | IC |
| Similar to ubiquitin carboxy-terminal hydrolase | Lotgi1|204019 | A  B  C | 6  -  2 | 12.3  0.8 |  | IC |
| Similar to ribosomal protein S17 | Lotgi1|204040 | A  B  C | 2  -  - | 1.5 |  | IC |
| Similar to peptidyl-glycine α-amidating monooxygenase | Lotgi1|204047 | A^2^  B^2^  C^2^ | 14  10  11 | 38.8  4.4  14.8 | si | EC |
| Similar to actin-related 2/3 complex subunit 4/ARP23 complex 20 kDa subunit | Lotgi1|204359 | A^2^  B^2^  C^2^ | 3  -  2 | 1.0  0.6 |  | IC |
| Uncharacterized protein; domain: USP (universal stress protein)_like | Lotgi1|204747 | A^2^  B^2^  C^2^ | 8  2  3 | 18.3  0.9  1.7 |  | IC |
| Uncharacterized protein; domains: EFh | Lotgi1|204915 | A^2^  B^2^  C | 7  4  5 | 12.9  4.2  6.2 |  | IC |
| Similar to intermediate filament protein; shares peptides with Lotgi1|109284 | Lotgi1|204921 | A^2^  B^2^  C | 3  -  - | 0.2 |  | IC |
| Uncharacterized protein; domain: SOUL | Lotgi1|205030 | A^2^  B^2^  C^2^ | 10  7  10 | 176.8  41.2  132.4 | si/tm | ? |
| Similar to β-catenin | Lotgi1|205205 | A  B^2^  C | 2  2  2 | 0.1  0.1  0.1 |  | IC |
| Similar to 14-3-3 protein B; shares peptide with Lotgi1|145846 and 222442 | Lotgi1|205379 | A^2^  B^2^  C^2^ | 5  2  - | 1.5  0.6 |  | IC |
| Similar to carbonic anhydrase | Lotgi1|205401 | A^2^  B  C^2^ | 8  3  5 | 7.3  0.8  2.2 |  | IC |
| Similar to nucleoside diphosphate kinase B | Lotgi1|205662 | A^2^  B^2^  C^2^ | 9  -  5 | 9.0  2.2 | si | ? |
| Similar to glutaminyl-peptide cyclotransferase; domain: peptidase_M28 | Lotgi1|206060 | A^2^  B^2^  C^2^ | 4  3  2 | 0.7  0.5  0.3 | si/tm | EC |
| Similar to ubiquitin-conjugating E2 | Lotgi1|207488 | A  B  C | 2  -  - | 0.9 |  | IC |
| Similar to tyrosine 3-monooxygenase activation protein/14-3-3 protein ε; shares 1 peptide with Lotgi1|145856 and 222442 | Lotgi1|207549 | A^2^  B^2^  C^2^ | 3  -  - | 0.6 |  | IC |
| Similar to ribonuclease UK114 | Lotgi1|208507 | A  B  C | 2  -  - | 1.5 |  | IC |
| Similar to chitobiase/chitinase | Lotgi1|209107 | A^2^  B^2^  C^2^ | 5  7  10 | 1.0  2.9  3.4 | si/tm | IC (Lyso) |
| Similar to thioester-containing protein/α2-macroglobulin | Lotgi1|209261 | A^2^  B^2^  C^2^ | 55  39  50 | 10.0  3.0  7.5 |  | EC |
| Similar to α-N-acetylgalactosaminidase | Lotgi1|209340 | A^2^  B^2^  C^2^ | 6  3  7 | 1.2  0.5  1.8 | si | IC (Lyso) |
| Similar to superoxide dismutase | Lotgi1|210191 | A  B  C | 3  -  - | 1.7 |  | IC |
| Similar to thioester-containing protein/α2-macroglobulin | Lotgi1|211452 | A^2^  B^2^  C^2^ | 2  -  - | 0.1 |  | EC |
| Similar to ribosomal protein L38 | Lotgi1|211882 | A  B  C | 2  -  - | 2.2 |  | IC |
| Similar to thioredoxin | Lotgi1|214285 | A  B  C | 2  -  - | 1.5 |  | IC |
| Similar to ectonucleotide pyrophosphatase/ phosphodiesterase/nucleotide pyrophosphatase | Lotgi1|214482 | A  B  C | 2  -  - | 0.3 | si/tm | TM |
| Similar to non-muscle myosin light chain | Lotgi1|215828 | A^2^  B  C | 2  -  - | 0.8 |  | IC |
| 78kDa glucose-regulated protein/HSP70; shares 1 peptide with Lotgi1|177837 | Lotgi1|216416 | A^2^  B^2^  C^2^ | -  -  3 | 0.2 | si | IC (ER, lumen) |
| Similar to ribosomal protein L12 | Lotgi1|217320 | A^2^  B^2^  C^2^ | 5  3  3 | 4.6  1.4  1.4 |  | IC |
| Similar to fructose-bisphosphate aldolase | Lotgi1|217342 | A^2^  B^2^  C^2^ | 6  -  - | 1.3 |  | IC |
| Similar to Lin-7-like protein B; domains:PDZ, L27 | Lotgi1|217446 | A  B  C | 2  -  - | 0.5 |  | IC/M |
| Similar to rab GDP dissociation inhibitor/Rab GDI protein | Lotgi1|218952 | A^2^  B^2^  C^2^ | 2  -  - | 0.3 |  | IC |
| Similar to annexin, type IV | Lotgi1|219490 | A^2^  B  C | 2  -  - | 0.3 |  | IC |
| Similar to annexin, type IV | Lotgi1|219492 | A^2^  B^2^  C^2^ | 4  -  - | 0.6 |  | IC |
| Similar to calmodulin | Lotgi1|219843 | A  B  C | 5  -  - | 5.0 |  | IC |
| Similar to lecithin:cholesterol acyltransferase/1-O-acylceramide synthase | Lotgi1|220528 | A  B  C | 2  3  - | 0.3  0.5 | si/tm | EC |
| Similar to proactivator polapeptide (prosaposin) | Lotgi1|221240 | A^2^  B^2^  C^2^ | 13  2  8 | 5.7  0.2  0.9 | si | EC |
| Similar to 14-3-3 ζ protein; shares 1 peptide with Lotgi1|205379 and 145856 | Lotgi1|222442 | A^2^  B^2^  C^2^ | 4  -  - | 0.8 |  | IC |
| GAPDH; shares 1 peptide with contaminant (bovine GAPDH) | Lotgi1|222542 | A^2^  B^2^  C^2^ | 6  -  - | 1.4 |  | IC |
| Similar to peptidyl-prolyl cis/trans isomerase | Lotgi1|222979 | A^2^  B^2^  C^2^ | 5  6  6 | 25.8  25.8  9.0 | si | IC (ER, lumen) |
| Similar to α-tubulin | Lotgi1|223558 | A^2^  B^2^  C^2^ | 7  5  7 | 2.2  0.7  0.4 |  | IC |
| Similar to ribosomal protein L5 | Lotgi1|224562 | A^2^  B^2^  C | 2  -  - | 0.5 |  | IC |
| Similar to dystroglycan | Lotgi1|224800 | A  B  C | 3  -  - | 0.3 | si/tm, tm | EC/TM |
| Similar to malate dehydrogenase | Lotgi1|225558 | A^2^  B^2^  C | 6  -  - | 1.0 |  | IC |
| Similar to peroxiredoxin 6 | Lotgi1|225601 | A^2^  B  C | 6  -  - | 1.7 |  | IC |
| Similar to epidymal secretory protein E1-like/ Niemann-Pick C2 protein ; 15% Val, 11% Ser, 10% Lys | Lotgi1|226175 | A^2^  B^2^  C^2^ | 4  -  3 | 2.7  1.7 | si/tm | EC |
| Uncharacterized protein; domain: chitin_binding_3 | Lotgi1|226726 | A^2^  B^2^  C^2^ | 11  11  12 | 16.8  11.1  29.5 | si/tm | EC/TM |
| Similar to villin-1/gelsolin | Lotgi1|227360 | A^2^  B^2^  C^2^ | 3  -  - | 0.4 |  | IC |
| Uncharacterized protein; 10% Pro, 11% Leu; aa17-126: 17% R+K, 12% P, 11% L; pI: 11 | Lotgi1|227783 | A^2^  B^2^  C^2^ | 16  6  15 | 6811.9  67.1  >10,000 | si | EC |
| Uncharacterized protein; 27% Thr; pI: 4.3; aa20-300: 42% T, 15% E; aa301-482: 16% R | Lotgi1|227996 | A^2^  B^2^  C^2^ | 5  2  6 | 6.5  0.8  12.3 | si, tm | EC/TM |
| Similar to Pif97/BMSP ^1^; domains: vWA, chitin-binding | Lotgi1|228264 | A^2^  B^2^  C^2^ | 11  10  8 | 3.2  2.5  1.4 | si | EC |
| Uncharacterized protein; 12% Pro, 10% Tyr | Lotgi1|228268 | A^2^  B^2^  C^2^ | 20  16  23 | 5335.7  5335.7  9999.0 | si/tm | EC/TM |
| Uncharacterized protein; 18% Arg, 11% Ser; pI 11.7; R/H/S-rich from aa103-150 (30% R, 12% H, 12% S) | Lotgi1|228385 | A^2^  B  C | 5  5  5 | 99.0  9.0  5.3 | si/tm | EC |
| Similar to VEGF; domain: PDGF; Thr-rich motif near C-term | Lotgi1|228640 | A  B  C | 4  -  2 | 0.7  0.3 |  | EC |
| Uncharacterized protein; 13% Gly, 11% Arg | Lotgi1|228700 | A  B  C | 4  -  3 | 5.3  3.0 | si | EC |
| Uncharacterized protein; domains: Cys-rich repeat; 12% Gln, 13% Pro | Lotgi1|228882 | A^2^  B^2^  C^2^ | 33  30  37 | 1332.5  999.0  5109.0 | si/tm | EC |
| Similar to perlucin-like protein ^1^ (aa1-156);  aa200-470: ZP_2 domain | Lotgi1|229175 | A^2^  B^2^  C^2^ | 22  18  20 | 41.6  18.8  22.5 | si | EC |
| Uncharacterized protein; domain: carbohydrate –binding CBM_6/Galactose-binding; 11% Ser | Lotgi1|229427 | A^2^  B^2^  C^2^ | 16  6  11 | 7.1  0.9  2.2 | si | EC |
| Uncharacterized protein | Lotgi1|229482 | A^2^  B^2^  C^2^ | 8  5  5 | 4.9  1.9  1.4 | si | EC |
| Uncharacterized protein; domain: SOUL/heme-binding | Lotgi1|229543 | A^2^  B^2^  C^2^ | 12  9  13 | 124.9  14.8  198.5 | si | EC |
| Uncharacterized protein; domains: stefin A/cystatin | Lotgi1|229619 | A  B  C | 2  -  - | 0.9 |  | IC |
| Similar to thioester-containing protein/CD109 antigen-like; domains: A2M_N, A2M_N_2 | Lotgi1|229818 | A^2^  B^2^  C^2^ | 16  2  15 | 2.2  0.1  2.9 | si/tm, tm | TM |
| Uncharacterized protein; domain: zinc finger , CCCH-type (aa~40-70); 17% Arg, 11% Ser, 10% Asp | Lotgi1|229978 | A  B  C | 6  4  6 | 0.4  0.2  0.5 |  | IC |
| Similar to Notch; domains: EGF, CUB, tyrosine kinase, complement control, Sushi | Lotgi1|230171 | A  B  C | 9  -  8 | 0.2  0.1 |  | IC/EC |
| Similar to ribosomal protein S9 | Lotgi1|230231 | A  B  C | 5  -  2 | 3.0  0.6 |  | IC |
| Similar to calcineurin; domain: EFh; D-rich motif (aa152-171) | Lotgi1|230492 | A^2^  B^2^  C^2^ | 12  12  14 | 371.8  516.9  9999.0 | si | EC |
| Similar to calcium-binding protein; domain: EFh | Lotgi1|230493 | A^2^  B^2^  C | 7  5  6 | 5.8  5.8  5.8 | si | EC |
| Uncharacterized protein; 16% Pro | Lotgi1|230510 | A^2^  B^2^  C^2^ | 12  12  14 | 598.5  773.3  2781.6 |  | ? |
| Similar to putative RNA-binding protein; 34% Gly; domain: K homology | Lotgi1|230619 | A  B  C | 5  -  2 | 4.0  0.6 |  | IC |
| Uncharacterized protein; 10% Gly, 10% Cys | Lotgi1|230689 | A  B  C | 11  7  7 | 1.0  0.5  0.5 | si | EC |
| Uncharacterized protein; domain: EFh; 15% Ala, 13% Val; pI: 5.0 | Lotgi1|230732 | A  B  C | 5  4  4 | 13.7  9.0  5.8 | si/tm | EC/TM |
| Similar to myosin regulatory light chain; domains: EFh | Lotgi1|230842 | A  B  C | 2  -  2 | 0.8  0.8 |  | IC |
| Similar to pacifastin; domains: VWC/pacifastin | Lotgi1|230854  Lotgi1|176463 | A^2^  B^2^  C^2^ | 5  4  6 | 45.4  45.4  45.4 |  | EC |
| Uncharacterized protein | Lotgi1|230881 | A^2^  B  C | 17  13  15 | 39.8  13.7  23.5 | si, tm | EC/TM |
| Similar to antistasin | Lotgi1|230991 | A  B  C | 3  -  - | 1.2 | si | EC |
| Similar to UP2 ^1^; 12% Leu, 10% Ala | Lotgi1|231009 | A^2^  B^2^  C^2^ | 10  7  8 | 9999.0  9999.0  9999.0 | si/tm | EC |
| Uncharacterized protein; 11% Gly, 10% Ser; limited similarity to aa1000-1390 of lustrin A ^1^ | Lotgi1|231010 | A  B  C | 13  13  13 | 10.7  12.6  10.7 | si | EC |
| Uncharacterized protein | Lotgi1|231046 | A^2^  B^2^  C^2^ | 14  13  13 | 463.2  358.4  358.4 | si | EC |
| Uncharacterized protein | Lotgi1|231065 | A  B  C | 3  -  - | 9.0 | si/tm | EC/TM |
| Uncharacterized protein; 19% Gly, 12% Pro, 10% Asn; pI: 4.6; aa400-481: 1% M, 19% G, 14% L, 11% P; aa300-400: 33% G, 10% S; similar to mpn88 ^1^ | Lotgi1|231186 | A  B  C | 11  9  10 | 561.3  175.8  1777.3 | si, tm | EC/TM |
| Uncharacterized protein; 18% Ser, 11% Thr; domain: EB | Lotgi1|231368 | A  B  C | 3  -  2 | 0.4  0.4 | si, tm | EC/TM |
| Uncharacterized protein; domains: 2 x chitin-binding peritrophin-A; some similarity to PIF/BMSP ^1^ | Lotgi1|231395 | A^2^  B^2^  C^2^ | 8  6  6 | 34.9  6.7  11.9 | si | EC |
| Uncharacterized protein; domain: EFh, shares peptides with Lotgi1|231427; 13% Val, pI: 5.0 | Lotgi1|231426 | A  B^2^  C | 2  4  2 | 2.2  9.0  2.2 | tm | TM |
| Uncharacterized protein; domain: EFh; shares peptides with Lotgi1|231426; 16% Val, pI:4.8 | Lotgi1|231427 | A  B^2^  C | -  -  2 | 2.2 | si/tm | EC/TM |
| Similar to twisted gastrulation; domain: Tsg | Lotgi1|231460 | A  B  C | 5  4  5 | 3.0  1.5  2.2 | si | EC |
| Similar to predicted matrix metalloproteinase; domains: ZnMc_MMP, HX, PG (peptidoglycan-binding) | Lotgi1|231475 | A  B  C^2^ | 7  -  - | 1.6 | si | EC |
| Uncharacterized protein; 17% Pro, 10% Leu, 10% Ser, pI: 4.2; aa30-120: 18% E, 12% A, 10% P, 10% S | Lotgi1|231509 | A  B  C | 5  5  3 | 30.6  9.0  4.6 | si | EC |
| Similar to USP-A | Lotgi1|231833 | A  B  C | 3  -  - | 1.2 |  | IC |
| Uncharacterized protein; domain: SEA; 27% Ser, 14% Thr | Lotgi1|231862 | A  B^2^  C^2^ | 2  -  - | 0.7 | tm | TM |
| Uncharacterized protein; domains: chitin-binding peritrophin A; Pro-rich extensin-like; aa470-600: 29% pro, 16% Thr, 12% Gln, 12% Asn | Lotgi1|231869 | A^2^  B^2^  C^2^ | 59  50  60 | 84.9  29.9  74.4 |  | EC |
| Uncharacterized protein; 15% Pro | Lotgi1|231884 | A  B  C | 2  -  - | 2.2 | si | EC |
| Similar to Pif/BMSP ^1^; domains: vWA, chitin-binding | Lotgi1|232022 | A^2^  B^2^  C^2^ | 5  -  2 | 0.5  0.2 |  | EC |
| Uncharacterized protein; domain: EGF_3; 13% Thr | Lotgi1|232671 | A  B  C | -  -  2 | 0.9 | si | EC |
| Uncharacterized protein; 11% Ser | Lotgi1|232714 | A^2^  B^2^  C^2^ | 21  17  28 | 3.0  1.9  5.8 | si, tm | TM |
| Uncharacterized protein; domains: EGF; 11% Pro | Lotgi1|232718 | A  B  C | 13  12  8 | 2.5  1.9  1.6 | si, tm | TM |
| Similar to tropomyosin | Lotgi1|233095 | A  B  C | 17  5  13 | 16.2  1.0  7.7 |  | IC |
| Uncharacterized protein; domains: CAP/VSTPX_like /protease_inhibitor_16 | Lotgi1|233199 | A^2^  B^2^  C^2^ | 28  24  30 | 1332.5  999.0  2737.4 | si | EC |
| Uncharacterized protein; domains: CAP/VSTPX_like/  protease_inhibitor_16 | Lotgi1|233200 | A^2^  B^2^  C^2^ | 48  39  48 | >10,000  >10,000  >10,000 | si | EC |
| Uncharacterized protein; domains: CAP/allergen V5 | Lotgi1|233201 | A  B^2^  C^2^ | 5  6  9 | 0.6  1.2  1.6 | si | EC |
| Similar to UDP-N-acetylglucosamine pyrophosphorylase-like | Lotgi1|233335 | A  B  C | 3  10  - | 0.2  2.2 |  | IC |
| Uncharacterized protein | Lotgi1|233348 | A^2^  B^2^  C^2^ | 12  10  12 | 5.1  2.7  3.8 | si/tm, tm | TM |
| Uncharacterized protein; 16% Gly, 15% Arg | Lotgi1|233392 | A  B  C | 3  -  3 | 2.2  2.2 | si/tm | EC/TM |
| Uncharacterized protein; Pro/Ala- and His-rich motifs in C-term | Lotgi1|233397 | A^2^  B^2^  C^2^ | 12  11  11 | 2681.7  516.9  1929.7 | si/tm | EC/TM |
| Similar to cofilin/actin-depolymerizing factor | Lotgi1|233408 | A^2^  B^2^  C^2^ | 8  4  6 | 30.6  1.3  5.6 |  | IC |
| Uncharacterized protein; 31% Asp, D+E=41%, pI 3.6;  Similar to very acidic proteins like aspein | Lotgi1|233420 | A^2^  B^2^  C^2^ | 11  9  10 | 1583.9  630.0  1583.9 | si/tm | EC |
| Uncharacterized protein; 17% Ala | Lotgi1|233451 | A^2^  B^2^  C^2^ | 2  2  2 | 9.0  3.6  3.6 | si/tm, tm | TM |
| Uncharacterized protein; 14% Gly, 13% Asn, 11%Arg, 11% Asp; similar to nacrein B3/B4 ^1^ | Lotgi1|233461 | A^2^  B^2^  C^2^ | 16  19  20 | 5.5  9.9  11.9 |  | EC |
| Similar to ependymin-related protein 1/X-box-binding protein; domains: ependymin | Lotgi1|233583 | A^2^  B^2^  C^2^ | 2  -  - | 0.5 | si | EC |
| Similar to glutathione-S-transferase µ | Lotgi1|233779 | A^2^  B^2^  C^2^ | 5  -  - | 3.6 | si | IC |
| Uncharacterized protein; 10% Lys | Lotgi1|233820 | A^2^  B  C^2^ | 4  -  - | 0.8 |  | IC |
| Similar to low density lipoprotein-receptor-related 12; domains: CUB, LDLα | Lotgi1|233824 | A  B  C | 4  -  4 | 0.4  0.4 | si | EC/TM |
| Uncharacterized protein | Lotgi1|233852 | A  B  C | 3  -  - | 1.7 |  |  |
| Similar to thioredoxin-domain-containing protein 12 | Lotgi1|234262 | A  B  C | 2  -  - | 0.7 | si | IC (ER, lumen) |
| Uncharacterized protein; 13% Ala, 11% Gly | Lotgi1|234386 | A^2^  B^2^  C^2^ | 7  5  5 | 9.0  5.0  5.0 | si/tm | EC/TM |
| Uncharacterized protein | Lotgi1|234387 | A^2^  B^2^  C^2^ | 13  13  14 | 288.4  141.5  202.1 |  | ? |
| Uncharacterized protein; domains: chitin-binding, peritrophin A | Lotgi1|234405 | A^2^  B^2^  C^2^ | 16  14  19 | 40.2  23.2  57.8 | si | EC |
| Uncharacterized protein; domains: multiple FN3; shares peptides with and 164518 | Lotgi1|234472  Lotgi1|168990  Lotgi1|165393 | A^2^  B  C | 22  3  8 | 1.2  0.1  0.3 |  | ? |
| Uncharacterized protein; 13% Cys, 10% Asn, 10% Gly; 34% identity to E4W3F5_HALDV, B3TK45_HALDV, B3TK76_HALDV | Lotgi1|234488 | A^2^  B^2^  C^2^ | 10  6  8 | 41.2  12.3  12.3 | si | EC |
| Similar to selenoprotein P | Lotgi1|234596 | A  B  C | 3  3  6 | 1.7  1.7  4.2 |  | EC |
| Uncharacterized protein; 18% Asp, 16% Arg, 14% Gly; many GDDR and related repeats; domain: superoxide dismutase; very similar to acidic shell proteins such as aspein ^1^, DGRP_HALAI ^1^, or the aragonite-binding domain of Pif ^1^ | Lotgi1|234845 | A^2^  B  C^2^ | 4  4  13 | 1.5  1.2  17.5 | si | EC |
| Similar to melanotransferrin; domains: transferrin | Lotgi1|234865 | A^2^  B^2^  C^2^ | 17  6  12 | 1.3  0.3  0.8 | si | EC |
| Uncharacterized protein; domain: Sushi; 19% Gln, 11% Pro; 42% Q in aa281-630; G/L/A-rich region aa631-928; protein different from Lotgi1|234884 retrieved from annotation homepage! | Lotgi1|234884 | A^2^  B^2^  C^2^ | 15  15  15 | 397.1  189.5  315.2 | si | EC |
| Uncharacterized protein; 10% Gly | Lotgi1|234885 | A^2^  B^2^  C^2^ | 9  8  9 | >10,000  >10,000  >10,000 | si/tm | EC |
| Uncharacterized protein; domain: otoanchorin (aa~900-1100); 12% Asp, 12% Leu, pI 4.7 | Lotgi1|235120 | A^2^  B^2^  C^2^ | 44  34  41 | 65.1  6.4  54.4 |  | ? |
| Uncharacterized protein; 11% Ala, 13% Pro; aa120-247: 20% P, 16% A, 10% Q | Lotgi1|235497 | A^2^  B^2^  C^2^ | 21  20  23 | >10,000  >10,000  >10,000 | si/tm, tm | EC/TM |
| ~aa1-160: similar to perlucin-like protein ^1^ ;  aa220-480: ZP (zona pellucida)_2 | Lotgi1|235529 | A  B  C | 5  -  - | 0.6 |  | EC |
| Similar to gigasin-2 ^1^; domains: EGF, ZP_2 | Lotgi1|235548 | A^2^  B^2^  C^2^ | 10  8  8 | 4.3  3.1  3.6 | si, tm | EC |
| Similar to mannose receptor; domains: EGF, CLECT, ZP_2 | Lotgi1|235549 | A^2^  B^2^  C^2^ | 19  17  17 | 1.2  0.8  1.0 | si/tm, tm | EC/TM |
| Similar to interferon-γ-inducible lysosomal thiol reductase; domains: saposin A, GILT | Lotgi1|235566 | A  B  C | 4  3  3 | 1.5  1.5  1.0 | si | IC (Lyso) |
| Uncharacterized protein; 11% Arg, 12% Thr, 10% Ser | Lotgi1|235609 | A^2^  B^2^  C^2^ | 10  5  9 | 9.0  4.4  7.6 | si | EC |
| Uncharacterized protein; 15% Pro, 15% Thr | Lotgi1|235610 | A^2^  B^2^  C^2^ | 17  15  17 | >10,000  3161.3  7496.9 | si | EC |
| Uncharacterized protein; domain: annexin A7; 14% Gly, 13% Thr; 16 x [GGQPs/tT]; 44% identity to mpn88 in a ~100aa overlap | Lotgi1|235621 | A^2^  B^2^  C^2^ | 16  15  17 | >10,000  5178.5  >10,000 | si | EC |
| Similar to FGFBP; domain: FGFBP_1 | Lotgi1|235694 | A^2^  B  C^2^ | 5  4  6 | 11.9  5.0  9.0 | si/tm | EC |
| Uncharacterized protein; domain: EFh; 10% Leu, 10% Ser; pI: 4.5 | Lotgi1|235797 | A  B  C | -  -  2 | 1.5 | si | EC |
| Uncharacterized protein; 10% Asn, 18% Gln, 24% Pro; domains: Pro_rich_extensin; aa57-376: 17 repeats of 16aa, NNxa/vQPPxxQxxYQPx | Lotgi1|235812 | A  B  C | 16  13  13 | 27.9  13.3  16.0 |  | EC |
| Uncharacterized protein; 11% Ser | Lotgi1|235865 | A^2^  B^2^  C^2^ | 2  -  - | 0.3 | si | EC |
| Uncharacterized protein; 15% Ala | Lotgi1|235969 | A^2^  B^2^  C^2^ | 2  -  - | 2.2 | tm | TM |
| Uncharacterized protein; domain: Hedgehog/DD-peptidase | Lotgi1|235988 | A^2^  B^2^  C^2^ | 30  20  24 | 4.7  1.9  3.2 | si | EC |
| Similar to prefoldin subunit | Lotgi1|236136 | A  B  C | 2  -  - | 0.3 |  | IC |
| Uncharacterized protein; 13% Tyr (Y+F=18%) | Lotgi1|236183 | A^2^  B^2^  C^2^ | 15  15  15 | 3726.6  5178.4  3726.6 | si | EC |
| Similar to eukaryotic initiation factor 5A | Lotgi1|236304 | A^2^  B  C | 6  -  3 | 2.6  1.2 |  | IC |
| Uncharacterized protein; 19% Pro, 10% Ala, 10% Arg, 10% Val | Lotgi1|236689 | A^2^  B^2^  C^2^ | 12  17  13 | 9.0  21.8  12.9 | si | EC |
| Uncharacterized protein; 22% Gln, 19% Pro; aa268-356: 4 x [xAQPGAYQQP(x)_2-4_ GAYxQQP] | Lotgi1|236690 | A^2^  B^2^  C^2^ | 5  4  4 | 20.5  13.7  13.7 | si | EC |
| Uncharacterized protein; 22% Pro, 13% Gln, 10% Ala; Q-rich regions: ~aa61-160 and ~aa721-990; P-rich: ~aa280-600 and ~780-970 | Lotgi1|236691 | A^2^  B  C^2^ | 10  12  12 | 18.3  30.6  42.9 |  | ? |
| Uncharacterized protein;domains: ADAM_MEPRO | Lotgi1|236770 | A^2^  B^2^  C^2^ | 34  30  34 | 20.1  12.1  24.8 | si | EC |
| Uncharacterized protein; 12% Pro | Lotgi1|236812 | A  B  C | 13  17  20 | 1.0  1.3  1.7 |  | ? |
| Similar to ribosomal protein L30 | Lotgi1|236815 | A^2^  B  C | 4  -  2 | 9.0  1.2 |  | IC |
| Uncharacterized protein; domains: EGF, Vitellinogen, FN3; shares peptides with Lotgi1|173550 | Lotgi1|236952 | A^2^  B^2^  C^2^ | 57  10  40 | 0.5  0.1  0.3 | si | EC |
| Uncharacterized protein; domain: Glycohydrolase_26 | Lotgi1|236955 | A^2^  B^2^  C^2^ | 2  2  - | 0.5  0.5 | si | EC |
| Uncharacterized protein; 15% Ser, 14% Gly, 10% Arg;  Limited similarity to aa980-1420 of lustrin A ^1^ | Lotgi1|237013  Lotgi1|177036 | A  B  C | 4  -  8 | 0.3  0.8 | si | EC |
| Uncharacterized protein; domain: Lam (laminin)G/concanavalin A-like lectin | Lotgi1|237103 | A^2^  B^2^  C | 2  -  4 | 0.1  0.4 | si | EC |
| Uncharacterized protein; domain: EGF_1 | Lotgi1|237131 | A  B  C | 2  -  - | 1.2 | tm | TM |
| Similar to gastric intrinsic factor/transcobalamin; shares peptides with Lotgi1|237142 | Lotgi1|237143 | A^2^  B^2^  C^2^ | 3  3  3 | 24.1  9.0  14.8 | si | EC |
| Uncharacterized protein; domain: reelin; 21% Thr, 15% Ser, 11% Pro; aa190-300: 51% Thr | Lotgi1|237152 | A^2^  B  C^2^ | 4  2  4 | 1.6  0.5  1.6 | si/tm | EC |
| Similar to chitin-binding protein P86860 ^1^ | Lotgi1|237510 | A^2^  B^2^  C^2^ | 10  14  14 | 176.8  176.8  236.1 |  | EC |
| Similar to thrombospondin type 1-containing protein; domains:TSP_1, ADAM-TS spacer, PLAC (protease and lacunin) | Lotgi1|237754 | A  B  C | 7  7  5 | 0.7  0.7  0.5 |  | EC |
| Uncharacterized protein; 14% Pro,12% Gly | Lotgi1|237996 | A  B^2^  C^2^ | 3  3  3 | 99.0  99.0  99.0 | si/tm | EC/TM |
| Similar to nacrein-like protein ^1^; domain: α-carbonic anhydrase | Lotgi1|238082 | A^2^  B^2^  C^2^ | 43  38  48 | >10,000  >10,000  >10,000 | si/tm | EC |
| Uncharacterized protein; domain: DUF3421 | Lotgi1|238094 | A^2^  B^2^  C^2^ | 13  9  10 | 99.0  24.1  49.1 | si | EC |
| Similar to EGF receptor | Lotgi1|238156 | A  B  C | 5  2  5 | 1.9  0.4  1.2 | si | EC/TM |
| Uncharacterized protein; 12% Lys | Lotgi1|238301 | A  B  C | -  -  2 | 0.3 |  | ? |
| Similar to triosephosphate isomerase | Lotgi1|238326 | A^2^  B^2^  C^2^ | 2  -  - | 0.7 |  | IC |
| Uncharacterized protein; 19% Asp, 11%Val, 10% Asn, 10% Ala; D+E=25%; pI 3.6; some similarity to aspein ^1^ | Lotgi1|238358 | A^2^  B^2^  C^2^ | 10  10  9 | 41.2  41.2  41.2 | tm | TM |
| Uncharactrized protein; domains: vWA_ECM, chitin-binding CBM_14 (peritrophin A) | Lotgi1|238400 | A^2^  B^2^  C^2^ | 4  4  6 | 1.6  1.6  5.8 | si | EC |
| Uncharacterized protein | Lotgi1|238415 | A^2^  B^2^  C^2^ | 6  4  7 | 1.1  0.6  1.3 | si/tm | EC/TM |
| Similar to FKBP-type peptidyl-prolyl cis/trans isomerase | Lotgi1|238515 | A  B  C | 8  6  7 | 19.0  5.3  14.8 | si | IC (ER, lumen) |
| Similar to BMSP100 (aragonite-binding)^1^; 18% Gly, 12% Ser, 10% Thr; | Lotgi1|238526 | A^2^  B^2^  C^2^ | 36  40  34 | 48.5  76.4  45.4 |  | EC |
| Similar to aminopeptidase N; domain: peptidase M1_APN_2 | Lotgi1|238560 | A^2^  B^2^  C^2^ | 5  7  5 | 0.3  0.5  0.3 | si | EC |
| Uncharacterized protein; 12% Lys, 11% Val | Lotgi1|238760 | A^2^  B  C | 4  -  4 | 1.8  1.2 | si | EC |
| Uncharacterized protein; 13% Ala, 11% Arg, 11% Leu; KRA-rich C-terminus (aa185-219) | Lotgi1|238831 | A^2^  B^2^  C^2^ | 20  19  21 | 3510.2  999.0  3510.2 | si | EC |
| Similar to perlustrin ^1^; domain: IGFBP_N_2 | Lotgi1|238970 | A^2^  B^2^  C | 5  5  5 | 4640.6  99.0  214.4 | si | EC |
| Uncharacterized proteins; 14% Ser, 10% Gly ; 93% identical | Lotgi1|239005  Lotgi1|239006 | A^2^  B^2^  C^2^ | 5  2  5 | 6.2  0.9  9.0 | si | EC |
| Uncharacterized protein; domains: antistasin, WAP | Lotgi1|239125 | A^2^  B^2^  C^2^ | 78  72  82 | 544.6  482.3  1273.3 | si | EC |
| Uncharacterized protein; 10% Lys | Lotgi1|239159 | A^2^  B^2^  C^2^ | 5  2  3 | 9.0  1.4  2.2 | si/tm | EC/TM |
| Uncharacterized protein; 16% Gly, 12% Met, 10% Gln; Gly-rich motif aa30-65 | Lotgi1|239170 | A^2^  B^2^  C^2^ | 8  6  10 | >10,000  >10,000  >10,000 | si/tm, tm | TM |
| Uncharacterized protein; 18% Leu, 16% Gly, 12% Ala | Lotgi1|239171 | A  B  C | 2  3  - | 1.5  3.0 | si/tm, tm | TM |
| Uncharacterized protein; 18% Gly, 19% Met, 11% Leu; limited similarity to aa520-750 of mpn88 ^1^ | Lotgi1|239173 | A  B  C | -  -  2 | 1.2 | tm | TM |
| Uncharacterized protein; 20% Gly, 18% Met, 12% Ala, 10% Leu; some similarity to shematrins ^1^ | Lotgi1|239174 | A^2^  B^2^  C^2^ | 7  7  6 | >10,000  >10,000  >10,000 | si/tm, tm | M |
| aa1-420: similar to nacrein ^1^; domain: carbonic anhydrase  aa421-633: 26% Asp, 23% Gly, 22% Arg, 13% Asn; pI:4.8; similar to aspein ^1^ | Lotgi1|239188 | A^2^  B^2^  C^2^ | 15  16  20 | 84.8  63.9  153.0 | si | EC |
| Similar to alginate lyase | Lotgi1|239189 | A^2^  B^2^  C^2^ | 2  -  - | 0.6 | si/tm | EC/TM |
| Similar to elongation factor 1α | Lotgi1|239271 | A^2^  B^2^  C^2^ | 2  -  3 | 0.3  0.8 |  | IC |
| Uncharacterized protein; 10% Pro, 12% Ser, 13% Thr; Thr-rich motif from ~aa185-240 | Lotgi1|239339 | A^2^  B^2^  C^2^ | 28  26  28 | 99.0  64.8  167.8 |  | ? |
| Uncharacterized protein; domain: K homology (RNA-binding proteins;aa6-71); 35% Gly, 12% Arg | Lotgi1|239386 | A  B  C | 2  -  - | 0.5 |  | IC |
| Uncharacterized protein; 22% Gly, 12% Asn; aa30-105: 49% Gly, 26% Asn; some similarity to GAAP_HALAI ^1^ | Lotgi1|239447 | A^2^  B^2^  C^2^ | 8  9  8 | 3980.1  2510.9  1583.9 |  | ? |
| Uncharacterized protein; domain: EFh; 12% Lys, 11% Asp; 10% Arg | Lotgi1|239519 | A  B  C | 3  -  2 | 2.2  2.2 | si | EC |
| Similar to Pif/BMSP ^1^; domains: chitin_binding CBM_14/ peritrophin A; Thr-rich motif from aa300-372 | Lotgi1|239574 | A^2^  B^2^  C^2^ | 51  38  46 | 336.1  62.9  176.8 | si | EC |
| Similar to sulfate transporter/prestin_like; domains: Sulphate transporter, STAS | Lotgi1|53031 | A^2^  B  C | 2  -  - | 0.4 | tm | M |
| Similar to ezrin/radixin/moesin | Lotgi1|59617 | A  B  C^2^ | 2  -  - | 9.0 |  | IC |
| Similar to carbonic anhydrase; domain: α-carbonic anhydrase | Lotgi1|66515 | A  B  C | 5  4  5 | 20.5  11.9  15.7 |  | IC |
| Uncharacterized protein; domains: scavenger receptor_related (SRCR) | Lotgi1|69892 | A^2^  B^2^  C^2^ | 5  3  4 | 3.2  1.4  2.2 |  | EC/TM |
| Uncharacterized protein; 19% Pro, 15% Ser, 12% Gly; 9 x [g/dSQPGIYP] and 4 x imperfect; some similarity to adhesive plaque matrix protein | Lotgi1|77105 | A^2^  B^2^  C^2^ | 5  7  7 | 99.0  397.1  397.1 |  | EC |
| Similar to growth differentiation factor 11/myostatin; domains: TGF-β | Lotgi1|82990 | A  B  C | 7  4  5 | 2.2  0.9  1.3 |  | EC |
| Similar to hephaestin/ceruloplasmin; domain: multicopper oxidase, cupredoxin | Lotgi1|83160 | A^2^  B^2^  C^2^ | 17  12  13 | 11.7  6.0  9.0 |  | TM |
| Uncharacterized protein; 23% Asn, 15% Pro, 15% Thr, 11% Ser; 7 repeats similar to TPxxxNNVNPGSETPxTxNNVNPGSE and 2 incomplete | Lotgi1|84059 | A  B  C | 2  2  2 | 9.0  9.0  9.0 |  | ? |
| Uncharacterized protein; domain: CRD_FZ/frizzled Cys-rich | Lotgi1|89037 | A  B  C | 3  4  2 | 1.7  2.7  0.9 |  | EC/TM |
| Similar to Se-dependent glutathione peroxidase | Lotgi1|97333 | A  B  C | 4  2  2 | 4.2  0.9  0.9 |  | EC |
| Similar to pancreatic lipase-related; domain: lipase (fragment) | Lotgi1|98299 | A^2^  B^2^  C^2^ | 5  2  - | 3.2  0.8 |  | EC |
| Similar to pancreatic lipase-related; domain: lipase (fragment) | Lotgi1|98300 | A^2^  B^2^  C^2^ | 4  3  6 | 1.5  1.5  5.3 |  | EC |
| Uncharacterized protein; domain: An_peroxidase/ Peroxidase_3 | Lotgi1|99791 | A^2^  B^2^  C^2^ | 5  5  5 | 4.0  30.6  30.6 |  | ? |
| Uncharacterized protein; domain: An_peroxidase/ Peroxidase_3 | Lotgi1|99809 | A^2^  B^2^  C^2^ | 3  3  3 | 999.0  214.4  214.4 |  | ? |
| Uncharacterized protein; domain: An_peroxidase/ Peroxidase_3 | Lotgi1|99852 | A^2^  B^2^  C^2^ | 5  5  5 | 99.0  99.0  99.0 |  | ? |
|  |  |  |  |  |  |  |

The entries are ordered according to increasing accession numbers, with six digit numbers first, followed by few five digit numbers. ^1^, previously identified in mollusk shells. ^2^, also identified in acid-insoluble matrix AS, Cleaning of shells before de-mineralization: A, 2h sodium hypochlorite; B, 2h sodium hypochlorite plus two 5min ultrasound treatments; C, 24h sodium hypochlorite plus two 5min ultrasound treatments. emPAI was calculated for sequence- (group) unique peptides. However, entries identified by overlapping peptide sets were counted as one if sequence alignment indicated that these entries were from the same protein. Si, predicted secretion signal sequence; tm, predicted transmembrane segment(s); si/tm indicates overlapping predicted sites. EC, extracellular; IC, intracellular; TM, transmembrane; M, membrane; subcellular location according to predicted signal sequences, transmembrane sequences, similarity to other proteins, previous identification in other shell matrices, or Lotgi1 annotation (<http://genome.jgi-psf.org/pages/search-for-genes.jsf?organism=Lotgi1>). The most abundant proteins (average emPAI>1000) are shaded yellow.
