# Supplementary material for: In-depth proteomic analysis of a mollusc shell: acid-soluble and acid-insoluble matrix of the limpet Lottia gigantea
Source: Proteome Sci. 2012 Jun 13;10:28. doi: 10.1186/1477-5956-10-28 (PMC3374290; doi:10.1186/1477-5956-10-28)
Supplement: Additional file 2 — Lottia giganteaacid-insoluble matrix proteins. Doc-file containing a list of all accepted protein identifications, their distribution in matrices obtained after different sodium hypochlorite treatments, the number of unique peptides, emPAI values and previously known or predicted subcellular occurrence. [file 1477-5956-10-28-S2.doc]

***Additional file 2: Lottia gigantea* acid-insoluble matrix proteins**

| **Protein** | **Accession** | **Cleaning** | **Unique peptides** | **emPAI** |  | **Sub-cellular**  **location** |
| --- | --- | --- | --- | --- | --- | --- |
|  |  |  |  |  |  |  |
| Uncharacterized protein; domain: Cu-Zn superoxide dismutase, fragment | Lotgi1|101611 | A^2^  B^2^  C^2^ | 4  4  4 | 157.5  38.8  38.8 |  | IC |
| Similar to pancreatic lipase-related protein; domain: esterase/lipase/thioesterase | Lotgi1|102397 | A^2^  B  C^2^ | 5  6  6 | 1.6  5.8  3.6 |  | EC |
| Similar to LIN-24-like family member; domain: Aerolysin/hemolysin toxin | Lotgi1|103152 | A  B  C | 4  3  - | 1.3  1.3 |  | EC |
| Similar to Ran-1-prov | Lotgi1|104069 | A  B  C | 3  2  3 | 1.0  0.6  1.0 |  | IC |
| Similar to lipoma HMGIC fusion partner-like | Lotgi1|105456 | A  B  C | 2  -  - | 2.2 | si/tm, tm | M |
| Similar to gelsolin; shares 1 peptide with Lotgi1|214936 | Lotgi1|105757 | A^2^  B  C | 8  9  6 | 6.0  7.4  3.9 |  | IC |
| Similar to adenosylhomocysteinase | Lotgi1|105848 | A  B  C | 8  4  2 | 1.7  0.5  0.2 |  | IC |
| Similar to cadherin | Lotgi1|106548 | A  B  C | -  -  2 | 0.3 |  | TM |
| Similar to ezrin/radixin/moesin, fragment (N-term) | Lotgi1|106937 | A^2^  B^2^  C^2^ | 15  14  14 | 13.4  7.9  6.0 |  | IC |
| Similar to ras-related protein 2 | Lotgi1|107327 | A  B  C | 2  -  - | 0.5 |  | IC |
| Similar to ezrin/radixin/moesin fragment (C-term) | Lotgi1|59617  Lotgi1|107438 | A^2^  B  C | -  -  3 | 9.0 |  | IC |
| Similar to fascin; domains: fascin (4x) | Lotgi1|108036 | A^2^  B  C | 6  4  4 | 9.0  3.2  3.2 |  | IC |
| Uncharacterized protein, fragment?; domain: IG_like (c2) | Lotgi1|108898 | A^2^  B^2^  C^2^ | 5  3  2 | 2.2  1.0  0.6 |  | EC |
| Similar to intermediate filament protein; shares peptides with Lotgi1|204921 | Lotgi1|109284 | A^2^  B  C^2^ | 20  10  6 | 3.8  1.1  0.4 |  | IC |
| Similar to pancreatic lipase-related protein; domain: esterase/lipase/thioesterase | Lotgi1|109426 | A^2^  B^2^  C^2^ | 3  3  5 | 2.6  1.2  9.0 |  | EC |
| Similar to osteonectin/BM-40/SPARC, overlapping fragments; domains: EFh, KAZAL | Lotgi1|109908  Lotgi1|176394 (aa1-135) | A^2^  B^2^  C^2^ | 9  11  6 | 5.5  9.0  2.2 | si/tm | EC |
| Uncharacterized protein/similar to mucin-like protein 1 fragment | Lotgi1|110884 | A^2^  B^2^  C^2^ | 4  5  - | 9.0  14.8 | si | EC |
| Uncharacterized protein; domains: VWF; 34% identity in a 180aa overlap with *Mytilus* byssal fibre proximal thread matrix protein | Lotgi1|112071 | A  B  C | -  -  2 | 0.5 |  | EC |
| Uncharacterized protein/similar to antistasin; domains: antistasin; 15% Cys | Lotgi1|113221 | A^2^  B  C^2^ | 3  3  3 | 0.8  0.8  0.8 | si | EC |
| Similar to probable polyketide synthase 1/β-ketoacyl synthase | Lotgi1|113304 | A  B  C | 2  -  2 | 0.5  0.5 |  | IC (Mito) |
| Similar to lysozyme, g-type | Lotgi1|114561 | A^2^  B^2^  C^2^ | 11  12  11 | 34.9  99.0  76.4 |  | EC |
| Uncharacterized protein; domain: Dyp-type peroxidase | Lotgi1|114599 | A^2^  B^2^  C | 3  -  - | 0.4 |  | ? |
| Similar to coronin; domains: WD-40 repeats | Lotgi1|114901 | A  B  C | 2  -  - | 0.3 |  | IC |
| Uncharacterized protein; 6 repeats of ~30aa, starting with MITPE; 14% Pro, 11% Thr | Lotgi1|115147 | A^2^  B^2^  C^2^ | 7  5  7 | 9.0  4.3  7.1 |  | ? |
| Uncharacterized protein/similar **to** epidermal growth factor receptor kinase substrate 8; domains: SH3, PH | Lotgi1|115450 | A^2^  B  C | 5  8  4 | 0.4  0.8  0.3 |  | IC |
| Uncharacterized protein/similar to ceruloplasmin; domains: cupredoxin, multicopper oxidase type 2 | Lotgi1|115607 | A^2^  B^2^  C^2^ | 42  39  42 | 53.7  67.1  29.3 |  | EC |
| Similar to legumain; domain: peptidase C13/legumain | Lotgi1|115714 | A^2^  B  C^2^ | 4  4  5 | 0.6  0.6  0.7 | si | EC |
| Similar to basic leucine zipper and W2-containing protein 1; domain: eIF4-γ/eIF5/eIF2-ε/W2/ARM | Lotgi1|115834 | A  B  C | 3  -  - | 0.3 |  | IC |
| Uncharacterized protein; domain: Peptidase_M24 ; N-term acetyl-Ala | Lotgi1|115903 | A  B  C | 2  -  - | 0.3 |  | EC |
| Similar to signal sequence receptor β-like protein; domain: translocon-associated β | Lotgi1|118304 | A  B  C | 4  4  4 | 2.2  2.2  2.2 | si, tm | IC (ER, membrane) |
| Similar to arrestin | Lotgi1|120072 | A  B  C | 5  8  - | 0.8  1.6 |  | IC |
| Similar to S-formylglutathione hydrolase | Lotgi1|120210 | A  B  C | 5  2  2 | 1.1  0.3  0.3 |  | IC |
| Similar to matrix metalloproteases-21; domain: ZnMc_MMP, hemopexin | Lotgi1|122269 | A^2^  B^2^  C^2^ | 8  12  13 | 1.1  2.3  2.3 |  | EC |
| Similar to polypeptide N-acetylgalactosaminyl transferase; domains: ricin_B_lectin, pp_GalNAc-T | Lotgi1|123129 | A  B  C | -  2  - | 0.2 |  | IC (Golgi) |
| Similar to D-glucuronyl C5-epimerase; domain: C5_epim_C | Lotgi1|123168 | A  B  C | -  5  - | 0.8 |  | IC (Golgi) |
| Similar to peroxiredoxin-2; domain: alkyl hydroxyperoxide reductase | Lotgi1|123611 | A  B  C | -  2  2 | 0.2  0.3 |  | IC |
| Similar to ferritin | Lotgi1|123691 | A  B  C | 2  2  2 | 0.8  0.8  0.8 |  | IC |
| Similar to multicopper oxidase; domains: multicopper oxidase type 1/2 | Lotgi1|124263 | A^2^  B^2^  C^2^ | 11  4  4 | 1.2  0.4  0.4 |  | ? |
| Similar to ubiquitin/polyubiquitin | Lotgi1|126004  Lotgi1|234561  Lotgi1|162671  Lotgi1|233138 | A^2^  B^2^  C^2^ | 3  3  3 | 24.1  24.1  14.8 |  | IC |
| Similar to transmembrane emp24 domain-containing protein 9 | Lotgi1|128992 | A  B  C | 2  3  - | 0.8  1.4 | si, tm | IC/TM |
| Uncharacterized protein; domain: RHOD (rhodanese) superfamily; 16% Glu; pI 4.7 | Lotgi1|129390 | A^2^  B^2^  C^2^ | 2  3  - | 1.2  2.2 |  | ? |
| Similar to thioredoxin-domain containing protein; domains: thioredoxin-related | Lotgi1|131935 | A^2^  B^2^  C^2^ | 14  13  15 | 7.9  6.8  7.9 |  | IC (ER) |
| Similar to enolase-phosphatase E1 | Lotgi1|132223 | A  B  C | 3  -  - | 0.8 |  | IC |
| Similar to proteasome subunit β | Lotgi1|132853 | A  B  C | 5  4  - | 1.6  1.2 |  | IC |
| Similar to Kunitz-type protease inhibitor KCP_HALAI ^1^ | Lotgi1|132911 | A^2^  B^2^  C^2^ | 2  -  - | 2.2 |  | EC |
| Similar to dermatopontin ^1^ | Lotgi1|133595 | A^2^  B  C^2^ | 5  5  4 | 11.9  6.7  9.0 | si | EC |
| Similar to vitellogenic serine carboxypeptidase | Lotgi1|134569 | A  B  C | 3  -  5 | 0.4  0.8 |  | EC |
| Similar to NADH-ubiquinine oxidoreductase | Lotgi1|135579 | A  B  C | 2  -  - | 0.7 |  | IC (Mito) |
| Similar to BMSP ^1^; domains: vWFA | Lotgi1|140660 | A^2^  B^2^  C^2^ | 8  8  8 | 2370.4  2370.4  2370.4 |  | EC |
| Similar to aldehyde reductase; domains: aldoketo_reductase | Lotgi1|140773 | A^2^  B  C | 5  4  3 | 1.1  0.8  0.5 |  | IC |
| Similar to membrane alanine aminopeptidase; domain: M1_APN_2 | Lotgi1|140786 | A^2^  B^2^  C^2^ | 55  56  51 | 347.1  315.2  168.5 |  | IC (ER) |
| Similar to short-chain dehydrogenase/reductase SDR | Lotgi1|141698 | A  B  C | 5  7  4 | 0.8  1.3  0.6 | si | EC |
| Similar to N-acylsphingosine amidohydrolase; domain: CBAH/acid ceramidase-like/Ntn_AC_NAAA | Lotgi1|142206 | A  B  C | 3  2  4 | 0.5  0.3  0.7 |  | IC (Lyso) |
| Similar to transaldolase | Lotgi1|142681 | A^2^  B  C | 8  6  9 | 1.6  1.1  2.8 |  | IC |
| Uncharacterized protein; 38% Gln, 11% Leu, 10% Pro; 5 ~70aa repeats containing shorter repeat motifs like NQQQ and KQQQ | Lotgi1|142814 | A^2^  B^2^  C^2^ | 2  2  2 | 0.3  0.3  0.5 |  | ? |
| Similar to perlwapin ^1^; domains: WAP (4x) | Lotgi1|143247 | A^2^  B^2^  C^2^ | 5  -  - | 1.8 |  | EC |
| Similar to gelsolin/villin | Lotgi1|144968 | A^2^  B  C | 4  3  3 | 4.2  1.7  2.7 |  | IC |
| Similar to histone H3 | Lotgi1|176498  etc | A^2^  B  C^2^ | 2  2  2 | 2.2  2.2  2.2 |  | IC |
| Similar to histone H2A | Lotgi1|181153  etc | A^2^  B^2^  C^2^ | 3  2  3 | 3.6  1.2  2.2 |  | IC |
| Similar to purple acid phosphatase_like; domain: MPP_PAPs | Lotgi1|150028 | A  B  C | 2  2  3 | 0.3  0.3  0.5 | si | EC |
| Uncharacterized protein; domain: esterase_lipase superfamily, Ndr | Lotgi1|150144 | A  B  C | 6  5  4 | 1.2  1.0  0.6 |  | ? |
| Similar to 6-phosphogluconate dehydrogenase | Lotgi1|150160 | A  B  C | 4  2  4 | 0.6  0.3  0.8 |  | IC |
| Similar to ribosomal protein S12 | Lotgi1|150191 | A^2^  B  C | 4  -  - | 2.7 |  | IC |
| Similar to thioredoxin peroxidase 2 | Lotgi1|150310 | A  B  C | 3  3  3 | 1.2  0.8  0.8 |  | IC |
| Similar to ribosomal protein S28 | Lotgi1|150772 | A  B  C | 2  -  - | 3.6 |  | IC |
| Uncharacterized protein; 11% Pro | Lotgi1|152799 | A^2^  B^2^  C^2^ | 3  -  11 | 0.6  6.2 | si | EC |
| Similar to proteasome subunit β-type | Lotgi1|153858 | A  B  C | 2  -  - | 0.6 |  | IC |
| Uncharacterized protein; 11% Glu; pI: 4.3; 3 repeats starting at aa 491, 551, and 611 | Lotgi1|154020 | A^2^  B^2^  C^2^ | 16  13  15 | 3.8  3.0  5.1 | si/tm | EC/TM |
| Uncharacterized protein; 10% Phe; pI:5.0 | Lotgi1|154166 | A  B  C | 2  3  3 | 1.2  3.1  2.2 | si, tm | TM |
| Uncharacterized protein | Lotgi1|154423 | A^2^  B^2^  C^2^ | 7  5  4 | 18.3  9.0  4.2 | si/tm | EC/TM |
| Uncharacterized protein | Lotgi1|154424 | A^2^  B^2^  C^2^ | 6  3  5 | 9.0  2.2  4.6 | si | EC |
| Uncharacterized protein; domain: NTR(netrin)_like superfamily/meteorin | Lotgi1|154590 | A^2^  B^2^  C^2^ | 8  13  14 | 2.7  9.0  7.7 | si/tm | EC |
| Uncharacterized protein; pI:4.4 | Lotgi1|154712 | A  B  C | -  -  2 | 0.9 |  | ? |
| Uncharacterized protein; domain: CBM6(mannanase_like)-CBM35-CBM36_like superfamily | Lotgi1|154713 | A^2^  B^2^  C^2^ | 2  2  2 | 4.6  4.6  4.6 | si/tm | EC/TM |
| Uncharacterized protein; domains: CLECT, CUB, Sushi/CCP, LDLRA_2, EGF; pI: 4.6 | Lotgi1|156525 | A^2^  B^2^  C^2^ | -  2  - | 0.2 | tm | TM |
| Similar to FAM20C/DMP4 | Lotgi1|156599 | A^2^  B^2^  C^2^ | 6  8  6 | 1.0  1.5  1.5 |  | EC |
| Uncharacterized protein; 12% Leu, 12% Lys, pI 9.4 | Lotgi1|156601 | A^2^  B^2^  C^2^ | -  3  - | 1.2 | si, tm | EC/TM |
| Similar to apolipoprotein D; domain:lipocain | Lotgi1|156771 | A  B  C | -  -  2 | 1.2 | si | EC |
| Uncharacterized protein; 13% Val, 11% Asp, pI 4.5 | Lotgi1|157678  Lotgi1|157690 | A^2^  B^2^  C^2^ | -  2  - | 0.3 | tm | TM |
| Uncharacterized protein, shares 1 peptide with Lotgi1|157827; 13% Val | Lotgi1|157680 | A^2^  B  C | 2  2  - | 1.7  0.9 | si | EC |
| Uncharacterized protein; shares peptide with Lotgi1|157680; 11% Val | Lotgi1|157827 | A^2^  B^2^  C^2^ | 3  5  3 | 5.8  20.5  9.0 | si/tm | EC/TM |
| Uncharacterized protein; 11% Pro; Q-rich C-term (aa210-240) | Lotgi1|158113 | A^2^  B^2^  C^2^ | 7  8  8 | 4.0  14.8  14.8 |  | ? |
| Uncharacterized protein; 11% Ser, 10% Gln; Q/S-rich motif aa561-640 | Lotgi1|158316 | A^2^  B^2^  C^2^ | 7  15  7 | 0.4  1.2  0.4 | si | EC |
| Uncharacterized protein; domains: β-ketoacyl synthase, acyl transferase, NAD(P)-binding, β-ketoacyl reductase | Lotgi1|158379 | A^2^  B  C | 18  9  15 | 0.5  0.2  0.4 |  | IC |
| Similar to filamin; domains: Filamin/ABP280 repeat, calponin homology (CH) | Lotgi1|158607 | A  B  C | 6  -  - | 0.1 |  | IC |
| Uncharacterized protein;13% Pro, 12% Ser | Lotgi1|158905 | A^2^  B^2^  C^2^ | 2  -  5 | 0.4  1.4 |  | ? |
| Uncharacterized protein; domain: partial Phospholip_A2_3 | Lotgi1|159171  Lotgi1|159173  Lotgi1|176428 | A  B  C | 3  2  3 | 1.3  0.7  0.7 | tm | TM |
| Uncharacterized protein | Lotgi1|159264 | A^2^  B^2^  C^2^ | 17  21  19 | 5.2  10.7  6.3 | si/tm | EC/TM |
| Similar to buccalin; sequence consists mainly of nineteen ~14aa repeats of the type KRGxDxf/yFxGQLG | Lotgi1|159314 | A^2^  B^2^  C | 3  -  - | 1.0 | si/tm | EC |
| Uncharacterized protein; domains: FAD-linked oxidase, arabinono-1,4-lactone oxidase | Lotgi1|159383 | A^2^  B  C | 12  8  7 | 5.6  1.3  1.1 |  | ? |
| Uncharacterized protein; domains:, chitin-binding | Lotgi1|159694 | A^2^  B^2^  C^2^ | 2  -  - | >0.1 |  | EC/TM |
| Uncharacterized protein | Lotgi1|159735 | A^2^  B  C | 3  3  - | 0.9  1.3 |  | ? |
| Similar to HSP70 | Lotgi1|159745 | A  B  C | -  -  4 | 0.6 | si, tm | IC (ER) |
| Uncharacterized protein; domains: Chitin-binding_2 peritrophin A | Lotgi1|160173 | A^2^  B^2^  C^2^ | 10  8  10 | 26.8  11.9  26.8 | si | EC |
| Uncharacterized protein; 13% Gln, 11% Gly, 11% Thr | Lotgi1|160356 | A^2^  B^2^  C^2^ | 6  5  5 | 4.0  6.9  4.0 |  | ? |
| Similar to Cys-rich secretory protein; domain: V5/Tpx-1-related/CAP | Lotgi1|160396 | A  B  C^2^ | -  -  3 | 1.7 |  | EC |
| Similar to peptidase S8, S53, subtilisin, kexin, sedolisin | Lotgi1|160410 | A  B  C | -  2  2 | 1.2  1.2 | si | EC |
| Similar to 84kDa HSP/HSP90A | Lotgi1|161608 | A  B  C^2^ | 6  3  4 | 0.5  0.2  0.3 |  | IC |
| Uncharacterized protein; 12% Pro; G/P-rich C-term (aa90-103) | Lotgi1|162562 | A^2^  B^2^  C^2^ | 4  4  4 | 16.8  5.6  18.8 | si | EC |
| Uncharacterized protein; 14% Ser, 10% Thr | Lotgi1|162861 | A^2^  B^2^  C^2^ | 9  9  9 | 1.2  1.2  1.2 | si/tm, tm | TM |
| Similar to thioester-containing protein; domains: α2-macroglobulin | Lotgi1|162872 | A^2^  B^2^  C^2^ | 56  54  56 | 11.8  12.7  14.9 |  | EC |
| Similar to sodium/calcium exchanger/cellular retinoic acid/retinol-binding protein; domains: FABP/lipocalin/calycin | Lotgi1|163303 | A  B  C | 4  -  2 | 1.8  0.7 |  | IC |
| Uncharacterized protein | Lotgi1|163448 | A^2^  B^2^  C^2^ | 6  12  10 | 1.0  3.0  2.2 | si | EC |
| Similar to guanine nucleotide-binding protein subunit α; shares peptide with Lotgi1|226384 | Lotgi1|163596 | A  B  C | 4  -  - | 0.8 |  | IC |
| Uncharacterized protein; domain: EFh, 17% Asp, 16%Ala, pI: 3.8; 12 ~30aa repeats | Lotgi1|163637 | A^2^  B^2^  C^2^ | 18  21  19 | 371.8  99.0  60.1 | si | EC |
| Uncharacterized protein/similar to endo α-1,4 polygalactosaminidase; domain: DUF297 | Lotgi1|163670 | A^2^  B^2^  C^2^ | 6  5  9 | 11.9  3.6  58.9 | si/tm | EC/TM |
| Uncharacterized protein; domain: DUF672; 10% Leu | Lotgi1|164518 | A  B  C | -  -  2 | 0.4 |  | ? |
| Uncharacterized protein; domains: Sushi/Complement control module | Lotgi1|164755 | A  B  C | 2  3  2 | 0.9  1.7  0.9 | tm | TM |
| Similar to tyrosinase ^1^; 11% Pro; domain: tyrosinase; aa393-462 nine GPPVNP-type repeats | Lotgi1|166196 | A^2^  B  C^2^ | 4  4  2 | 0.4  0.5  0.2 | si | EC |
| Similar to proteasome subunit β-type 5,8 | Lotgi1|166223 | A  B  C | 3  -  - | 0.6 |  | IC |
| Uncharacterized protein; 10% Ser | Lotgi1|166451 | A^2^  B^2^  C^2^ | 2  3  4 | 0.6  2.2  1.5 |  | ? |
| Similar to voltage-dependent anion channel 2-like protein/porin | Lotgi1|168464 | A  B  C | 9  5  6 | 4.6  1.6  2.2 |  | IC (Mito) |
| Similar to programmed death protein; domain: DUF1241 superfamily | Lotgi1|168922 | A  B  C | 4  3  3 | 1.3  0.9  0.9 |  | IC |
| Similar to Na+/K+ -ATPase α subunit | Lotgi1|169374 | A  B  C | 4  -  - | 0.2 | tm | TM |
| Uncharacterized protein; domain: AdoMet_MTase superfamily | Lotgi1|169491 | A^2^  B^2^  C | 3  2  3 | 0.9  0.5  0.9 | si/tm | ? |
| Similar to citrate synthase | Lotgi1|170380 | A  B  C | 4  -  - | 0.5 |  | IC |
| Uncharacterized protein; 13% Ser, G/E block aa322-337; pI 4.4 | Lotgi1|171084 | A^2^  B^2^  C^2^ | -  2  - | 3.6 | si | EC |
| Similar to cysteine protease/cathepsin F; domain: Peptidase_C1A | Lotgi1|172186 | A^2^  B  C | 2  -  - | 0.4 |  | IC (Lyso) |
| Similar to cystatin; domains: CY | Lotgi1|172187 | A^2^  B  C^2^ | 3  3  5 | 1.8  1.8  3.6 | si | EC |
| Similar to emp24 domain-containing protein | Lotgi1|172331 | A  B  C | -  2  - | 1.2 | si/tm, tm | TM |
| Uncharacterized protein; 23% Glu, 13% Asn, 13% Ser; aa130-702: 31 x 14aa repeats similar to QSNQQFNxxQSNQQF | Lotgi1|172698 | A^2^  B^2^  C^2^ | 3  4  3 | 0.4  0.6  0.4 | si/tm | EC/TM |
| Similar to cathepsin D; Lotgi1|176001 possibly a fragment of 173095 | Lotgi1|173095  Lotgi1|176001 | A  B  C | 5  3  9 | 1.8  0.9  9.0 | si | EC |
| Similar to BMSP/Pif ^1^, fragment; domain: CBM_14 (chitin-binding) peritrophin A | Lotgi1|173138 | A^2^  B^2^  C^2^ | 10  10  10 | 3980.1  2510.9  999.0 |  | EC |
| Uncharacterized protein; ~10% of P, N and G; aa107-170: 10x GAMP/GSMP; limited similarity to mpn88 ^1^ | Lotgi1|173200 | A^2^  B^2^  C^2^ | 17  16  16 | 34.5  34.5  43.7 |  | EC |
| Uncharacterized protein; 15% Pro; 19% P in aa50-400 and 35% P in aa778-882 | Lotgi1|174003 | A^2^  B^2^  C^2^ | 36  38  38 | 33.8  70.3  65.6 | si/tm | EC/TM |
| Similar to perlustrin ^1^ | Lotgi1|174065 | A^2^  B^2^  C^2^ | 7  10  6 | 250.2  6308.6  250.2 | si/tm | EC |
| Uncharacterized protein; 12% Pro, 10% Ser | Lotgi1|174652 | A^2^  B^2^  C^2^ | 8  8  8 | 0.4  0.4  0.4 | si/tm | EC/TM |
| Uncharacterized protein; domain: DUF187 | Lotgi1|174920 | A^2^  B^2^  C^2^ | 25  30  30 | 107.9  214.4  549.5 | si | EC |
| Uncharacterized protein; domain:DUF547 | Lotgi1|175346 | A^2^  B  C | 3  -  - | 0.7 |  | ? |
| Similar to heparan sulfate 2-O-sulfotransferase | Lotgi1|175663 | A  B  C | -  2  2 | 0.8  1.4 |  | IC (Golgi) |
| Similar to histone H2B/H4 | Lotgi1|175997 | A^2^  B^2^  C^2^ | 7  6  11 | 3.9  2.5  13.3 |  | IC |
| Similar to embryocarcinomic atigen-related cell adhesion molecule (secreted CEACAM1a-4C1); domains 2x IG_like | Lotgi1|176496 | A^2^  B^2^  C^2^ | -  3  - | 1.8 |  | EC/TM |
| Uncharacterized protein; 13% Ser, 13% Val, 12% Gly | Lotgi1|176686 | A^2^  B^2^  C^2^ | 3  3  2 | 9.0  9.0  3.6 | si | EC |
| Uncharacterized protein; 20% Leu, 20% Gly, 10% Ala; pI:4.6 | Lotgi1|176992  Lotgi1|230170 | A  B  C | 2  2  2 | 3.6  3.6  9.0 | si, tm | TM |
| Similar to mitochondrial glutamate dehydrogenase | Lotgi1|177468 | A  B  C | 5  3  - | 0.6  0.3 |  | IC (Mito) |
| Similar to HSP70; shares peptides with Lotgi1|216416 | Lotgi1|177837 | A^2^  B  C | 8  3  2 | 0.9  0.3  0.2 |  | IC (ER) |
| Similar to inositol monophosphatase | Lotgi1|178401 | A  B  C | 2  -  - | 0.4 |  | IC |
| Similar to ferritin | Lotgi1|178880 | A^2^  B^2^  C^2^ | 12  13  12 | 11.1  16.8  11.1 |  | IC |
| Similar to proteasome subunit β-type | Lotgi1|181139 | A  B  C | 2  3  - | 0.5  0.8 |  | IC |
| Similar to pleiotrophic membrane chitin-binding protein/chitin deacetylase; domain: polysaccharide deacetylase | Lotgi1|181237 | A^2^  B^2^  C^2^ | 5  7  8 | 0.8  1.3  2.4 | si | EC |
| Similar to β-tubulin | Lotgi1|197703  Lotgi1|202077  Lotgi1|223043  Lotgi1|182889 | A^2^  B^2^  C^2^ | 3  -  13 | 0.5  9.0 |  | IC |
| Similar to enolase | Lotgi1|182683 | A^2^  B  C | 4  -  2 | 0.6  0.4 |  | IC |
| Similar to arginine kinase | Lotgi1|183052 | A^2^  B^2^  C | 7  3  - | 2.2  0.5 |  | IC |
| Similar to H^+^-transporting ATP synthase subunit | Lotgi1|183079 | A  B  C | 4  2  - | 1.4  0.4 |  | IC (Mito) |
| Similar to thioredoxin peroxidase 1/peroxiredoxin 4 | Lotgi1|183148 | A  B  C | 2  -  2 | 0.8  0.8 |  | IC/EC |
| Similar to profilin; N-term acetyl-Ser | Lotgi1|183446 | A^2^  B^2^  C^2^ | 4  4  4 | 3.6  3.6  3.6 |  | IC |
| Similar to rho GDP dissociation factor | Lotgi1|183614 | A^2^  B  C^2^ | 9  10  7 | 11.9  20.5  5.0 |  | IC |
| Similar to guanine nucleotide-binding protein β polypeptide 2-like | Lotgi1|184120 | A  B  C | 2  -  - | 0.3 |  | IC |
| Similar to Thr/Ser-protein phosphatase 2 | Lotgi1|184125 | A  B  C | 3  -  - | 0.8 |  | IC |
| Similar to protein disulfide isomerase | Lotgi1|184255 | A^2^  B  C | 13  10  11 | 2.7  1.9  1.9 | si | IC (ER) |
| Similar to adenosylhomocysteinase | Lotgi1|184532 | A  B  C | 4  -  2 | 0.6  0.3 |  | IC |
| Similar to S-acyl fatty acid synthase thioesterase | Lotgi1|184680 | A  B  C | 2  -  2 | 0.5  0.5 | tm | TM |
| Similar to syntenin | Lotgi1|186095 | A  B  C^2^ | 5  8  7 | 1.4  2.5  3.1 |  | IC |
| Similar to phospholipase B-like protein; domains: phospholip B_like | Lotgi1|186156 | A  B  C | -  -  3 | 0.4 | si | EC |
| Similar to ribosomal protein S8 | Lotgi1|186221 | A  B  C | -  2  - | 0.8 | si | ? |
| Similar to palmitoyl-protein thioesterase | Lotgi1|186317 | A^2^  B^2^  C^2^ | 6  7  6 | 2.7  3.4  3.4 |  | IC (Lyso) |
| Uncharacterized protein | Lotgi1|186451 | A  B  C | -  -  2 | 0.4 | si | EC |
| Similar to quinone reductase/ζ-crystallin | Lotgi1|187129 | A  B  C | -  3  - | 0.9 |  | IC |
| Similar to RAB; domains: small GTPase family | Lotgi1|187701 | A  B  C | 2  -  - | 0.5 |  | IC |
| Similar to phosphoglycerate mutase | Lotgi1|187919 | A  B  C | 6  2  - | 1.9  0.4 |  | IC |
| Similar to cytosolic malate dehydrogenase | Lotgi1|188534 | A^2^  B  C | 13  9  7 | 6.1  2.2  1.8 |  | IC |
| Similar to proteasome subunit α-type | Lotgi1|188929 | A  B  C | 2  -  - | 0.4 |  | IC |
| Similar to chloride intracellular channel protein/Glutathione-S-transferase, C-term | Lotgi1|189089 | A^2^  B^2^  C^2^ | 11  11  10 | 7.7  7.5  6.2 |  | IC |
| Similar to proteasome subunit α-type | Lotgi1|189380 | A  B  C | 2  2  4 | 0.6  0.6  1.5 |  | IC |
| Similar to α-actinin | Lotgi1|189716 | A  B  C | 4  -  2 | 0.2  0.1 |  | IC |
| Similar to MSP130-2/alkaline phosphatase-like protein | Lotgi1|190352 | A^2^  B  C | 3  -  - | 0.3 |  | EC/TM |
| Similar to thioredoxin-2/protein disulfide isomerase | Lotgi1|190816 | A  B  C | 7  -  - | 0.9 | si | EC |
| Similar to ribosomal protein S27 | Lotgi1|191640 | A  B  C | 3  2  - | 9.0  3.6 |  | IC |
| Similar to short-chain dehydrogenase/reductase SDR | Lotgi1|191692 | A  B  C | 3  4  - | 0.5  0.7 |  | IC |
| Similar to peptidylprolyl isomerase/FKBP2 | Lotgi1|192237 | A^2^  B^2^  C^2^ | 3  2  2 | 1.7  0.9  0.9 | si/tm | IC (ER) |
| Similar to alanine aminotransferase | Lotgi1|192553 | A  B  C | 3  -  - | 1.4 |  | IC |
| Similar to gelsolin | Lotgi1|192582 | A^2^  B  C | 12  11  10 | 3.0  4.0  2.5 |  | IC |
| Similar to proteasome subunit α-type | Lotgi1|192880 | A  B  C | 3  -  2 | 0.9  0.5 |  | IC |
| Similar to plancitoxin/DNAse II | Lotgi1|193211 | A^2^  B^2^  C | 5  4  6 | 1.6  1.2  2.8 | si | EC |
| Similar to actin; shares peptides with contaminant (bovine actin) | Lotgi1|193218  Lotgi1|202971  Lotgi1|215510  Lotgi1|205506 Lotgi1 | A^2^  B^2^  C^2^ | 5  5  5 | 0.5  0.5  0.5 |  | IC |
| Similar to Ras-related GTPase | Lotgi1|193895 | A  B  C | 2  -  - | 0.7 |  | IC |
| Similar to defender against apoptotic cell death 1 | Lotgi1|193969 | A  B  C | -  2  - | 2.2 | si/tm, tm | TM |
| Similar to guanine nucleotide-binding protein G, α-subunit | Lotgi1|194019 | A  B  C | 7  7  9 | 1.5  1.5  2.5 |  | IC |
| Similar to annexin A7 | Lotgi1|195070 | A  B  C | 2  -  5 | 0.3  1.0 |  | IC |
| Similar to glutathione-S-transferase | Lotgi1|195517 | A^2^  B  C^2^ | -  2  2 | 0.4  0.4 |  | IC |
| Similar to microsomal glutathione S-transferase; domain: membrane-associated proteins in eicosanoid and glutathione metabolism (MAPEG) | Lotgi1|196152 | A  B  C | 2  2  - | 0.9  0.9 | tm | IC (ER, tm) |
| Uncharacterized protein; domains: P67PHOX (3x), SH2 motif (4x) | Lotgi1|196407 | A  B  C | 2  2  2 | 0.8  0.8  0.8 |  | IC |
| Similar to ribophorin I | Lotgi1|196756 | A  B  C | 4  -  - | 0.3 | si/tm, tm | IC (ER, tm) |
| Similar to F-actin-capping protein subunit α | Lotgi1|196809 | A  B  C | 7  5  3 | 1.9  1.5  0.6 |  | IC |
| Uncharacterized protein; domain: translationally controlled tumor protein (TCTP) | Lotgi1|197001 | A  B  C | 4  -  2 | 5.3  3.0 |  | IC |
| Similar to dolichyl-diphosphooligosaccharide-protein glycosyltransferase | Lotgi1|197848 | A  B  C | 4  -  - | 1.3 | si/tm | IC (ER, tm) |
| Similar to Ras-related GTPase | Lotgi1|198201 | A  B  C | 4  3  2 | 0.9  0.6  0.4 |  | IC |
| Similar to ribosomal protein S13 | Lotgi1|198613 | A  B  C | 4  -  - | 2.2 |  | IC |
| Similar to eukaryotic translation elongation factor | Lotgi1|199050 | A  B  C | 12  -  - | 0.9 |  | IC |
| Similar to Cdc24-like protein/small GTPase family | Lotgi1|199128 | A  B  C | 2  -  2 | 0.8  0.8 |  | IC |
| Similar to pyruvate kinase | Lotgi1|199626 | A^2^  B  C | 8  -  - | 0.8 |  | IC |
| Similar to Ser/Thr-protein phosphatase; shares peptides with Lotgi1|192316 | Lotgi1|200510 | A  B  C | 5  4  - | 1.0  0.7 |  | IC |
| Similar to D-3-phosphoglycerate dehydrogenase | Lotgi1|200644 | A  B  C | 4  -  - | 0.6 |  | IC |
| Similar to ras-related Rab-1A; shares peptide with Lotgi1|205560 | Lotgi1|201174 | A  B  C | -  2  2 | 0.4  0.4 |  | IC |
| Similar to ATP synthase subunit β | Lotgi1|201878 | A  B  C | 16  16  13 | 4.4  3.9  2.8 |  | IC (Mito) |
| Similar to ribosomal protein S3a | Lotgi1|202499 | A^2^  B  C | 2  -  - | 0.4 |  | IC |
| Similar to CD9/tetraspanin | Lotgi1|202951 | A  B  C | -  -  2 | 1.2 | si/tm, tm | M |
| Similar to fimbrin/plastin; domains: EFh, calponin, actinin | Lotgi1|203020 | A^2^  B  C^2^ | 22  18  14 | 5.0  3.2  2.4 |  | IC |
| Similar to cAMP-regulated protein-like; domain: cofilin/ADF; N-term: acetyl-Ser | Lotgi1|203293 | A  B  C | 5  3  3 | 9.0  2.7  1.7 |  | IC |
| Similar to ribosomal protein SA | Lotgi1|203487 | A^2^  B  C | 6  3  3 | 2.5  0.7  0.7 |  | IC |
| Similar to dipeptidyl peptidase 1/cathepsin C | Lotgi1|203670 | A^2^  B  C | 4  -  8 | 0.5  1.2 | si | IC (Lyso) |
| Similar to dynein light chain, type 1 | Lotgi1|203845 | A  B  C | -  2  - | 1.5 |  | IC |
| Similar to transmembrane emp24 domain-containing protein 2 | Lotgi1|203874 | A  B  C | 3  -  - | 0.8 | si/tm, tm | TM |
| Similar to thioredoxin; N-term: acetyl-Ser | Lotgi1|203919 | A^2^  B  C | 3  2  2 | 4.6  2.2  2.2 |  | IC |
| Similar to peptidyl-glycine α-amidating monooxygenase | Lotgi1|204047 | A^2^  B^2^  C^2^ | 3  4  3 | 0.6  0.8  0.6 | si | EC |
| Similar to prohibitin | Lotgi1|204240 | A  B  C | 5  3  - | 0.8  0.4 | tm | IC (Mito, tm) |
| Similar to actin-related 2/3 complex subunit 4/ARP23 complex 20 kDa subunit | Lotgi1|204359 | A^2^  B  C^2^ | 4  3  5 | 1.5  1.0  2.2 |  | IC |
| Uncharacterized protein; domain: USP(universal stress protein)_like | Lotgi1|204747 | A^2^  B^2^  C^2^ | 8  8  7 | 50.8  25.8  12.9 |  | IC |
| Similar to translocon-associated protein subunit δ | Lotgi1|204770 | A  B  C | 4  2  3 | 9.0  2.2  4.6 | si/tm | IC (ER, membrane) |
| Uncharacterized protein; domains: EFh | Lotgi1|204915 | A^2^  B^2^  C^2^ | 5  3  - | 4.2  1.7 |  | IC |
| Similar to intermediate filament protein; shares peptides with Lotgi1|109284 | Lotgi1|204921 | A^2^  B  C | 12  4  - | 1.5  0.3 |  | IC |
| Uncharacterized protein; domain: SOUL | Lotgi1|205030 | A^2^  B^2^  C^2^ | 7  7  7 | 16.8  16.8  16.8 | si/tm | EC/TM |
| Similar to uroporphyrinogen decarboxylase | Lotgi1|205092 | A  B  C | 2  -  - | 0.3 |  | IC |
| Similar to β-catenin | Lotgi1|205205 | A^2^  B^2^  C^2^ | -  3  - | 0.2 |  | IC |
| Similar to 14-3-3 protein B; shares peptide with Lotgi1|145846 and 222442 | Lotgi1|205379 | A^2^  B^2^  C | 10  7  9 | 6.4  3.0  3.6 |  | IC |
| Similar to carbonic anhydrase | Lotgi1|205401 | A^2^  B^2^  C^2^ | 3  -  3 | 0.8  0.8 |  | IC |
| Similar to Ras-related Rab-10; shares 1 peptide with Lotgi1|201174 | Lotgi1|205560 | A  B  C | 3  2  - | 0.8 |  | IC |
| Similar to eukaryotic translation initiation factor 2 | Lotgi1|205563 | A  B  C | 3  -  2 | 0.5  0.3 |  | IC |
| Similar to nucleoside diphosphate kinase B | Lotgi1|205662 | A^2^  B  C^2^ | 7  4  4 | 5.3  2.2  1.5 | si | ? |
| Similar to tissue specific transplantation antigen P35B; domain: NAD-dependent epimerase/dehydratase | Lotgi1|205768 | A  B  C | 3  -  - | 0.6 |  | IC |
| Similar to glutaminyl-peptide cyclotransferase; domain: peptidase_M28 | Lotgi1|206060 | A^2^  B^2^  C^2^ | 9  9  11 | 2.9  2.9  4.8 | si/tm | EC |
| Similar to cathepsin A; domains: carboxypeptidase/peptidase_S10 | Lotgi1|206131 | A  B  C | -  2  2 | 0.4  0.4 | si/tm | IC (Lyso) |
| Similar to calreticulin | Lotgi1|206609 | A  B  C | 3  -  2 | 0.4  0.2 | si | EC |
| Similar to ATP synthase subunit α | Lotgi1|206617 | A  B  C | 13  10  7 | 1.5  0.9  0.6 |  | IC (Mito) |
| Similar to translation initiation factor eIF4 | Lotgi1|207101 | A  B  C | 9  6  4 | 1.6  0.9  0.5 |  | IC |
| Similar to Rho-like protein | Lotgi1|207113 | A  B  C | 2  -  - | 0.3 |  | IC |
| Similar to tyrosine 3-monooxygenase activation protein/14-3-3 protein ε; shares 1 peptide with Lotgi1|145856 and 222442 | Lotgi1|207549 | A^2^  B  C | 9  8  10 | 5.3  4.4  6.3 |  | IC |
| Similar to Ras-like GTPase | Lotgi1|207643 | A  B  C | 3  -  - | 1.4 |  | IC |
| Similar to chitobiase | Lotgi1|209107 | A^2^  B^2^  C^2^ | 14  12  13 | 9.0  9.0  10.5 | si/tm | IC (Lyso) |
| Similar to thioester-containing protein; α2-macroglobulin family | Lotgi1|209261 | A^2^  B^2^  C^2^ | 58  53  56 | 15.9  12.4  14.8 |  | EC |
| Similar to α-N-acetylgalactosaminidase | Lotgi1|209340 | A^2^  B^2^  C^2^ | 5  10  10 | 1.4  3.6  6.7 | si | IC (Lyso) |
| Similar to ribosomal protein S14 | Lotgi1|209986 | A  B  C | 3  -  - | 3.0  - |  | IC |
| Similar to GTP-binding SAR1B | Lotgi1|210455 | A  B  C | 3  -  - | 0.9 | si | EC |
| Similar to mitochondrial ATP synthase B | Lotgi1|210633 | A  B  C | 3  -  - | 0.5 | si | EC |
| Similar thioester-containing protein; α2-macroglobulin family | Lotgi1|211452 | A^2^  B  C | 8  4  7 | 0.8  0.3  0.6 |  | EC |
| Similar to proteasome subunit α | Lotgi1|212487 | A  B  C | 4  2  2 | 1.2  0.5  0.5 |  | IC |
| Similar to phosphoglycerate kinase | Lotgi1|212640 | A  B  C | 8  5  - | 1.0  0.6 |  | IC |
| Similar to peptidyl-prolyl cis/trans isomerase B | Lotgi1|212757 | A  B  C | 6  7  6 | 2.5  3.3  2.5 | si | E*C* |
| Similar to Na^+^/K^+^ ATPase, β-subunit | Lotgi1|213719 | A  B  C | 4  4  3 | 1.5  1.5  1.0 | tm | IC (Mito) |
| Similar to 4-hydroxyphenylpyruvate dioxygenase/ glyoxalase | Lotgi1|214078 | A  B  C | 4  -  - | 0.6 |  | IC |
| Similar to cytb5 reductase | Lotgi1|214125 | A  B  C | 4  -  - | 2.2 | si/tm | IC (ER) |
| Similar to MEC2; domains: band_7, stomatin; shares peptides with Lotgi1|231818 | Lotgi1|214616 | A  B  C | 2  3  2 | 0.3  0.5  0.3 | tm | M |
| Similar to gelsolin; shares 1 peptide with Lotgi1|105757 | Lotgi1|214936 | A  B  C | 7  5  4 | 2.6  0.9  0.7 |  | IC |
| Similar to mollusk-like growth factor; domain: Adenosine deaminase-related growth factor | Lotgi1|215090 | A  B  C | 5  -  3 | 0.6  0.3 | si/tm | EC |
| Similar to nuclear transport factor-2; domain: NTF2 | Lotgi1|215219 | A  B  C | 2  -  - | 3.6 |  | IC |
| Similar to non-muscle myosin light chain | Lotgi1|215828 | A^2^  B  C | 2  -  - | 0.8 |  | IC |
| Similar to CDGSH iron-sulfur domain-containing protein 2 | Lotgi1|216266 | A  B  C | 3  -  - | 1.7 | tm | IC (Mito membrane) |
| 78kDa glucose-regulated protein/HSP70; shares 1 peptide with Lotgi1|177837 | Lotgi1|216416 | A  B  C^2^ | 8  4  3 | 0.8  0.3  0.2 | si | IC (ER, lumen) |
| Similar to si:dkey-222f8.3/endoribonuclease; domain: XendoU | Lotgi1|216792 | A  B  C | 5  4  4 | 1.4  1.4  1.0 |  | IC (Nuc) |
| Similar to ribosomal protein L12 | Lotgi1|217320 | A^2^  B^2^  C^2^ | 6  5  5 | 4.6  3.2  3.2 |  | IC |
| Fructose-bisphosphate aldolase | Lotgi1|217342 | A^2^  B  C | 11  6  5 | 4.1  1.7  1.0 |  | IC |
| Similar to ribosomal protein L9 | Lotgi1|217766 | A  B  C | 5  4  4 | 2.6  1.8  1.8 |  | IC |
| Similar to α-fucosidase | Lotgi1|217885 | A  B  C | 2  3  3 | 0.3  0.4  0.4 | si/tm, tm | EC/TM |
| Similar to MAP kinase | Lotgi1|218948 | A  B  C | -  -  4 | 0.9 |  | IC |
| Similar to Rab GDP dissociation inhibitor | Lotgi1|218952 | A^2^  B  C | 12  10  6 | 3.0  2.2  1.0 |  | IC |
| Similar to Mago nashi; N-term: acetyl-Ala | Lotgi1|219308 | A  B  C | 2  -  - | 0.7 |  | IC |
| Similar to annexin, type IV | Lotgi1|219490 | A^2^  B  C | 5  -  - | 1.0 |  | IC |
| Similar to annexin, type IV | Lotgi1|219492 | A^2^  B  C | 10  6  4 | 3.0  1.8  0.6 |  | IC |
| Similar to actin-related 2/3 complex subunit 2 | Lotgi1|220774 | A  B  C | 4  -  - | 0.8 |  | IC |
| Similar to isocitrate dehydrogenase | Lotgi1|220933 | A  B  C | 3  -  - | 0.3 |  | IC |
| Similar to cathepsin L-like cysteine proteinase | Lotgi1|221163 | A  B  C | 2  2  3 | 0.3  0.3  0.4 | si/tm | IC (Lyso) |
| Similar to proactivator polypeptide (prosaposin) | Lotgi1|221240 | A^2^  B^2^  C^2^ | 14  7  14 | 2.9  0.7  2.9 | si | EC |
| Similar to dipeptidyl peptidase 1/cathepsin C | Lotgi1|221343 | A  B  C | -  3  2 | 0.4  0.3 |  | IC (Lyso) |
| Similar to Rab 5 | Lotgi1|222012 | A  B  C | -  3  2 | 1.5  0.6 |  | IC |
| Similar to saccharopine dehydrogenase b | Lotgi1|222130 | A  B  C | 4  -  - | 0.6 | tm | IC (Mito) |
| Similar to 14-3-3 ζ protein; shares 1 peptide with Lotgi1|205379 and 145856 | Lotgi1|222442 | A^2^  B  C | 8  8  7 | 2.4  2.4  1.9 |  | IC |
| GAPDH; shares 1 peptide with contaminant (bovine GAPDH) | Lotgi1|222542 | A^2^  B  C | 12  9  9 | 9.0  3.6  4.3 |  | IC |
| Similar to peptidyl-prolys cis/trans isomerase | Lotgi1|222979 | A^2^  B^2^  C^2^ | 5  5  4 | 2.4  12.9  9.0 | si | IC (ER, lumen) |
| Similar to UTP-glucose-1-phosphate uridyltransferase | Lotgi1|223337 | A  B  C | 5  -  - | 0.5 |  | IC |
| Similar to Rab 7 | Lotgi1|223383 | A  B  C | 5  3  3 | 1.3  0.6  0.6 | si | IC (vesicles) |
| Similar to α-tubulin | Lotgi1|223558  Lotgi1|237161  Lotgi1|203680  Lotgi1|213577 | A^2^  B^2^  C^2^ | 14  11  5 | 5.4  2.4  0.9 |  | IC |
| Similar to peptidyl-prolyl cis/trans isomerase; N-term: acetyl-Ala | Lotgi1|223760  Lotgi1|198520 | A  B  C | 4  3  - | 1.8  1.8 |  | IC |
| Similar to DJ-1 protein; domain: GATase1(glutamine amidotransferase)_DJ-1 | Lotgi1|223821 | A  B  C | 4  -  - | 1.0 |  | IC |
| Similar to ribosomal protein S3 | Lotgi1|223917 | A  B  C | 4  2  - | 1.0  0.4 |  | IC |
| Similar to ribosomal protein L5 | Lotgi1|224562 | A^2^  B  C | 4  2  - | 1.3  0.5 |  | IC |
| Similar to proline iminopeptidase | Lotgi1|224583 | A  B  C | 3  4  - | 0.4  0.5 |  | IC |
| Similar to Rab11 | Lotgi1|225073 | A  B  C | 5  4  5 | 0.5  1.3  1.8 |  | IC |
| Similar to malate dehydrogenase | Lotgi1|225558 | A^2^  B  C | 7  5  - | 1.2  0.8 |  | IC |
| Similar to peroxiredoxin 6 | Lotgi1|225601 | A^2^  B  C | 4  -  - | 0.9 |  | IC |
| Similar to epidymal secretory protein E1-like/ Niemann-Pick C2 protein ; 15% Val, 11% Ser, 10% Lys | Lotgi1|226175 | A^2^  B  C^2^ | 4  4  4 | 2.7  4.2  4.2 | si/tm | EC |
| Similar to guanine nucleotide-binding protein Gi α; shares 1 peptide with Lotgi1|163596 | Lotgi1|226384 | A  B  C | 4  -  - | 1.3 |  | IC |
| Uncharacterized protein; domain: chitin_binding_3 | Lotgi1|226726 | A^2^  B^2^  C^2^ | 3  4  5 | 0.8  1.2  1.6 | si/tm | EC/TM |
| Similar to small G protein/Ras-like protein | Lotgi1|226838 | A  B  C | 4  -  4 | 1.8  1.8 |  | IC |
| Similar to villin-1/gelsolin | Lotgi1|227360 | A^2^  B  C | 5  4  5 | 0.7  0.6  0.7 |  | IC |
| Uncharacterized protein; 10% Pro, 11% Leu; aa17-126: 17% R+K, 12% P, 11% L; pI: 11 | Lotgi1|227783 | A^2^  B^2^  C^2^ | 4  3  4 | 5.8  2.2  3.6 | si | EC |
| Uncharacterized protein; 27% Thr; pI: 4.3; aa20-300: 42% T, 15% E; aa301-482: 16% R | Lotgi1|227996 | A^2^  B^2^  C^2^ | 5  2  2 | 6.5  0.8  2.2 | si, tm | EC/TM |
| Similar to Pif97/BMSP ^1^; domains: vWA, chitin-binding | Lotgi1|228264 | A^2^  B^2^  C^2^ | 18  26  13 | 9.0  71.0  4.6 | si | EC |
| Uncharacterized protein; 12% Pro, 10% Tyr | Lotgi1|228268 | A^2^  B^2^  C^2^ | 7  7  7 | 5.6  9.0  5.6 | si/tm | EC |
| Uncharacterized protein; 18% Arg, 11% Ser; pI 11.7; R/H/S-rich from aa103-150 (30% R, 12% H, 12% S) | Lotgi1|228385 | A^2^  B^2^  C^2^ | 3  -  - | 3.0 | si/tm | EC |
| Similar to cytosolic fatty acid-binding protein | Lotgi1|228443 | A  B  C | 2  -  - | 0.9 |  | IC |
| Uncharacterized protein; domains: tetraspanin | Lotgi1|228639 | A  B  C | 2  2  2 | 0.7  0.7  0.7 | tm | M |
| Uncharacterized protein; domains: Cys-rich repeat; 12% Gln, 13% Pro | Lotgi1|228882 | A^2^  B^2^  C^2^ | 18  17  20 | 7.3  7.3  15.2 | si/tm | EC |
| Similar to thioredoxin/nucleoredoxin | Lotgi1|229002 | A  B  C | -  -  2 | 0.6 |  | IC |
| Similar to perlucin-like protein ^1^ (aa1-156);  aa200-470: ZP_2 domain | Lotgi1|229175 | A^2^  B^2^  C^2^ | 22  18  20 | 26.8  17.2  18.8 | si | EC |
| Uncharacterized protein; domains: cadherin repeats | Lotgi1|229248 | A  B  C | 5  7  3 | 0.1  0.2  0.1 | si, tm | TM |
| Uncharacterized protein; domains: cadherin repeats | Lotgi1|229249 | A  B  C | 6  5  - | 0.2  0.1 | si/tm, tm | TM |
| Similar to proteasome subunit α-type | Lotgi1|229407 | A  B  C | 6  6  5 | 1.9  1.9  1.4 |  | IC |
| Uncharacterized protein; domain: carbohydrate –binding CBM_6/Galactose-binding; 11% Ser | Lotgi1|229427 | A^2^  B^2^  C^2^ | 13  4  8 | 3.3  0.5  1.3 | si | EC |
| Uncharacterized protein | Lotgi1|229482 | A^2^  B^2^  C^2^ | 3  5  2 | 0.7  1.9  - | si | EC |
| Uncharacterized protein; domain: PMP-22/EMP/MP20/claudin | Lotgi1|229513 | A  B  C | 2  3  3 | 0.7  1.8  1.8 | si/tm, tm | TM |
| Uncharacterized protein; domain: SOUL/heme-binding | Lotgi1|229543 | A^2^  B^2^  C^2^ | 8  9  9 | 30.6  30.6  38.8 | si | EC |
| Uncharacterized protein; domains: Peptidase C26/γ-glutamyl hydrolase | Lotgi1|229608 | A  B  C | 3  4  3 | 0.9  1.3  0.9 | si, tm | EC |
| Similar to thioester-containing protein/CD109 antigen-like;domains: A2M_N, A2M_N_2 | Lotgi1|229818 | A^2^  B^2^  C^2^ | 15  12  15 | 2.0  1.6  2.2 | si/tm, tm | TM |
| Similar to ribosomal protein L14 | Lotgi1|229862 | A  B  C | 3  2  3 | 2.2  1.2  2.2 |  | IC |
| Similar to Rab6 | Lotgi1|230007 | A  B  C | 3  6  2 | 0.7  1.9  0.4 |  | IC |
| Uncharacterized protein; 15% Lys, 12% Leu; 3 ~75aa repeats | Lotgi1|230068 | A  B  C | 7  13  5 | 2.2  10.5  1.7 | si/tm, tm | TM |
| Similar to ribosomal protein L10a | Lotgi1|230263 | A  B  C | 4  -  - | 1.8 |  | IC |
| Similar to Ras-related Rac | Lotgi1|230301 | A  B  C | 3  -  - | 2.2 |  | IC |
| Similar to calcineurin; domain: EFh; D-rich motif (aa152-171) | Lotgi1|230492 | A^2^  B^2^  C^2^ | 5  6  6 | 6.2  6.2  9.0 | si | EC |
| Similar to calcium-binding protein; domain: EFh | Lotgi1|230493 | A^2^  B^2^  C^2^ | 3  2  - | 1.2  0.5 | si | EC |
| Uncharacterized protein; 16% Pro | Lotgi1|230510 | A^2^  B^2^  C^2^ | 3  5  5 | 1.8  3.6  2.6 |  | ? |
| Uncharacterized protein/similar to cerebellin; domain: C1q/TNF_like | Lotgi1|230589 | A  B  C | -  -  3 | 1.7 | si | EC |
| Similar to vitellogenin | Lotgi1|230838 | A  B  C | -  -  6 | 0.1 | si | EC |
| Similar to pacifastin; domains: VWC/pacifastin | Lotgi1|230854  Lotgi1|176463 | A^2^  B^2^  C^2^ | 4  3  3 | 5.8  5.8  5.8 |  | EC |
| Uncharacterized protein | Lotgi1|230881 | A^2^  B^2^  C^2^ | 2  -  - | 0.3 | si, tm | EC/TM |
| Similar to UP2 ^1^; 12% Leu, 10% Ala | Lotgi1|231009 | A^2^  B^2^  C^2^ | 5  5  4 | 38.8  38.8  9.0 | si/tm | EC |
| Uncharacterized protein | Lotgi1|231046 | A^2^  B^2^  C^2^ | 7  8  6 | 9.0  15.7  9.0 | si | EC |
| Uncharacterized protein; domains: 2 x chitin-binding peritrophin-A; some similarity to PIF/BMSP ^1^ | Lotgi1|231395 | A^2^  B^2^  C^2^ | 6  5  7 | 15.7  5.0  15.7 | si | EC |
| Uncharacterized protein; domain: EFh, shares peptides with Lotgi1|231427; 13% Val, pI: 5.0 | Lotgi1|231426 | A^2^  B^2^  C^2^ | -  2  - | 1.2 | tm | TM |
| Uncharacterized protein; domain: EFh; shares peptides with Lotgi1|231426; 16% Val; pI:4.8 | Lotgi1|231427 | A  B  C^2^ | -  2  - | 2.2 | si/tm | EC/TM |
| Similar to predicted matrix metalloproteinase; domains: ZnMc_MMP, HX, PG (peptidoglycan-binding) | Lotgi1|231475 | A^2^  B  C | -  -  2 | 0.2 | si | EC |
| Similar to ADP-ribosylation factor 6; domains: small GTPase ARF/SAR type/Rab type | Lotgi1|231498 | A  B  C | 4  -  2 | 3.6  1.2 | si | EC |
| Similar to protein kinase; domains: ERK1/2 MAP kinase | Lotgi1|231498 | A  B  C | 4  -  - | 0.7 |  | IC |
| Similar to macrophage migration inhibitory factor | Lotgi1|231585 | A  B  C | 2  -  - | 1.5 |  | EC |
| Similar to cytoplasmic aspartate aminotransferase I | Lotgi1|231665 | A  B  C | 6  -  - | 0.8 |  | IC |
| Similar to actin-binding protein; domains:cofilin/tropomyosin type | Lotgi1|231819 | A  B  C | -  2  3 | 0.3  0.7 |  | IC |
| Uncharacterized protein; domain: SEA; 27% Ser, 14% Thr | Lotgi1|231862 | A^2^  B  C | -  3  4 | 0.7  1.4 | tm | TM |
| Similar to cyt f | Lotgi1|231865 | A  B  C | 3  3  3 | 1.7  4.2  4.2 | si/tm | IC (Mito) |
| Uncharacterized protein; domains: chitin-binding peritrophin A; Pro-rich extensin-like; aa470-600: 29% pro, 16% Thr, 12% Gln, 12% Asn | Lotgi1|231869 | A^2^  B^2^  C^2^ | 29  25  30 | 5.4  3.7  5.3 |  | EC |
| Similar to Pif/BMSP ^1^; domains: vWA, chitin-binding | Lotgi1|232022 | A^2^  B  C^2^ | 5  5  6 | 0.5  0.5  0.6 |  | EC |
| Similar to proteasome subunit α | Lotgi1|232029 | A  B  C | 4  4  - | 1.2  1.2 |  | IC |
| Similar to ADP,ATP carrier protein | Lotgi1|232343 | A  B  C | 5  4  - | 0.9  0.7 | tm | IC (Mito, membrane) |
| Similar to proteasome subunit α-type | Lotgi1|232677 | A  B  C | 3  -  - | 0.5 |  | IC |
| Uncharacterized protein; 11% Ser | Lotgi1|232714 | A^2^  B^2^  C^2^ | 17  15  19 | 2.7  1.6  2.5 | si, tm | TM |
| Uncharacterized protein; domains: SEA, chitin-binding peritrophin A | Lotgi1|232880 | A  B  C | 15  29  11 | 0.5  1.4  0.4 | si/tm | EC |
| Similar to quinone oxidoreductase; domains: alcohol dehydrogenase (ADH_Zn_N)/GroES-like | Lotgi1|232898 | A  B  C | 2  -  - | 0.3 |  | IC |
| Uncharacterized protein; domains: CAP/VSTPX_like /protease_inhibitor_16 | Lotgi1|233199 | A^2^  B^2^  C^2^ | 20  21  23 | 132.4  364.2  272.8 | si | EC |
| Uncharacterized protein; domains: CAP/VSTPX_like/  protease_inhibitor_16 | Lotgi1|233200 | A^2^  B^2^  C^2^ | 26  25  29 | 665.1  507.0  2954.2 | si | EC |
| Uncharacterized protein; domains: CAP/allergen V5 | Lotgi1|233201 | A^2^  B^2^  C^2^ | -  6  6 | 1.0  0.8 | si | EC |
| Uncharacterized protein | Lotgi1|233348 | A^2^  B^2^  C^2^ | 11  10  12 | 2.7  1.7  3.4 | si/tm, tm | TM |
| Uncharacterized protein; domain: collagen triple helix repeat; 19% Gly, 13% Ala, 11% Val | Lotgi1|233390 | A  B  C | -  -  2 | 3.6 | si/tm | EC/TM |
| Uncharacterized protein; A/P-rich motif aa150-170; H-rich motif aa171-185 | Lotgi1|233397 | A^2^  B^2^  C^2^ | 6  6  6 | 36.3  12.9  50.8 | si/tm | EC/TM |
| Similar to cofilin/actin-depolymerizing factor | Lotgi1|233408 | A^2^  B^2^  C^2^ | 8  5  6 | 9.0  3.3  4.3 |  | IC |
| Uncharacterized protein; 31% Asp, D+E=41%, pI 3.6;  Similar to very acidic proteins like aspein | Lotgi1|233420 | A^2^  B^2^  C^2^ | 16  14  15 | >10,000  >10,000  >10,000 | si/tm | EC |
| Uncharacterized protein; 17% Ala | Lotgi1|233451 | A^2^  B^2^  C^2^ | 2  2  2 | 20.5  3.6  9.0 | si/tm, tm | TM |
| Uncharacterized protein; 14% Gly, 13% Asn, 11%Arg, 11% Asp; similar to nacrein B3/B4 ^1^ | Lotgi1|233461 | A^2^  B^2^  C^2^ | 5  10  6 | 0.7  2.9  1.3 |  | EC |
| Uncharacterized protein | Lotgi1|233524 | A  B  C | 2  -  - | 0.6 | si, tm | TM |
| Similar to ependymin-related protein 1/X-box-binding protein; domains: ependymin | Lotgi1|233583 | A^2^  B  C | 6  5  7 | 2.2  1.8  2.8 | si | EC |
| Similar to glutathione-S-transferase µ | Lotgi1|233779 | A^2^  B  C | 10  7  8 | 20.5  5.0  6.7 | si | IC |
| Uncharacterized protein; 10% Lys | Lotgi1|233820 | A^2^  B  C | 3  -  3 | 0.5  0.5 |  | IC |
| Similar to F-actin-capping protein | Lotgi1|234041 | A  B  C | 3  -  2 | 0.6  1.3 |  | IC |
| Uncharacterized protein; domain: CD225 (interferon-induced transmembrane protein), 10% Gln, Gln-rich N-term (~aa20-50, 4x P[Q]_2-3_GY) | Lotgi1|234302 | A  B  C | 3  3  3 | 30.6  30.6  30.6 | tm | TM/M |
| Uncharacterized protein; 13% Ala, 11% Gly | Lotgi1|234386 | A^2^  B^2^  C^2^ | 2  4  3 | 1.8  3.6  2.6 | si/tm | EC/TM |
| Uncharacterized protein | Lotgi1|234387 | A^2^  B^2^  C^2^ | 13  14  14 | 27.9  69.2  82.8 |  | ? |
| Similar to D-lactate dehydrogenase | Lotgi1|234402 | A  B  C | 3  -  - | 0.6 |  | IC |
| Uncharacterized protein; domains: chitin-binding peritrophin A | Lotgi1|234405 | A^2^  B^2^  C^2^ | 2  8  14 | 0.4  3.1  19.3 | si | EC |
| Uncharacterized protein; domains: multiple FN3; shares 1 peptide with Lotgi1|234471 | Lotgi1|234472 | A^2^  B^2^  C^2^ | 3  -  - | 0.1 |  | ? |
| Similar to tyrosinase | Lotgi1|234481 | A  B  C | 2  -  5 | 0.2  0.6 |  | ? |
| Uncharacterized protein; 13% Cys, 10% Asn, 10% Gly; 34% identity to E4W3F5_HALDV, B3TK45_HALDV, B3TK76_HALDV | Lotgi1|234488 | A^2^  B^2^  C^2^ | 5  5  6 | 4.6  4.6  6.5 | si | EC |
| Similar to stanniocalcin | Lotgi1|234508 | A  B  C | 2  -  6 | 0.8  6.5 | si | EC |
| Uncharacterized protein; 18% Asp, 16% Arg, 14% Gly; many GDDR and related repeats; domain: superoxide dismutase; very similar to acidic shell proteins such as aspein ^1^, DGRP_HALAI ^1^, or the aragonite-binding domain of Pif ^1^ | Lotgi1|234845 | A^2^  B^2^  C^2^ | 2  -  6 | 0.6  1.9 | si | EC |
| Similar to melanotransferrin; domains: transferrin | Lotgi1|234865 | A^2^  B^2^  C^2^ | 22  19  21 | 2.2  1.9  2.0 | si | EC |
| Uncharacterized protein; domain: Sushi; 19% Gln, 11% Pro; 42% Q in aa281-630; G/L/A-rich region aa631-928; protein different from Lotgi1|234884 retrieved from annotation homepage! | Lotgi1|234884 | A^2^  B^2^  C^2^ | 4  6  6 | 1.5  5.3  4.0 | si | EC |
| Uncharacterized protein; 10% Gly | Lotgi1|234885 | A^2^  B^2^  C^2^ | 5  5  6 | 99.0  30.6  999.0 | si/tm | EC |
| Uncharacterized protein; domain: otoanchorin (aa~900-1100); 12% Asp, 12% Leu, pI 4.7 | Lotgi1|235120 | A^2^  B^2^  C^2^ | 39  35  42 | 29.7  17.0  78.0 |  | ? |
| Uncharacterized protein; 11% Ala, 13% Pro; aa120-247: 20% P, 16% A, 10% Q | Lotgi1|235497 | A^2^  B^2^  C^2^ | 7  12  12 | 34.9  463.2  598.5 | si/tm, tm | EC/TM |
| Similar to gigasin-2 ^1^; domains: EGF, ZP_2 | Lotgi1|235548 | A^2^  B^2^  C^2^ | 13  13  11 | 7.8  9.0  5.8 | si, tm | EC |
| Similar to mannose receptor; domains: EGF, CLECT, ZP_2 | Lotgi1|235549 | A^2^  B^2^  C^2^ | 20  19  19 | 2.2  1.0  1.4 | si/tm, tm | EC/TM |
| Uncharacterized protein; 11% Arg, 12% Thr, 10% Ser | Lotgi1|235609 | A^2^  B^2^  C^2^ | 4  7  4 | 1.2  3.0  1.2 | si | EC |
| Uncharacterized protein; 15% Pro, 15% Thr | Lotgi1|235610 | A^2^  B^2^  C^2^ | 8  9  10 | 22.7  22.7  99.0 | si | EC |
| Uncharacterized protein; domain: annexin A7; 14% Gly, 13% Thr; 16 x [GGQPs/tT]; 44% identity to mpn88 in a ~100aa overlap | Lotgi1|235621 | A^2^  B^2^  C^2^ | 7  6  9 | 25.8  25.8  99.0 | si | EC |
| Uncharacterized protein/similar to UDP-N-acetyl-a-D-galactosamine:polypeptide N-acetylgalactosaminyl-transferase | Lotgi1|235690 | A  B  C | 6  10  9 | 0.7  1.3  1.2 | si/tm | IC (Golgi, membrane) |
| Similar to FGFBP; domain: FGFBP_1 | Lotgi1|235694 | A^2^  B^2^  C^2^ | 2  -  3 | 0.7  1.8 | si/tm | EC |
| Uncharacterized protein; 11% Ser | Lotgi1|235865 | A^2^  B  C | 5  4  5 | 2.6  1.8  1.8 | si | EC |
| Similar to ATP synthase γ-chain | Lotgi1|235900 | A  B  C | 3  -  - | 0.5 |  | IC (Mito) |
| Uncharacterized protein; 15% Ala | Lotgi1|235969 | A^2^  B  C | 3  2  4 | 55.2  4.6  55.2 | tm | TM |
| Uncharacterized protein; domain: hedgehog/DD-peptidase | Lotgi1|235988 | A^2^  B^2^  C^2^ | 27  23  20 | 4.7  3.0  2.6 | si | EC |
| Uncharacterized protein; 13% Tyr (Y+F=18%) | Lotgi1|236183 | A^2^  B^2^  C^2^ | 9  10  10 | 50.8  137.9  137.9 | si | EC |
| Similar to ADP-ribosylation factor; shares peptides with Lotgi1|228722 | Lotgi1|236204 | A  B  C | 3  2  - | 1.2  0.7 |  | IC |
| Similar to eukaryotic initiation factor 5A | Lotgi1|236304 | A^2^  B  C^2^ | 2  -  - | 0.7 |  | IC |
| Similar to guanine nucleotide-binding protein subunit β; N-term acetyl-Ser | Lotgi1|236462 | A  B  C | 8  4  6 | 3.9  1.0  1.9 |  | IC |
| Uncharacterized protein; 19% Pro, 10% Ala, 10% Arg, 10% Val | Lotgi1|236689 | A^2^  B^2^  C^2^ | 7  11  10 | 2.2  5.1  3.4 | si | EC |
| Uncharacterized protein; 22% Gln, 19% Pro; aa268-356: 4 x [xAQPGAYQQP(x)_2-4_ GAYxQQP] | Lotgi1|236690 | A^2^  B^2^  C^2^ | 2  2  2 | 2.2  2.2  2.2 | si | EC |
| Uncharacterized protein; 22% Pro, 13% Gln, 10% Ala; Q-rich regions: ~aa61-160 and ~aa721-990; P-rich: ~aa280-600 and ~780-970 | Lotgi1|236691 | A^2^  B^2^  C^2^ | 2  -  3 | 0.4  0.4 |  | ? |
| Uncharacterized protein;domain: ADAM_MEPRO | Lotgi1|236770 | A^2^  B^2^  C^2^ | 34  38  31 | 23.1  37.7  23.1 | si | EC |
| Similar to ribosomal protein L30 | Lotgi1|236815 | A^2^  B  C^2^ | 2  -  - | 1.2 |  | IC |
| Uncharacterized protein; domains: EGF, Vitellinogen, FN3; shares peptides with Lotgi1|173550 | Lotgi1|236952 | A^2^  B^2^  C^2^ | 76  67  39 | 0.7  0.6  0.3 | si | EC |
| Similar to mannan endo-1,4-β-mannosidase; domains: Glycoside hydrolase, family 26 | Lotgi1|236955 | A^2^  B^2^  C | 3  3  4 | 0.8  0.8  1.6 | si | EC |
| Uncharacterized protein; domain: LamG/concanavalin A-like lectin | Lotgi1|237103 | A^2^  B  C^2^ | 3  4  - | 0.9  1.8 | si | EC |
| Similar to gastric intrinsic factor/transcobalamin; shares peptides with Lotgi1|237142 | Lotgi1|237143 | A^2^  B^2^  C^2^ | 5  4  4 | 62.1  24.1  62.1 | si | EC |
| Uncharacterized protein; domain: reelin; 21% Thr, 15% Ser, 11% Pro; aa190-300: 51% Thr | Lotgi1|237152 | A^2^  B^2^  C^2^ | 2  -  3 | 0.5  1.2 | si/tm | EC |
| Similar to Pi-class glutathione S-transferase | Lotgi1|237303 | A  B  C | 3  -  - | 0.8 |  | IC |
| Similar to prohibitin | Lotgi1|237446 | A  B  C | 5  3  - | 1.3  0.6 | si | EC |
| Similar to chitin-binding protein P86860 ^1^ | Lotgi1|237510 | A^2^  B^2^  C^2^ | 8  10  11 | 30.6  41.2  74.0 |  | EC |
| Similar to acidic ribosomal protein P0 | Lotgi1|237709 | A  B  C | 7  7  5 | 2.0  1.6  1.3 |  | IC |
| Uncharacterized protein | Lotgi1|237778 | A  B  C | -  4  2 | 2.2  0.8 | si | EC |
| Uncharacterized protein; 14% Pro,12% Gly | Lotgi1|237996 | A^2^  B^2^  C^2^ | -  2  2 | 99.0  99.0  99.0 | si, tm | EC/TM |
| Similar to nacrein-like protein ^1^; domain: α-carbonic anhydrase | Lotgi1|238082 | A^2^  B^2^  C^2^ | 35  30  37 | 8375.8  8375.8  >10,000 | si/tm | EC |
| Uncharacterized protein; domain: DUF3421 | Lotgi1|238094 | A^2^  B^2^  C^2^ | 6  8  6 | 4.0  19.0  4.0 | si | EC |
| Similar to triosephosphate isomerase | Lotgi1|238326 | A^2^  B  C | 12  9  7 | 6.0  6.0  3.1 |  | IC |
| Uncharacterized protein; 19% Asp, 11%Val, 10% Asn, 10% Ala; D+E=25%; pI 3.6; some similarity to aspein ^1^ | Lotgi1|238358 | A^2^  B^2^  C^2^ | 11  9  10 | 236.1  55.2  236.1 | tm | TM |
| Uncharacterized protein; domains: vWA_ECM, chitin-binding CBM_14 (peritrophin A) | Lotgi1|238400 | A^2^  B^2^  C^2^ | 9  10  11 | 25.1  55.2  55.2 | si | EC |
| Uncharacterized protein | Lotgi1|238415 | A^2^  B^2^  C^2^ | 4  2  4 | 0.6  0.3  0.6 | si/tm | EC/TM |
| Similar to protein Mo25 | Lotgi1|238430 | A  B  C | 6  5  4 | 1.1  0.8  0.6 |  | IC |
| Similar to BMSP100 (aragonite-binding)^1^; 18% Gly, 12% Ser, 10% Thr; | Lotgi1|238526 | A^2^  B^2^  C^2^ | 56  56  60 | 1063.2  3261.2  1204.3 |  | EC |
| Similar to aminopeptidase N; domain: peptidase M1_APN_2 | Lotgi1|238560 | A^2^  B^2^  C^2^ | 31  28  25 | 10.2  7.4  7.0 | si | EC |
| Uncharacterized protein; 12% Lys, 11% Val | Lotgi1|238760 | A^2^  B  C^2^ | 3  -  - | 1.2 | si | EC |
| Uncharacterized protein; 13% Ala, 11% Arg, 11% Leu; KRA-rich C-terminus (aa185-219) | Lotgi1|238831 | A^2^  B^2^  C^2^ | 9  9  10 | 27.5  34.1  34.1 | si | EC |
| Similar to chloride-channel Ca-activated-2-like; domains: vWA, Chloride channel, DUF1973 | Lotgi1|238844 | A  B  C | 7  11  3 | 0.5  0.8  0.2 | si, tm | TM/M |
| Similar to perlustrin ^1^; domain: IGFBP_N_2 | Lotgi1|238970 | A^2^  B^2^  C^2^ | 3  3  - | 9.0  9.0  3.6 | si | EC |
| Uncharacterized protein; 14% Ser, 10% Gly ; 93% identical | Lotgi1|239005  Lotgi1|239006 | A^2^  B^2^  C^2^ | 4  5  6 | 12.9  25.8  25.8 | si | EC |
| Uncharacterized protein; domains: antistasin, WAP | Lotgi1|239125 | A^2^  B^2^  C^2^ | 76  71  81 | 179.2  144.5  497.5 | si | EC |
| Uncharacterized protein; 10% Lys | Lotgi1|239159 | A^2^  B^2^  C^2^ | 8  9  5 | 30.6  55.2  12.3 | si/tm | EC/TM |
| Uncharacterized protein; 16% Gly, 12% Met, 10% Gln; Gly-rich motif aa30-65 | Lotgi1|239170 | A^2^  B^2^  C^2^ | 5  5  5 | 176.8  561.3  3161.3 | si/tm, tm | TM |
| Uncharacterized protein; 20% Gly, 18% Met, 12% Ala, 10% Leu; some similarity to shematrins ^1^ | Lotgi1|239174 | A^2^  B^2^  C^2^ | 5  5  6 | 71.0  71.0  137.9 | si/tm, tm | M |
| aa1-420: similar to nacrein ^1^; domain: carbonic anhydrase  aa421-633: 26% Asp, 23% Gly, 22% Arg, 13% Asn; pI:4.8; similar to aspein ^1^ | Lotgi1|239188 | A^2^  B^2^  C^2^ | 14  15  16 | 35.5  47.7  99.0 | si | EC |
| Similar to alginate lyase | Lotgi1|239189 | A^2^  B  C | 9  9  8 | 24.1  24.1  30.6 | si/tm | EC/TM |
| Uncharacterized protein ; 12% Pro, 11% Val, 10% Pro | Lotgi1|239628  Lotgi1|239234 | A  B  C | 6  -  - | 0.7 |  | ? |
| Similar to elongation factor 1α | Lotgi1|239271 | A^2^  B  C^2^ | 3  3  3 | 0.6  0.6  0.6 |  | IC |
| Uncharacterized protein; 10% Pro, 12% Ser, 13% Thr; Thr-rich motif from ~aa185-240 | Lotgi1|239339 | A^2^  B^2^  C^2^ | 8  7  10 | 1.3  1.1  2.2 |  | ? |
| Uncharacterized protein; 22% Gly, 12% Asn; aa30-105: 49% Gly, 26% Asn; some similarity to GAAP_HALAI ^1^ | Lotgi1|239447 | A^2^  B^2^  C^2^ | 5  5  5 | 14.8  24.1  14.8 |  | ? |
| Uncharacterized protein; domains: IG-like | Lotgi1|239573 | A  B  C | -  2  - | 0.3 | si/tm | EC/TM |
| Similar to Pif/BMSP ^1^; domains: chitin_binding CBM_14/ peritrophin A; Thr-rich motif from aa300-372 | Lotgi1|239574 | A^2^  B^2^  C^2^ | 40  35  39 | 105.6  30.6  42.5 | si | EC |
| Uncharacterized protein | Lotgi1|239621 | A  B  C | -  2  - | 1.0 |  | ? |
| Similar to sulfate transporter/prestin_like; domains: Sulphate transporter, STAS | Lotgi1|53031 | A^2^  B  C | 3  -  - | 0.6 | tm | M |
| Uncharacterized protein/similar to stromal cell-derived factor 2-like; domain: reeler | Lotgi1|66392 | A  B  C | 3  -  - | 5.3 |  | EC |
| Similar to probable thiopurine S-methyltransferase; domain: TPMT | Lotgi1|67729 | A  B  C | 2  -  - | 0.5 |  | IC |
| Uncharacterized protein; domains: scavenger receptor_related | Lotgi1|69892 | A^2^  B^2^  C^2^ | 4  4  4 | 2.2  2.2  2.2 |  | EC/TM |
| Uncharacterized protein; 19% Pro, 15% Ser, 12% Gly; 9 x [g/dSQPGIYP] and 4 x imperfect; some similarity to adhesive plaque matrix protein | Lotgi1|77105 | A^2^  B^2^  C^2^ | 6  7  7 | 397.1  630.0  630.0 |  | EC |
| Similar to trimeric G-protein γ-subunit; domain: partial GGL | Lotgi1|80489 | A  B  C | 2  -  - | 4.6 |  | IC |
| Similar to hephaestin/ceruloplasmin; domain: multicopper oxidase, cupredoxin | Lotgi1|83160 | A^2^  B^2^  C^2^ | 19  17  21 | 22.4  15.2  28.8 |  | TM |
| Similar to pancreatic lipase-related; domain: lipase (fragment) | Lotgi1|98299 | A^2^  B^2^  C | 4  6  6 | 2.2  6.5  9.0 |  | EC |
| Similar to pancreatic lipase-related domain: lipase (fragment) | Lotgi1|98300 | A^2^  B^2^  C^2^ | 6  6  6 | 9.0  9.0  9.0 |  | EC |
| Uncharacterized protein; domain: An_peroxidase/ Peroxidase_3 | Lotgi1|99791 | A^2^  B^2^  C^2^ | 9  6  6 | 1777.3  176.8  99.0 |  | ? |
| Uncharacterized protein; domain: An_peroxidase/ Peroxidase_3 | Lotgi1|99809 | A^2^  B^2^  C^2^ | 3  3  3 | 463.2  463.2  999.0 |  | ? |
| Uncharacterized protein; domain: An_peroxidase/ Peroxidase_3 | Lotgi1|99852 | A^2^  B^2^  C^2^ | 7  6  7 | 999.0  999.0  999.0 |  | ? |

The entries are ordered according to increasing accession numbers, with six digit numbers first, followed by few five digit numbers.  ^1^, previously identified in mollusk shells. ^2^, also identified in acid-soluble matrix. Cleaning of shells before de-mineralization: A, 2h sodium hypochlorite; B, 2h sodium hypochlorite plus 2 x 5min ultrasonication; C, 24h sodium hypochlorite plus 2 x 5min sonication. emPAI was calculated for (group)unique peptides. Si, predicted secretion signal sequence; tm, predicted transmembrane segment(s). EC, extracellular; IC, intracellular; TM, transmembrane; M, membrane; subcellular location according to predicted signal sequences, transmembrane sequences, similarity to other proteins, previous identification in other shell matrices, or Lotgi1 annotation (<http://genome.jgi-psf.org/pages/search-for-genes.jsf?organism=Lotgi1>). The most abundant proteins (average emPAI>1000) are shaded yellow.
