# Supplementary material for: In-depth proteomic analysis of a mollusc shell: acid-soluble and acid-insoluble matrix of the limpet Lottia gigantea
Source: Proteome Sci. 2012 Jun 13;10:28. doi: 10.1186/1477-5956-10-28 (PMC3374290; doi:10.1186/1477-5956-10-28)
Supplement: Additional file 15 — Selected sequence alignments. Doc-file showing sequence alignments of ependymin-related protein, gigasin-2, nacrein-like protein, tyrosinase, UP2, and osteonectin to similar proteins identified in this study. [file 1477-5956-10-28-S15.doc]

**Additional file 15: Selected sequence alignments**

**Figure A**

**Sequence comparison of ependymin-related proteins**

Lotgi1|233583 1 MLKLVVISACLVAVLGQYKFNDESELQMEYPTVAGCCTPDQWEGYQGIIG

B6RB39_HALDI 1 MLSVVLFALIAAAVADP------------------CCTPDQWEGSFGSIT

EPDR1_HALAI 1 MILQAALFLAGLTVVSGSI----------------CCPPKQFNSYQYVTF

EPDR2_HALAI 1 MILQVVLLLACLSGAIVST--------------GACCPPSRFNAFQYVTI

Lotgi1|233583 51 GYARFIKK---GAVYGYTKVSYDAKNERIAAVANMTRDGKNHAIRVIEDY

B6RB39_HALDI 33 GTVTDDMTKTI-KVNGMMA--YDFINKMVAQTTVVTEGSMKMQNKTIINY

EPDR1_HALAI 35 VNSTTTIRALYYIV-------YDGDNQRYLITGDRNNKQLVGTTKVIYDY

EPDR2_HALAI 37 VNSTTRTRGLYYMV-------YDGPNERYLLTGDRLKNLY-GTTRVIYDY

Lotgi1|233583 98 QNGKIYIISLKKNWCKTLTTKRSF---KKACVPKSAVKIGE-FYQGTGL-

B6RB39_HALDI 80 NTKMMYLIDMTRDLCEVKSVDQPM---MQACTPQGATETGT-FYFGAGED

EPDR1_HALAI 78 KKRIAYSIDAKARTCSKFPVQGQFEDQENVCVPGGAEILGPLFY-GYNQ-

EPDR2_HALAI 79 KKGIAYNIDVQKRSCTTFPLHGKFEDQENVCVPRDAVYTGRSAY-GFDQ-

Lotgi1|23358 143 RKLTQVAYYYEHKSFFNVVR-STFDLTKKGCIPT--NEVISGRVKGVNF-

B6RB39_HALDI 126 NMLDATSYKFTVNTM-----GSYLTVTSLDCVPI--TSVTYG-QAGNTAI

EPDR1_HALAI 126 SRLNSQSYAYNTTSLDGSHHNVVTTVSEDDCVPIVICTITTG-GPGGNS-

EPDR2_HALAI 127 GALHSWSYEYNRTHPDGRHQNIETTVTKENCIPIVTTTISTD-ASGGNS-

Lotgi1|233583 189 MDVVGFSDITAGIKDPAVFNPPESCNKTESDSGPNFELHEHMLYREFRY/

B6RB39_HALDI 168 MTSVGFVDIMLKIQDPSVFDPPAACDEAKVTPGENYALHSFFGTPTHHHL

EPDR1_HALAI 174 LYTVGYNDFYPGIKDITVFDIPPYCNA

EPDR2_HALAI 175 LHILGYNDFYPGIRDISMLEIPSYCRA

-----------------------------------------------------------------------------

Peptides sequenced by MS/MS are shown in red. EPDR sequences are from [14], the sequence of B6RB39 was submitted to EMBL/Genebank/DDBJ databases by Kang, DeZoysa and Lee (2006).

**Figure B**

**Sequence comparison of Lottgi1|235548 and gigasin-2 and related EGF-like domain containing protein-1 and -2**

Lotgi1|235548 161 RYYRVLDFLMMHTFLRRLCVVALCLGYIKASADFDCRRTSQS-CVT-GTC

GIGA2_CRAGI 1 MNKMSPLYVLALCCLATTVFAKYDCTNNGGYGCKYGGTC

ELDP1_PINMA 1 MFYLSTFMTIVISLSLVSCSY--------DCNNPGYS-CK—-GTC

ELDP2_PINMA 1 MPPSLSHLFLLSTFASLALCSF--------YCKNPGYP-CLNGGTC

Lotgi1|235548 209 NDVNGDCDCPTDANGVATHRNADCGLEIAKVVPT-ALCGPPCLNGGECYE

GIGA2_CRAGI 40 HFY-GFCICPKGFQ------GEDCGLKTELIS-TAANCTAECKNGGTCYE

ELDP1_PINMA 35 HYY-GPCICNEKLM------GYDCSVLKSRMS-TGSNCTVTCQNNGKCYD

ELDP2_PINMA 38 LY-NGECNCTSGFR------GFNCGLDSSTIS---AACTVECHNKGICFN

Lotgi1|235548 258 PTVGTYMCMCPEAFYGNKCENPRKKVECSGTEITIN-YMPIPTFSGDIFI

GIGA2_CRAGI 82 SD----RCYCPHGFIGDMCEIPDTVARCAPDRMIIEAYRPL-GFVGEVYM

ELDP1_PINMA 77 GS----KCLCSSDYTGDLCEKQTTGARCTLDAVVFEAYRPI-GFVGETYL

ELDP2_PINMA 79 GD----KCYCTKDYMGPTCQQAYDFADCNKSSMKIKAYRPT-EFNGEIFL

Lotgi1|235548 307 LDNRNTPECAFTEANGMYTATFTYQQ---------CGVTTTNDEPNAGDT

GIGA2_CRAGI 127 FQNRKS--CALKQVPSDIRDMMKLERVVLHSDKTECALTRTPDTPKLGDV

ELDP1_PINMA 122 SQSRS---CKLLETTSDVPGMIKFERKIFHGDTSMCGLKKHMDIPSAGDV

ELDP2_PINMA 122 MQSMFG--CKLTEVTSTIPGYKQYELDVPHDSTGPCKLKKTID-ATTGDV

Lotgi1|235548 348 SYEISAAVRFNANIERATDMKLTAKCVIDGTGQSNLNDNIGTVSVDQRTD

GIGA2_CRAGI 175 TMKTTVVSTHNYNQFGPRDSIMDVSCVHTNSFQGSTKEITETAFPFRMVA

ELDP1_PINMA 169 TYEADIYSTFQYNSWGTRDFMDNVKCQYKPTRVGLSMDAPDSLFPIKMSA

ELDP2_PINMA 169 HFEVNVSTIHHAGQFGMYDGLKTVSCHYSSRDQAIVKDVTNHELLVSVTT

Lotgi1|235548 398 LTEETALTEYQPVSFQLQGKNGNPMPVPVNL-GDELRIYIPLADTGRYTK

GIGA2_CRAGI 225 LDMNGE-----PVQALAANESIILQFEPVGIP-DVRGVMVEY--------

ELDP1_PINMA 219 RDGASS-----NVQATTQSAPISLLFSPQNIP-DVKGAMVDY--------

ELDP2_PINMA 219 SDGNTQ-----NIQEIQTNDVIHLTFNPVNLPGGYKGVKILD--------

Lotgi1|235548 447 LKITELQTNNGMVEQDLVMETLIFNGC-----LTDIGEALVTGDISSDPA

GIGA2_CRAGI 261 LEVYSINANS----NEVVSKTIIENGCVLRTAQQHLEIPIRNYSEMXRQG

ELDP1_PINMA 255 LEVYSINSTS----KEYKSVVAVKNGC----AQKNEYNVAFSNLDELDPA

ELDP2_PINMA 255 LEMYSVQW------NEVNSILLLKDQC---MTQKADELGYSVSN-----E

Lotgi1|235548 491 --IPAIIINFMAFRLRGSPQVK--FDARVQVCEGTDTSCDSVVCPSPPQ/

GIGA2_CRAGI 308 TSWVARSAMR-AFILLPGDH-----------CYSSLDYASVPEVPR

ELDP1_PINMA 297 TSKWIGLVKMQAFIIFEN--EPILFNYRLRFCP---DRCTTPTCAAPXV/

ELDP2_PINMA 293 VDGYSGRAILKAIPLFENVQASVYFNYRLRFCR---NRCKIKSCPSQSP/

**----------------------------------------------------------------------------------------------------------------**

Peptides sequenced by MS/MS are shown in red. The gigasin-2 sequence is from [16] (P86785), the ELDP sequences are from [17] (P86953 and P86954).

**Figure C**

**Sequence comparison of Lotgi1|238082 to nacrein-like protein**

Lotgi1|238082 1 MKLQGAGCVVAAVLGALFIVNVESHFHKPELQLCKAFGEPCISYDVRSTI

MANL_MYTCA 1 ---------------------------RGPKNWCKVH—-PCWTT------

Lotgi1|238082 51 GPRCWFKLEFPREKCCNENGKRQSPIDIPDVKSIYKVPQKLRYSSR-KFV

MANL_MYTCA 16 ---CGSQM-------------RQSPININTNQTIYKRYPRLKVENVHKRV

Lotgi1|238082 100 -GHLENTGIQPAFK-RKVGADKVYLE-GIGSPVGKRYFIENVHFHVGVRH

MANL_MYTCA 50 IATIRNNGHAPYFEVHEKFDDEIVLRNVPERPRRKEYNFAQLHVQLG-RD

Lotgi1|238082 147 KERQTENTLNGRSFDGEAHIVHIREDFGDLKEAANHPQGLLVISIFL---

MANL_MYTCA 99 EKEGSEHSIDNKFKPMEAQMVFYDKDYEDVLEAKSKKNGLVVISVMIEVY

Lotgi1|238082 194 --STSKGERRRDGFDDLIEMIQDVQEFEEE------DGPCANVKIPDIFK

MANL_MYTCA 149 GRSKEHDDCACDGETCTVRYVRKLSKLMEKYYEKVRRYPLVSIN-PHFLT

Lotgi1|238082 236 FKQLIPFHPVWPICKKTFPVAD---DSDNSGSGVVCNFYLPNGLCGEKKE

MANL_MYTCA 198 FIKL-PRKCWYNKCGRT-PSPDFIEKKCEKEEPETRPFFVFEG-------

Lotgi1|238082 283 SKINPNELLA-DDPEYYVFNGGLTTPPCSESVLWLVAKQPRKVSVFYPYV

MANL_MYTCA 238 --ITPLDVIPYDTNRFYTYAGSLTSPPCYETVQWVVFKCPIKVSS-KAFR

Lotgi1|238082 332 VRNMETQREGEIIGDFGNLRPLQDLNDRPVFLVRFRLKRNWEHGDTAAND

MANL_MYTCA 286 MLQLVQDSHLDPLEKLGVRRPLQ--TNKNVIVYRNHLK

Lotgi1|238082 382 NDAMDSPFSVLGIN

**----------------------------------------------------------------------------------------------------------**

Peptides sequenced by MS/MS are shown in red. The nacrein-like sequence MANL_MYTCA was submitted to EMBL/GenBank/DDBJ databases (P86856) by A. Gracey, J. Grimwood, J. Schmutz and R.M. Myers (2008).

**Figure D**

**Alignment of Tyrosinase sequences**

Lotgi1|166196 1 MRIALSLLLLLSIVTDVEPLIREAPLPKQLKECYQKYSRKSLASVVGKSL

TYRO_PINMA 1 MNTMTLLGKVFLLQFLIGVGFCMLMQDPKRNDTKGTYAACFRSQPQGNEP

Q287T6_PINFU 1 MKMNLSNREVVIFLLLAACTSAALLGDKYNVPPECMEEVIFDYDSPKDNS

A1IHF1_PINFU 1 MKKLWALAASLPLLLCVHCIKEKEILKESYKQKCMKNAVYDFNSTNPTTL

Lotgi1|166196 51 --CWYCETSLRGRMNPPAEPMVLPNRR-DY-----RRLAEPLIN--RRVK

TYRO_PINMA 51 ASP-DCLKAFMAYAEDMKNIFHFTKEQINYLWSLERETQSLLHN-HRRRK

Q287T6_PINFU 51 TLNKDCVKFVSDSYRKLQQLINGTDDDINYIRSLTREGMALLYPGSGREK

A1IHF1_PINFU 51 EPK--CATLFGHEYSDIKNFLKFDDQQMNYILSLERAMMRTQHRNNKRHK

Lotgi1|166196 91 RQAGGST----CIRKEYRMLTSAERDNYHNAINALKQDTTMTPNMYDAVA

TYRO_PINMA 99 RQAVYLPVRKEC-RLLSELERQNLFY----TVRSLKMDTS-NPNEYDTLA

Q287T6_PINFU 101 RQAALRA-RREC-RSLTSEEWRRLA----NAIRRLKFDPG---NRFDTMA

A1IHF1_PINFU 99 RQAMMRP-RQEC-RTLSDPDRNALF----GAIVTLKQPFSGMSR-YNTLA

Lotgi1|166196 137 MFHVG-DASVRAHGGPGFLGWHRMYLVMYERALQSKVPGV--CIPYIDNT

TYRO_PINMA 143 NLHRG-AVQPHAHDGSNFLGWHRVYLMYYERALRRIRGDVTLCFWDTTME

Q287T6_PINFU 142 RIHAMPAVIANAHDGSSILGWHRVFLYLFENALRRKVPGVVLCYWDSTID

A1IHF1_PINFU 142 AMHNL-QAFGNAHNGPNFLGWHRVYLNMYEEALQEIRPGVALCYWDSTLD

Lotgi1|166196 184 IEAELGDDG-SYLWSDEFLGTPNGVVTSGPFANWNTPIG-----ELTRNV

TYRO_PINMA 192 FNLGMDNWEYTAVFSSDFFGNRRGQVITGPFRDWPLPPGLTESDYLYRNM

Q287T6_PINFU 192 YLIPGPGQAQSSSFSHNMFGNSRGLVRTGPFANFPTPWG-----PLRRNF

A1IHF1_PINFU 191 YLMPGDSQRRTVAFSDELFGNGRGAVINSQFANWRL----SDNTPLRRMI

Lotgi1|166196 228 GNQAFPMDKDILNDIMSR-GRIED---IVSPTAELEH-------------

TYRO_PINMA 242 TRGRGMPFDSRAASSIFYNPNTIIHSTITWEG--------FGFDTITNSQ

Q287T6_PINFU 237 GGEGGSLMRPHVVDMIASDPRIRSHGQIV-DG-----QGATGF-IDSMT-

A1IHF1_PINFU 241 GENNSSLTRPGIVDLILTDPRINRHRWIVNEGSRFNQSPRFGF-IDPDS-

Lotgi1|166196 261 ------DIEYHHGSYHIHVGGLMESIDTASFDPVFFMHHAYIDYVWEQFR

TYRO_PINMA 284 GQTRNITIEGEHNNVHNWVGGAMGFLDPAPQDPIFFFHHCYIDYVWERFR

Q287T6_PINFU 279 GQRT--SLEAEHNNAHVAVGALMAVIPNAAWDPLFYFHHCYIDYVWQLFR

A1IHF1_PINFU 285 GMRH--SWEREHDNTHVWVGGIMVNVERSPEDPVFWFHHLYIDYVWELFR

Lotgi1|166196 305 QKTLAAGG-DPTR-YPESNDLPLHTGDTVINVIQLPTGN-VTVTQRDMYA

TYRO_PINMA 334 EKMRRYFR-DPTTDYPGHGNETLHDANYPM------IGF-EWYRNIDGYS

Q287T6_PINFU 327 RKLRNRLGIDPARDYLGHG-GPAHAPNAPL------LGLIPGWRNVHGYS

A1IHF1_PINFU 333 RKIDPMDRFDLRTDYPMDSVNEQHRAFQTM------AGF-PAYRNIDGYH

Lotgi1|166196 352 -TLTDYEYQP--SPECSRTNPDCGSI---YLAC------NRTSYRCYPVN

TYRO_PINMA 376 DYFTQNVYRYE-SPTCQ----ACYYS--PYTVC------GQGNQ-CI-AR

Q287T6_PINFU 370 NVFTQRVYRYHFHPVCGN---GCSGSTRRLLYCPG-G--GSRYRRCV---

A1IHF1_PINFU 376 NFF-RRMYAPH--PRCSN---NCGGS--RFLRCPDIGPMGNPDRRCVSLA

Lotgi1_166196 388 PSLQPPVNPGPPINP-GPPVNPGPPVNPGPPVNPGPPVNPGPPVDPPT/

TYRO_PINMA 409 MNYPGTEIEEGPQVPNGPVAAFSVAGGTMMMSASNGRGFIATSNSE

Q287T6_PINFU 411 ----SNTMPGRAQPPALSIAGRSAEEKFKTVYDDPDIAS

A1IHF1_PINFU 416 ID--SDVVPAAAASPAAAMAGFGASRAGFAAFGGPAAMASGGAARVSL/

-----------------------------------------------------------------------------------------------------------------

Peptides sequenced by MS/MS are shown in red. The sequence *Pinctada maxima* tyrosinase (TYRO_PINMA, P86952) is from [17], the *Pinctada fucata* sequences are from [53] (Q287T6) and [52] (A1IHF1, Pfty2).

**Figure E**

**Comparison of Lotgi1|231009 to UP2**

Lotgi1|231009 1 ----------------------------------------MANIKI---W

UP2_HALAI 1 PLGAATSNIPPQYARSTLQPTGLTSRAQSYPTNTNPGPSAKGNLVLPLNW

Lotgi1|231009 8 ILLCMFLAFVAVNQAQL----TGLANLVAGPKGRMLKMLA--YRSPALQR

UP2_HALAI 51 QLLNSPASQIPTQSTTTFRSNPPLPPVVPGRRNTSPFFFPKPTRPLSFRQ

Lotgi1_231009 52 GLATLQDMKIAKRLGCH---RSLDNESPLRAILPMGCTTQKDICPYTRPM

UP2_HALAI 101 ILDFLGRIRATKELDCKTVSEALSLNLP-KFYYPLSC---DDKCP---PP

Lotgi1_231009 99 TKCLQVGIIGMCCPYYVSSNSIKSAKMYAKWSKFSEIMA

UP2_HALAI 143 SVCRHVGLVGFCCPPHVTDQLIWMVGLAERFKVLGG

**---------------------------------------------------------------------------------------------------------------**

Peptides sequenced by MS/MS are shown in red. The sequence of Uncharacterized protein 2 (UP2) was contributed by [17]. The Fasta E value was 2.9, indicating a low significance for this match although it was the best match of only two matches reported.

**Figure F**

**Alignment of osteonectin sequences**

Lotg1|176394 1 ----------MTIGMTEEARMRKWIVALLLGLVFTAVYVRADEDEDDDEE

Lotg1|109908 --------------------------------------------------

F2Z9K2_HALDI 1 ----MRHLLLVALLAVIFSAVFAKGGRRQQRQDVQSDIADDVEEGGEEES

F2Z9K1_PINFU 1 --------------------MKWILALFLLGLVWSALAQYDLTDVDEEGD

SPRC_HUMAN 1 MRAWIFFLLCLAGRALAAPQQEALPDETEVVEETVAEVTEVSVGANPVQV

Lotg1|176394 41 VEEEEIDVAAVEAGKTTLVNPCEKKRCRRGEQ-CIVDEKRQPSCVCYQN-

Lotg1|109908 1 -----------------ITDPCEKKRCRRGEQ-CIVDEKRQPSCVCYQN-

F2Z9K2_HALDI 47 DENEEVDVYAEEKRLRMRIDLCKKKKCYRGE-VCRLDNRQQAECVCLHE-

F2Z9K1_PINFU 31 DDVDDNVQPVDDNNQGNRKNPCNFKECKRRGQTCILTPNNKAKCVCREE-

SPRC_HUMAN 51 EVGEFDDGAEETEEEVVAENPCQNHHCKHGK-VCELDENNTPMCVCQDPT

Lotg1|176394 89 -CEEETDERYWVCSTKNITYKSDCLLDREHCLCRRKDAACKNLAEKKVHL

Lotg1|109908 32 -CEEETDERYWVCSTKNITYKSDCLLDREHCLCRRKDAACKNLAEKKVHL

F2Z9K2_HALDI 95 -CEPEVDPRYHVCSTKNQTYESECELDRDHCLCKTKQPGCSNARLNKIQL

F2Z9K1_PINFU 80 -CKIDPVPRHMVCSVKNMTFDSECHLDREYCMCKSMK-ACSNAEAKKFRL

SPRC_HUMAN 100 SCPAPIGEFEKVCSNDNKTFDSSCHFFATKCTLEGTKKGHKLHLDYIGPC

Lotg1|176394 138 DYYGGCREKIQNLND/

Lotg1|109908 81 DYYGGCRDLTACPKDEFQEFPNRLREWLFIVMKQLAAR-EELHEYLDLLE

F2Z9K2_HALDI 144 DYFGGCRKLTKCPDDEFEEFPIRMKEWLFLVMKQLASR-DELGEYIDLLD

F2Z9K1_PINFU 128 DYYGECKELTRCEDLEMKQFPDRMSNWTYVVMKEMARCHQLDTEYLDLLK

SPRC_HUMAN 150 KYIPPCLDSELTEFPLRMRDWLKNVLVTLYERDEDNNLLTEKQKLRVKKI

Lotg1|109908 130 SAKSDANHTDALV--------------------WKFCDLDQNPQDRRVSR

F2Z9K2_HALDI 193 KARNDANHTEAVL--------------------WKFCDLDSSPQDRQVSR

F2Z9K1_PINFU 178 KATADDHHTDAIL--------------------WKFCDLDIRPHDRKVSR

SPRC_HUMAN 200 HENEKRLEAGDHPVELLARDFEKNYNMYIFPVHWQFGQLDQHPIDGYLSH

Lotg1|109908 160 RELQHTIQSLKAMEHCLVPFLNDCDANNDRRITLREWGGCLNADLSK

F2Z9K2_HALDI 223 RELQYIVQSLKAWEHCLVPFLSMCDQDSNRKITLTEWGARLGVNSKKISD

F2Z9K1_PINFU 208 RELLFIIASVKPMEHCLVP----CLNLDPVHIEDKCKDIQSRRQ

SPRC_HUMAN 250 TELAPLRAPLIPMEHCTTRFFETCDLDNDKYIALDEWAGCFGIKQKDIDK

Lotg1|109908 -

F2Z9K2_HALDI 173 KCIDIRARAKRH

F2Z9K1_PINFU -

SPRC_HUMAN 300 DLVI

**----------------------------------------------------------------------------------------------------------------**

Peptides sequenced by MS/MS are shown in red. Lotgi1|109908 contained the C-terminus of the protein, the N-terminus was identified in the first 135 amino acids of Lotgi1|176394. *Haliotis discus* and *Pinctada fucata* sequences (UniprotKB/TrEMBL accessions F2Z9K1_PINFU and F2Z9K2_HALDI) were submitted to databases by H. Miyamoto and F. Asada. The sequence of human osteonectin/SPARC/BM-40 is from [77] (P09486).
