# Supplementary material for: In-depth proteomic analysis of a mollusc shell: acid-soluble and acid-insoluble matrix of the limpet Lottia gigantea
Source: Proteome Sci. 2012 Jun 13;10:28. doi: 10.1186/1477-5956-10-28 (PMC3374290; doi:10.1186/1477-5956-10-28)
Supplement: Additional file 16 — Sequence analysis of BMSP-relatedLottiaproteins. Doc-file showing the alignment of BMSP-related protein sequences to Mytilus galloprovincialis BMSP (A) and the domain distribution in these sequences (B). [file 1477-5956-10-28-S16.doc]

**Additional file 16: Sequence analysis of BMSP-related *Lottia* proteins**

**A: Sequence alignments**

Lotgi1|140660 1 VDLYPTSKISGVNCNVPGDVIFLMDASDS

BMSP 201 NMVCQVITTTTSPPLQTQMVTLPSIHVANPTDKVCNVPGDIVFLIDGSNS

Lotgi1|140660 30 IDPADWEREKNFVEILIDSLTIQPDAIHVGMVVYSTFIGDVIGLKPYKAK

BMSP 251 MSNADFRKQKNFVANMIDNFEIGKEFIHVGIVVFSTVIGDIVHLQPSRSK

Lotgi1|140660 80 SRLKTIARNLTQLRDGTDTALGIAEVRKMFRLQGRSNT--PHVAIVITDG

BMSP 301 DLLKILANNLRHPKVGTNTALGIERVRKMMREEGRSFA--PKIMVVVTDG

Lotgi1|140660 128 LSTIPAETVRQAFMAKREGTAMIAVGIGDQVFMDELRDIASSASTLFNVQ

BMSP 349 RSASPALTSLQASLAKAEGLTVIAIGVGSAMFKDELENIASDQGKMFQVT

Lotgi1|140660 178 DFRALQGLVQSLRDII--------------------------LLACSSPA

BMSP 399 RFQDLELIITAMRNLICQSITTTTSTTTTTTTPATINVPYPPGLICDVAA

Lotgi1|140660 202 DVIFVMDGSDSIIPMDWIREKQFVAQLINSFDIGPSAINVGAVVYSSIVG

BMSP 449 DVGFVIDGSKSISSGDWPKGLNFVANLINNLYITPEGIHAGIVVYSTFIN

Lotgi1|140660 252 DVIQLRPFKSKQRIVNMIMSLKQPRFSTNTKLGIAMVSICKCFLKLPKVN

BMSP 499 EKVPLNPFKSKPLLMAMTKSLKQPKQGTNTALGVDTMRQMFRNQGRYDAP

Lotgi1|140660 302 LSRM

BMSP 549 KVMII/

Lotgi1|173138 1 MALAQHPHRFPKVVWAPLGLCDGR

BMSP 799 LQGLSSSERFIHRTDNFNSLEMIMPQIRQQICEIIMKPPGDSFTELCSGC

Lotgi1|173138 24 VMDKGIGYNAYPEDCTKYVQCFQQGNTYRSVIRPCPFGTFWDQNAVTCSN

BMSP 849 LIDKGIGFNPYPGDCTKYVQCWRDNNRVMGAIKSCPFGQFWDRVAMACQP

Lotgi1|173138 74 AANVICRNDLCRTMPDYVIYAHSGPGCRPHWMCLGQRSLPQCCPAGSRFI

BMSP 899 SLSVQCPADVCRHSPDGFTYGMKNKGCRAYWLCVNGHSVGSCCPEGSYYV

Lotgi1|173138 124 DKVGCIVDPMCMESCMSHHHENHTSAECMLKTHADKKFYLETIAGFGEIA

BMSP 949 EGMGCMRGKACKEPCPPDGGIILT-PDCSKEEHWDQRYFIEKVPFYGKVV

Lotgi1|173138 174 RPCAPGSIFSEKQCTCISAPDLNLHSQGELSYASVTFHSTNALTFFHFHF

BMSP 998 RPCPPGTVYSTSYCDCVVKGTNGIIPILPGDKCRAKAHFNFDVDMKDKSG

Lotgi1|173138 224 SSMAAEDN MFNGKSQVNLWRFAGVEFGPELVLKFRYRM--

BMSP 1048 GSVPISYRNVQRTRYGTAHFTGNGEMNLWKNRNINVNNKLVLRFKFKLDR

Lotgi1|238526 31 WTPATDWTP-----GSGG--TWTWDASSNSWINSTSSAGSWMSGMDGVWI

BMSP 1098 WTDRFDYGGVIVDMGKGGDVHWKYDIESGWTRNITVG-GGWDIRNLVKLF

Lotgi1|238526 74 ------GTNDQTGQQWLGVGNPGGHWVGTVDSGMTGNG-MGYDFWNSLST

BMSP 1147 RTLVSSRTEPEMKQNMWKVANSMDFKTLLYQLGFVPNTSSGMQLLRLLMN

Lotgi1|238526 117 GTG---VWPGMWQVGNGMNGVGLGSMWTGAFGGNGISGTSSFSGSGNWVG

BMSP 1197 GNGADILQKIVIALEKQKSPYDMRMTLKTMLSRPEMRTWILQNTPKQYID

Lotgi1|238526 164 GSSSSTGSWSGTFGNGNGGTWSG---VARTGSGTWSGTTTNNQLVTGTWA

BMSP 1247 ITMKNWNRFLESLSLPENQWKTGKITWYKPADKIGTGSMSSIAIIADLWR

Lotgi1|238526 211 DMSGAGTYGIWNVTSGPWLGTSGVWSTNGNGGGTWIVTQVADMTNSG---

BMSP 1297 TFARAMGMDTRDWQNLLRITLGTDIIGEGGRVDVEFINEMKNVLGRNNTI

Lotgi1|238526 258 -GSWSNTGRYDSSWNMNTQGTGNTGEVWTGTLDLGSLDTGTSWSRGSETE

BMSP 1347 LQAWHDFLRGKNVR-LDFEGKNPTTSWWEKTFGIGRGMAPIPGIDRGMSV

Lotgi1|238526 307 TRGTSTWNNVQGSGTNIQGGTSSTSTSTGNTGLWTDGTGETINTNVDKSG

BMSP 1396 GIVADLWRKFIQEAQLPESMKQKLTWTFGGPDATEDDLFKEINTVLGDDN

Lotgi1|238526 357 IN------IQGATTGGNTDSVDTSKNQISDN-IDITKVGIDAGAKGINTG

BMSP 1446 LKNKWIHFIQGHYAHGGPGKTEAQLWKPGPRGELFWKIIFGQGKDGSGTG

Lotgi1|238526 400 PTGGTNQINRMYSLDTVNFLGGKWDNGNWVSDSSDTGPQTSVDTSWTFEK

BMSP 1496 PGTDGSQTGTGQTSVDIKKLADLWR—-TFVTQSKLEGPQ-WKDLLI-FTN

Lotgi1|238526 450 EISGGQTGPTKPVVNQPRRNRWQIVGSGKPNIPVIDTPKTGGSIDSIFNV

BMSP 1542 TDKPGSKNINVKLLLKEMKN—-VLMADDNVDTAWLKYLQDNG--------

Lotgi1|238526 500 -DGQWVVQPNKNQGQPAVRVEGGRWVKEPTNPSSTTTDSQGRSFITGSGM

BMSP 1582 IDKSWFLRILFSDQGGPFNENWVYWYLYRNKEYAGIGNSTGGIGVGGS--

Lotgi1|238526 549 WKIVGPDGRIIRSGVGNSIPANVINELMGNTRSVGWTKQLEWTIIGPNGQ

BMSP 1630 ----GGGGQTNKNQIIGLWRDFVTQSKLG----PEWNGMLTWTLGSGNNG

Lotgi1|238526 599 LLRSGTGQIPQNLRNTGTVLAGG-----FFDVNLG--DVSQWVIISGDGQ

BMSP 1672 GPGEYMEWIYEIISVlGNKEMRGRWLNFLNAKNVGNEWINWFMVISGDSR

Lotgi1|238526 642 ITKHGIGSTPNVQIPQGSLLMKIIWSSGNQ-QWTIAKPDGTVIKSGTGNF

BMSP 1722 GSFEVDQSGLNLVMSLWKRFIGALNLNGQGGQSGQNWNQKLIWTLGGGLT

Lotgi1|238526 691 PQTLDLSNLLNQVKPTGRFGMGQTGATVVTGSGGGGRWIIGGNQAMARQP

BMSP 1772 DRYIDWSNEVVSVLQN—-TTIRDHWFNYLQQNNAGNVWI-------NWFR

Lotgi1|238526 741 SVMKIGGGQVKIGSGQPSVVKIGSGQPSIMKIGGGQVKIGSGQPSVVKIG

BMSP 1813 MILDVGSTSSSSGNWIWHYLSSGMSVP-LDILANLWIQFVFDR----NLG

Lotgi1|238526 791 SGPLPMNVGGSSQWTVSGSGTSGPSSIDINKIIGAIGGKIPGSSVGSSGP

BMSP 1858 YGQWADSLWWTLNVIPGAISADEYMHLFNEMTILMMDEMIRNDWITFLHG

Lotgi1|238526 841 RQGSSGTATFVSNSGTSGQQGGSGSSNIRGVSSVAGSGRVDSTPLGSRGV

BMSP 1908 NDITASWSSTAGAGMTGGTTAESLDAILNSLSGGTGSGSYGTSSGSSSGG

Lotgi1|238526 891 TDLERGSFDVTVDKTNIGPLDYNLRAIKMRSKRSLSRPQAIISNCNGVNG

BMSP 1958 SDFFWG---LGGDWDMDGGFSRKRRSAHRQKRAIVDKMAHVALVGNCGNN

Lotgi1|238526 941 EGPSIMVSGGPDYVTFSIKTSNTVRPAQLTIPARPGYNDVSMIYDGQNLK

BMSP 2005 VKPSISITANEQNVKMSLLTQNI--PAELEIPLIQGWNEVTMVYDGKNLH

Lotgi1|238526 991 KAKVN---DIARSIPLTGKIEMRQAGLLFGACNGYANFRGEVDDFEMYEC

BMSP 2053 GTVQNWQGKKHKKTPLTGNIVQRKG-LTFGACDKYPRFAGQLDDILLYNC

Lotgi1|238526 1038 IPPFWG

BMSP 2102 VPLALKKEYP

**B: Domain structures and alignment**


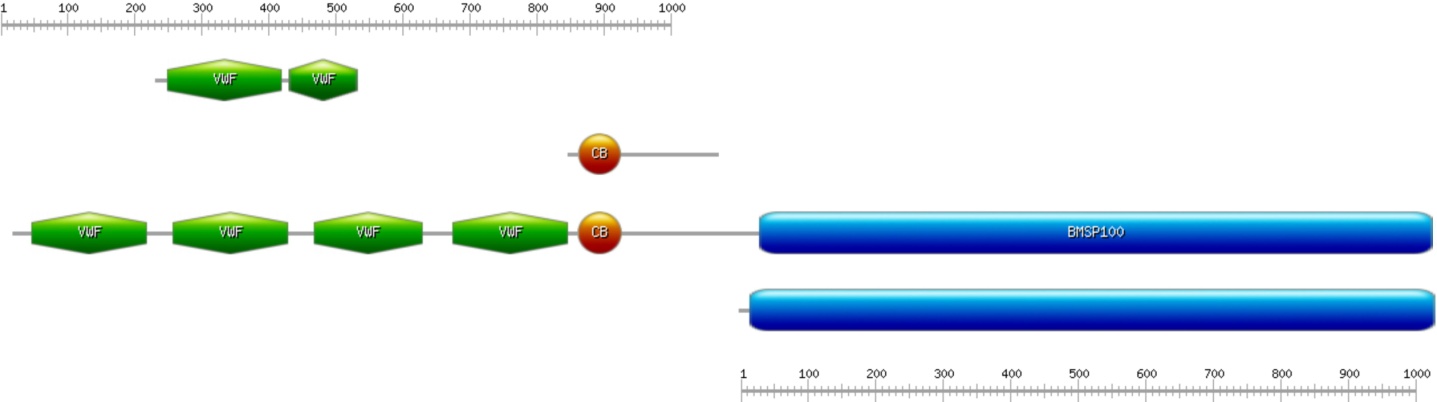


**A**, MS/MS-sequenced peptides are shown in red. The chitin-binding domain identified in the original publication [48] is underlined. The *Mytilus galloprovincialis* sequence is from [48] (G1UCX0_MYTGA). **B**, domain borders were determined with Prosite (<http://prosite.expasy.org/>), the drawing was prepared with the help of Prosite MyDomains (<http://prosite.expasy.org/cgi-bin/prosite/mydomains/>). The proteins from top to bottom: Lotgi1|140660, Lotgi1|173138, BMSP, Lotgi1|238526. VWA, von Willebrand type A; CB, chitin-binding.
