# Supplementary material for: In-depth proteomic analysis of a mollusc shell: acid-soluble and acid-insoluble matrix of the limpet Lottia gigantea
Source: Proteome Sci. 2012 Jun 13;10:28. doi: 10.1186/1477-5956-10-28 (PMC3374290; doi:10.1186/1477-5956-10-28)
Supplement: Additional file 17 — Sequence analysis of Pif-relatedLottiaproteins. Doc-file showing the alignment of Pif-related protein sequences to Pinctada fucata Pif (A) and the domain distribution in these sequences (B). [file 1477-5956-10-28-S17.doc]

**Additional file 17: Sequence analysis of Pif-related *Lottia* proteins**

**A: Sequence alignments**

Lotgi1|228264 1 MVCKL-FLAFLLALTVSVYSDEHQNVQYVAPISSYTECKS-LLDVVVVVD

Lotgi1|232022 1 MGWLCIVRRGVQKVSFSNLKGLYAEN----------EQCEHLLDVVAVID

PIF_PINFU 1 MVQPSIRVVFLLTAVFCVGVKS-------------DECKT--ADVVVNVD

Lotgi1|228264 49 GSDSIAADDFVTLKLALESLVLDLNVRPDNTRFGVVLYSSTIAGKIDISG

Lotgi1|232022 41 GSETIFPDEFQTIREVMGKFVRQLGVEENNKHFGAVVYSSAVSEVIEMSG

PIF_PINFU 36 ASDDVSDQDFDKLKRAMLMMVRGLSIDDNQIRLGMVTYGSEVCDSIPLQG

Lotgi1|228264 99 NAGHIIPGIRALPHPRDGTNTALAIAEMNDMVAAQRRPGVPV----VGVV

Lotgi1|232022 91 DQEKLVEAMLNMDHPRTGTRTDLGIEEMINVFIRKGRSDVPK----AGIV

PIF_PINFU 86 DRLDLARTIRYMKKPTGPSKPFKGMGEARRMFSSRGRYNVPHITMNLGGD

Lotgi1|239574 276 /AVGNIPTIQLPGSGIIIAR--------------PDSWTPTTTTVAPTTT

Lotgi1|228264 145 ITDGISKDQAATAQQAAIARNQGINMFAIGVGINVDTTELKSIASNDQQV

Lotgi1|232022 137 ITDGASHEKELTLKQAQIAKDMGVNMFAIGIGARIMDAELKGIASKPDQA

PIF_PINFU 136 IVDTEVKDLMDETDK---ARDEDIKVMAIGLGAKVDRDEIESIAYDRDQA

Lotgi1|239574 311 TTVAPTTTTSQPAPTTTVALTTTTVAPTTTTTTVAPITTTTKAATTTTPT

Lotgi1|228264 195 LTTVNFNQLGSLLSNFIQVVCPTTTTSTTTTTTTTTTPAPTTSTTVPTTT

Lotgi1|232022 187 YVIEDFEAFAKLIASLAIQICPTTTTTTTTTTTTPKPTTQKPTTRAPTPE

PIF_PINFU 183 YFMDDEDDLIRKVKEIPDYLCKIIKAKKPKVSGGKKSKPAKKVDNGPAGK

Lotgi1|239574 361 TKVAPTTTTTTTQKPVPYKNVIDGECNRCIYRNGVGFLSHTSDCTKFFKC

Lotgi1|228264 245 TTVK-----------------PD-PCANCKMSNGIGFNPHPTDCDKYFQC

Lotgi1|232022 237 SAP-DATPESTSPPIK-----PDGPCSGCRMSNGIGYNSHE-ECHKFVQC

PIF_PINFU 233 SPGFDALKQSDDKSDKAKKVEVKELCDDAEWVDGVGYGSVPTRCEDFVMC

Lotgi1|239574 411 QRLSNGGFRAAELQCPFGLYWNNEIFSCDYPRNTNCTNHPCTNTNTRYAE

Lotgi1|228264 277 EFSLEGLVNSVLRQCGQGLFWDQDLLTCNYPAAVQCRADPCQNYHISSYK

Lotgi1|232022 280 FFRDGGLTGFFVQTCGTGTFWNQDKLTCDHMSKINCPNDRCALVKELKYY

PIF_PINFU 283 Q-NVSGSLRKTLKSCPFGQYWSKRQTSCVLTEDEDCSDDLCKTMLLPSRE

Lotgi1|239574 461 MT-GHCAGYWRCDWATPVAYCCPQGHRFQQSSQLCEVDRTRTCRDDCSGP

Lotgi1|228264 327 KA-GNCREYYSCSNGTSMPECCKKGFAYVSGQ—-CVPSYN--CNAHCKGD

Lotgi1|232022 330 KLGGNCAEYIECVNGRSQPNKCRVGYSFVEGDEICLKDAS--CTTGGSIG

PIF_PINFU 332 YD-VSCRAYWKCEKGKSVARCCPSGMAYEPGKG-CVLDLD--CDEECPPK

Lotgi1|239574 509 QQKPSGVVEKL------------CDKRSVSDSKTAFEQRVPVNRWIRLDC

Lotgi1|228264 372 FINPY------------------CEMRAVSDDISSYEQFVRGVGWVRKPC

Lotgi1|232022 378 GNNE-------------------CNFWSVPGTKCYYYWTLGNQGWT-MPC

PIF_PINFU 378 NDGDDDDDSSDEDDDDEIEYNPNCPLRPIKGHPEK**F**KQHTGDDNWEDFDC

Lotgi1|239574 548 APSTGFNPKTCLCSDRVNAPVTQ-----KTSNKDCDPMLHISFNNGVRDE

Lotgi1|228264 403 APGSAFSPVECSCTVA--------IDPLPINAGECKAEVYIPFDDDVAID

Lotgi1|232022 409 PRGTRFAQRQCGCVYASPNR--------------CRPRSCTPLVDIDFNG

PIF_PINFU 428 APGTLFSARDCACSILGTAKKDDKNDDGGDAHKVCEPELYLPFCDDLH-D

Lotgi1|239574 593 SRHRF-WIENVGVTSKA--GVGLFNGNNKLLVNRFANAPLGRDLVIEVVY

Lotgi1|228264 447 KSGNGNYVENEGVFVI--GGKGYFNGTSGLRIPRFSNIEFGSKVVITMRY

Lotgi1|232022 447 AEP-GEY—DNYGAVVK--GGLAYFDGNSNIKILRLSNVEFTTKLSILIRY

PIF_PINFU 479 YSGKETHVENEGDAVIIENGKAYFNGRAGLKIPRFSGVPYGKSVFIKMKY

Lotgi1|239574 640 EPADRRRDEVLVSNGDCGVKPSLYIVTGPTGVTFSVKTTRSSTPSVVTVP

Lotgi1|228264 493 KAESIYGSQGLISNGDCGKPGSLLVAIDNTNTLFGLQTVSGTAGIVTIPS

Lotgi1|232022 437 KPFNSNNTEAVLSNGDCGEEATINLINNGAVASLGVINAENQRAALHDIT

PIF_PINFU 529 KEDEDDDKNKNDDDKKLRMKRDERSRKDYLKAILKRDDRKDKTDDTKGRR

Lotgi1|239574 690 ISMPKGLIKARLSLANGRLTGEVGGLSKSTPASGSVELRKSSLIIGSGDG

Lotgi1|228264 543 ANGWNEIIYQVEGDVLTGSVNGNSAHKTIDGAVK--RSQCALQVGRATHL

Lotgi1|232022 487 EAEWREIEYTFENGEIKSVMNGKSGLKSFAISDLPRRSQCAFQLGHSEGF

PIF_PINFU 579 IIDRNDIIDDRRGRRKDDRKDGGRDDGKDGRRDDRKDNRLDDIKDKNDEP

Lotgi1|239574 740 MKKFD-GIMDDVKMFFCQNDGK

Lotgi1|228264 591 SNFR--GYVDELTVYLC

Lotgi1|232022 537 KDFR--GWIDYIVVSTC

PIF_PINFU 627 MTLISNGECDNFELNDCFEKPS/

**B: Domain structures and alignment**


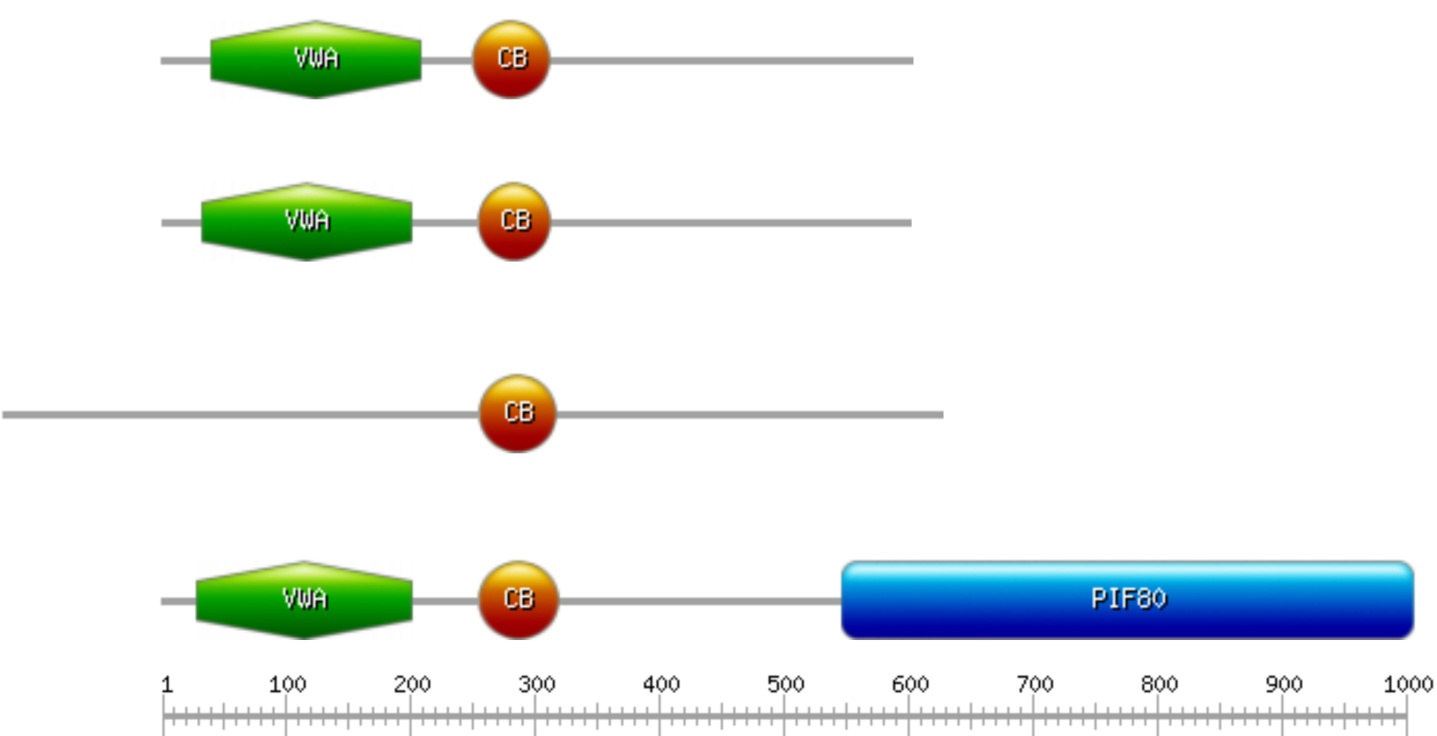


**A**, MS/MS-sequenced peptides are shown in red. The chitin-binding domain identified in the original publication [47] is underlined. The *Mytilus galloprovincialis* sequence is from [47] (C7G0B5). **B**, domain borders were determined with Prosite (<http://prosite.expasy.org/>), the drawing was prepared with the help of Prosite MyDomains (<http://prosite.expasy.org/cgi-bin/prosite/mydomains/>). The proteins are from top to bottom: Lotgi1|228264, Lotgi1, Lotgi1|232022, Lotgi1|239574, Pif.
